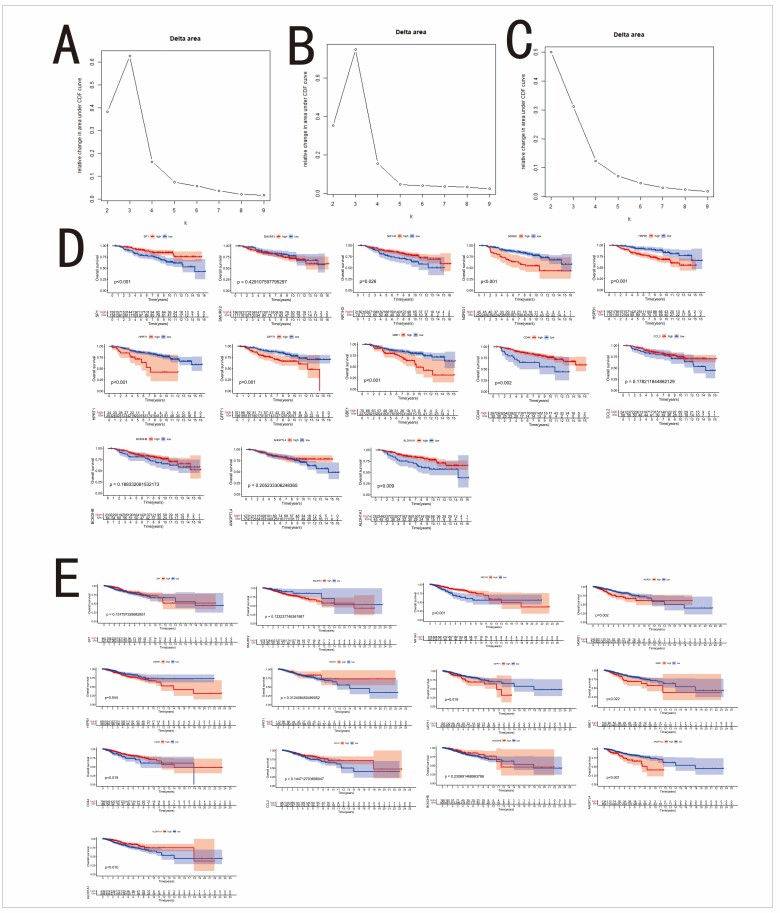# The crosstalk between metabolic reprogramming and epithelial-mesenchymal transition and their synergistic roles in distant metastasis in breast cancer

**DOI:** 10.1097/MD.0000000000038462

**Published:** 2024-06-14

**Authors:** Liyan Yu, Yongni Chen, Yingyu Chen, Kangwei Luo

**Affiliations:** a Department of Breast Surgery, Guangdong Medical University Affiliated Hospital, Zhanjiang, P.R. China.

**Keywords:** breast cancer, epithelial-mesenchymal transition, metabolic reprogramming, prognosis, tumor microenvironment

## Abstract

**Background:**

Metabolic reprogramming (MR) and epithelial-mesenchymal transition (EMT) are crucial phenomena involved in the distant metastasis of breast cancer (BRCA). This study aims to assess the risk of distant metastasis in BRCA patients based on MR and EMT processes and investigate their underlying mechanisms.

**Methods:**

Gene sets related to EMT and MR were downloaded. MR-related genes (MRG) and EMT-related genes (ERG) were obtained. Principal Component Analysis method was used to define the EMT Potential Index (EPI) and MR Potential Index (MPI) to quantify the EMT and MR levels in each tumor tissue. A linear scoring model, the Metastasis Score, was derived using the union of MRGs and ERGs to evaluate the risk of distant metastasis/recurrence in BRCA. The Metastasis Score was then validated in multiple datasets. Additionally, our study explored the underlying mechanism of the Metastasis Score and its association with tumor immunity, focusing on *HPRT1* gene expression in breast cancer tissues of transfer and untransferred groups using experimental methods.

**Results:**

A total of 59 MRGs and 30 ERGs were identified in the present study. Stratifying the dataset based on EPI and MPI revealed significantly lower survival rates (*P* < .05) in the MPI_high and EPI_high groups. Kaplan–Meier analysis indicated the lowest survival rate in the EPI-high + MPI-high group. The Metastasis Score demonstrated its ability to distinguish prognoses in GSE2034, GSE17705, and TCGA-BRCA datasets. Additionally, differences in mutated genes were found between the high- and the low-Metastasis Score groups, displaying significant associations with immune cell infiltration and anti-tumor immune status. Notably, the 13 genes included in the Metastasis Score showed a strong association with prognosis and tumor immunity. Immunohistochemistry and western blot results revealed high expression of the HPRT1 gene in the transfer group.

**Conclusion:**

This study established the Metastasis Score as a reliable tool for evaluating the risk of distant metastasis/recurrence in BRCA patients. Additionally, we identified key genes involved in MR and EMT crosstalk, offering valuable insights into their roles in tumor immunity and other relevant aspects.

## 1. Introduction

Breast cancer (BRCA), the most common malignant tumor in women worldwide, is the second leading cause of cancer-related deaths among women. Globally, an estimated 2.3 million new cases^[1]^ and 685,000 deaths^[2]^ occurred in 2020. In 2022, approximately 290,560 new cases and 43,780 deaths were reported in the United States alone.^[3]^ It is now understood that breast cancer exhibits significant intra and inter-tumor heterogeneity. However, once distant metastasis occurs, the chances of long-term survival for patients decrease drastically. Metastatic breast cancer is rarely curable, and treatment generally focuses on palliative care. According to the American Cancer Society, stage IV patients have a 5-year relative survival rate of 26%, compared to nearly 100% for stage I patients.^[2]^ Based on the presence or absence of estrogen receptors (ER), progesterone receptors (PR), and human epidermal growth factor receptor 2 (ERBB2; formerly HER2) molecular markers, breast cancer can be classified into 3 main subtypes: hormone receptor-positive/ERBB2-negative (70% of patients), ERBB2-positive (15–20%), and triple-negative (lacking all 3 standard molecular markers; 15%). While early-stage breast cancer often has a favorable prognosis after comprehensive treatment (including surgery), the therapeutic outcomes for intermediate and late-stage breast cancer are limited. In this respect, triple-negative breast cancer has a median overall survival of about 1 year.^[4]^ Therefore, assessing the risk of distant metastasis and recurrence of breast cancer is particularly important, which provide a basis for clinical decisions in treatments for breast cancer patients.

Epithelial-mesenchymal transition (EMT) is a process by which epithelial cells transform into cells with a mesenchymal phenotype. During EMT, tumor cells undergo significant changes, including the loss of tight junctions, disruption of apical-basal polarity, and cytoskeletal remodeling. These alterations collectively promote cell migration from the primary tumor site, invasion into adjacent tissues, survival within the circulatory system, and ultimately, the formation of metastatic colonies at distant locations.^[5]^ Research by Mehrdad Hashemi et al^[6]^ reported that EMT induces migratory behavior in breast tumor cells. It was concluded that EMT is characterized by increased levels of N-cadherin and vimentin, along with a decrease in E-cadherin, which potentiates the invasive properties of breast tumors. Furthermore, Ana Pavlic et al suggest that EMT plays a pivotal role in various aspects of cancer development, including initiation, progression, metastasis, stem cell-like properties, immune evasion, metabolic reprogramming, and the development of treatment resistance.^[7,8]^

Metabolic reprogramming (MR) is a hallmark of cancer cells. Current evidence suggests that the activity of metabolic pathways is altered during tumor development and progression, demonstrating the inherent metabolic plasticity of cancer cells.^[9]^ Xiang et al^[10]^ advocated that abnormal intracellular metabolism not only provides the necessary resources for tumor growth but also impacts the function of various immune cells within the tumor microenvironment, ultimately promoting tumor immune evasion. They^[10]^ specifically described how key metabolic processes, primarily those involving glucose and lipid metabolism, influence both anti-tumor immune cells and immunosuppressive cells (such as macrophages, dendritic cells, T cells, neutrophils, and B cells) within the context of pancreatic cancer progression.

EMT and MR are closely intertwined processes, with metabolic changes often accompanying the EMT process. A meta-analysis of 184 publicly available transcriptome datasets by Muralidharan et al^[11]^ revealed a positive correlation between enhanced expression of PD-L1 (and other immune checkpoint markers) and the intensification of both EMT and glycolysis (a metabolic reprogramming process). This pattern was further observed in single-cell RNA-seq data and time-course EMT induction studies across various cell lines. Additionally, Luo et al^[12]^ demonstrated that 27-hydroxycholesterol (27-HC), a key metabolite in the MR process, promotes EMT by activating the liver X receptor, ultimately contributing to cancer development and metastasis. These studies overlap in their assertion of the significant interaction between EMT and MR, demonstrating their combined influence on tumor metastasis, immune evasion, and other cancer-related processes.

Herein, we comprehensively investigated the crosstalk between EMT and metabolic reprogramming. We developed a Metastasis Score to assess the risk of distant metastasis/recurrence in patients, facilitating risk stratification and clinical management of breast cancer. Additionally, an in-depth analysis of MR and EMT interactions was conducted to identify key genes involved in distant metastasis/recurrence, potentially providing therapeutic targets for patients with intermediate and late-stage breast cancer.

## 2. Materials and methods

### 2.1. Data acquisition

We obtained gene expression data and related clinical information for TCGA-BRCA from The Cancer Genome Atlas Program (TCGA, https://portal.gdc.cancer.gov/). EMT and metabolic reprogramming-related genes were downloaded from GeneCards (https://www.genecards.org/), filtering for scores of 5.50 or higher (metabolic reprogramming) and 6.20 or higher (EMT) to manage the number of genes included. Gene mutation data was acquired from the Genomic Data Commons (GDC; https://portal.gdc.cancer.gov/) and analyzed using the “maftools” package. We investigated the Gene Expression Omnibus (GEO) database and acquired several transcriptome datasets: GSE2034 (microarray data from 286 breast cancer patients with 5-year distant metastasis or recurrence records), GSE17705 (microarray data from 298 patients undergoing tamoxifen endocrine therapy, including distant metastasis and recurrence information), and GSE43837 (gene expression data from both brain metastasis tissue and non-metastatic tumors in 19 HER2-positive patients). Additionally, we obtained the GSE176078 dataset to obtain single-cell RNA sequencing (scRNA-seq) data for breast cancer.

### 2.2. A computational model for the level of MR and EMT in breast cancer

Inspired by the work of Jiahui Zhou et al^[5]^ on the Apoptosis Index and EMT Index for colon adenocarcinoma prognosis, we developed the EMT Potential Index (EPI) and MR Potential Index (MPI). In this regard, we first obtained EMT and MR genes from GeneCards. Using the GSE2034 dataset, we performed univariate Cox analysis with distant metastasis/recurrence time as the target variable. This allowed us to identify significant MR and EMT genes associated with distant metastasis/recurrence. Next, we extracted differentially expressed genes between tumor and adjacent tissues from the TCGA-BRCA dataset. By intersecting these with the significant gene sets from the univariate Cox analysis, we obtained the final MR-related genes (MRG) and EMT-related genes (ERG). We conducted Principal Component Analysis (PCA) on the gene expression matrices of MRG and ERG separately, selecting the first 3 principal components as the main dimensions. Following established methods,^[13]^ we defined EPI and MPI as follows: EPI or MPI = ∑ (PC1i + PC2i + PC3i), where i represents the expression matrix of either ERG or MRG. We then used EPI and MPI to classify patients in the GSE2034 dataset into 4 molecular subtypes: EPI_high + MPI_high; EPI_high + MPI_low; EPI_low + MPI_high; and EPI_low + MPI_low. Kaplan–Meier analysis was used to assess survival differences among these subtypes. To identify differentially expressed genes (DEGs) associated with the highest risk, we compared the (EPI_high + MPI_high) group to the others. Functional enrichment analysis was performed on the DEGs, with criteria for differential expression set as an absolute log fold change (logFC) greater than 1 and a *P*-value less than 0.05. We utilized both the Gene Ontology and Kyoto Encyclopedia of Genes and Genomes datasets and the “clusterProfiler” package for enrichment analysis, with both *P* value and q value cutoffs set to .05.

### 2.3. The crosstalk between MR-related genes and EMT-related genes and the development of Metastasis Score

We performed clustering analysis on the GSE17705 and TCGA-BRCA datasets using the merged expression matrix of MRG and ERG. We next sought to investigate the association between clustering categories and relapse-free survival or disease-free survival. To predict distant metastasis/recurrence time, we employed Least Absolute Selection and Shrinkage Operator (LASSO) regression analysis on the combined MRG and ERG expression matrix, using the GSE2034 dataset for model construction. Based on the regression model’s results, we developed the Metastasis Score. Samples were then classified into high and low score groups using the median Metastasis Score as the threshold. We validated the Metastasis Score on the GSE48837, GSE17705, and TCGA-BRCA datasets. Additionally, using the TCGA-BRCA dataset, we constructed a nomogram incorporating the Metastasis Score and other relevant clinical factors. The nomogram’s predictive ability for 1, 3, and 5-year survival was then evaluated. Finally, we used the TCGA-BRCA dataset to explore the Metastasis Score in relation to various factors, including the tumor microenvironment, immune markers, and genetic variations.

### 2.4. Tumor immune-related analysis

Use the R package “estimate” to calculate the Immune Score and Stromal Score for each patient. To assess the infiltration of various immune cells in the high and low Metastasis Score groups, we employed the R packages “xCell” and ‘MCPcounter’, utilizing the pipelines provided within these packages. For evaluating functional gene expression signatures (Fges) representing cells and functional properties of the tumor microenvironment, we used the website http://science.bostongene.com/tumor-portrait/, as proposed by Bagaev et al^[14]^ It has been established that microsatellite instability (MSI) can be a predictor of immunotherapy response. In the present study, we calculated the MSI for each sample using the R package ‘PreMSIm’,^[15]^ classifying samples as either MSI-high (MSI-H) or MSI-low/microsatellite stable (MSS). Finally, we used the Tumor Immune Dysfunction and Exclusion (TIDE, http://tide.dfci.harvard.edu/login/) platform to obtain individual Exclusion Score, Myeloid-derived Suppressor Cells (MDSCs) Score, Tumor-associated M2 Macrophages (TAM.M2) Score, and Cancer-associated Fibroblasts (CAFs) Score.

### 2.5. Genes involved in Metastasis Score

Utilizing the TCGA-BRCA dataset, we investigated the differential expression of Metastasis Score-related genes between tumor and adjacent tissues. We further explored the potential causes of this differential expression by analyzing genetic mutations, copy number variations (CNVs), and methylation patterns. Additionally, we examined the correlation between Metastasis Score-related genes and genes associated with tumor immunity. This analysis focused on various aspects such as chemokines, immune inhibitors, MHC, immune stimulation, and receptor-related genes.

### 2.6. Single-cell analysis

We utilized the single-cell data from GSE176078 processed by Wu et al,^[16]^ which had undergone quality control and cell annotation by the authors. We employed the UMAP method to visualize the clustering of single cells within the dataset. Next, we extracted the expression matrix for each cell and compared the Metastasis Scores of different cell types. To explore changes in Metastasis Score along a pseudotime trajectory, we used the R package “Monocle 2” to generate the pseudotime trajectory specifically for cancer-associated fibroblast.

### 2.7. Specimen collection

We selected breast cancer patients treated at the Breast Surgery Department of Guangdong Medical University Affiliated Hospital (June 2021 to June 2022) as research subjects. Inclusion criteria were: Patients were diagnosed by pathological examination, complete clinical data, age ≥ 18 years, surgical treatment in the Breast Surgery Department of Guangdong Medical University Affiliated Hospital with a follow-up post-radical tumor resection exceeding 1 year, and absence of concurrent malignant tumors. Patients with imaging (CT, PET-CT, ultrasound, etc.) or pathological evidence of distant organ metastasis/recurrence (e.g., lung, bone, liver) within 1 year of follow-up were classified into the “transfer group”; the remaining patients were categorized as the ‘untransferred group.” We randomly selected 5 cases from the transfer group and obtained breast cancer specimens from the specimen library. Using propensity score matching (factors: gender, age, BMI, tumor stage, etc.), we identified 5 matched cases from the untransferred group and obtained corresponding breast cancer specimens for further study. SPSS 21.0 software facilitated the propensity score matching. This study was approved by the Ethics Committee of Guangdong Medical University Affiliated Hospital and was conducted in accordance with the Helsinki Declaration of the World Medical Association.

### 2.8. Immunohistochemistry

We collected a total of 10 breast cancer tissue samples, equally divided between the transfer group (5 cases) and the matched untransferred group (5 cases). Immunohistochemistry analysis was performed using the streptavidin-peroxidase method (Zymed Laboratories Inc., San Francisco). The HPRT1 antibody (K113171P; Beijing Solarbio Science & Technology Co., Ltd, Beijing, P.R.China) was diluted to 1:50 and applied for 15 seconds to both sample groups to ensure consistent staining time. We determined the Immunohistochemistry score of HPRT1 based on the average optical density (AOD) value, reflecting the staining intensity within tumor cells. ImageJ software was used to calculate the AOD value using the formula: AOD = IOD/Area. A higher AOD value indicates higher HPRT1 expression. Two independent observers evaluated and examined the immunostaining results.

### 2.9. Western blot assay

The target protein was detected using a 15% polyacrylamide gel. After SDS-PAGE electrophoresis, the target protein was transferred to a PVDF membrane (Merck Millipore ISEQ00010). The membrane was blocked with 5% skim milk and then incubated with anti-HPRT1 primary antibody overnight at 4°C. Following two 7-minute washes with TBST, the membrane was incubated with the corresponding diluted HRP-labeled secondary antibody for 1 to 2 hours at room temperature. After 3 additional 7-minute TBST washes, chemiluminescence detection was performed using the White Shark Easy BL520A kit. ImageJ software was used to analyze the target band’s density value. Protein expression differences between the transferred and untransferred groups were compared using GAPDH as an internal reference.

### 2.10. Statistical analysis

The analyses employed included the Wilcoxon test for comparisons of continuous variables, the chi-square test for inter-group comparisons of categorical variables, and the Log-Rank test for inter-group analysis of survival rate. Correlation analysis was performed using either the Pearson or Spearman correlation coefficient, depending on the data characteristics. A *P* value less than .05 was statistically significant, with asterisks denoting significance levels (*P < .05; **P < .01; ***P < .001; ****P < .0001).

## 3. Results

### 3.1. Identification of MRG and ERG related to distant metastasis or recurrence

**Figure 1.**
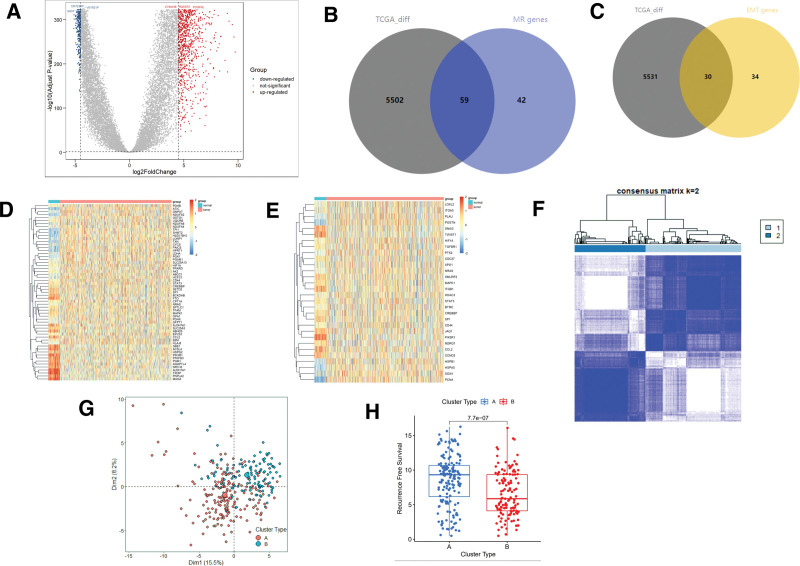
(A) Volcano plot of differentially expressed genes between tumor and adjacent normal tissues in the TCGA-BRCA dataset. (B) Venn diagram of MR-related genes and differentially expressed genes. (C) Venn diagram of EMT-related genes and differentially expressed genes. (D) Heatmap of MRGs expression in tumor and adjacent normal tissues in the TCGA-BRCA dataset. (E) Heatmap of ERGs expression in tumor and adjacent normal tissues in the TCGA-BRCA dataset. (F) Consensus clustering plot of the GSE17705 dataset. (G) PCA distribution plot of different clusters in the GSE17705 dataset. (H) The box plot showing the comparison of the recurrence-free survival between different clusters in GSE17705 dataset. BRCA = breast cancer, EMT = epithelial-mesenchymal transition, ERG = EMT-related genes, MR = metabolic reprogramming, MRG = MR-related genes, PCA = Component Analysis, TCGA = The Cancer Genome Atlas.

A total of 6927 EMT genes and 5433 MR genes were downloaded from GeneCards (Table S1, Supplemental Digital Content, http://links.lww.com/MD/M868). The GSE2034 dataset was used to identify MR and EMT genes associated with distant metastasis or recurrence through univariate Cox analysis, with distant metastasis/recurrence time as the target variable. This analysis identified 101 significant MR genes (Table S2, Supplemental Digital Content, http://links.lww.com/MD/M869) and 64 significant EMT genes (Table S3, Supplemental Digital Content, http://links.lww.com/MD/M870) (*P* < .05). These genes were then intersected with differentially expressed genes between tumor and adjacent tissues in TCGA-BRCA (Fig. 1A), resulting in the identification of MRG (n = 59 genes) and ERG (n = 30 genes) (Fig. 1B and C). Heatmaps were generated to visualize the expression of MRG and ERG in tumor and adjacent tissues from the TCGA-BRCA dataset (Fig. 1D and E). Subsequently, MRG and ERG were merged to create a combined gene set, which was then used for consensus clustering on the GSE17705 dataset (Fig. 1F). Based on the results presented in Figure S1, Supplemental Digital Content, http://links.lww.com/MD/M872, the optimal clustering number k was determined to be 2. PCA was performed to visualize the separation between different clusters (Fig. 1G). The analysis revealed differences in distant metastasis/recurrence time between the clusters (Fig. 1H), suggesting that the combined MRG and ERG gene set could distinguish the distant metastasis status of breast cancer patients.

### 3.2. Construction of MPI and EPI

**Figure 2.**
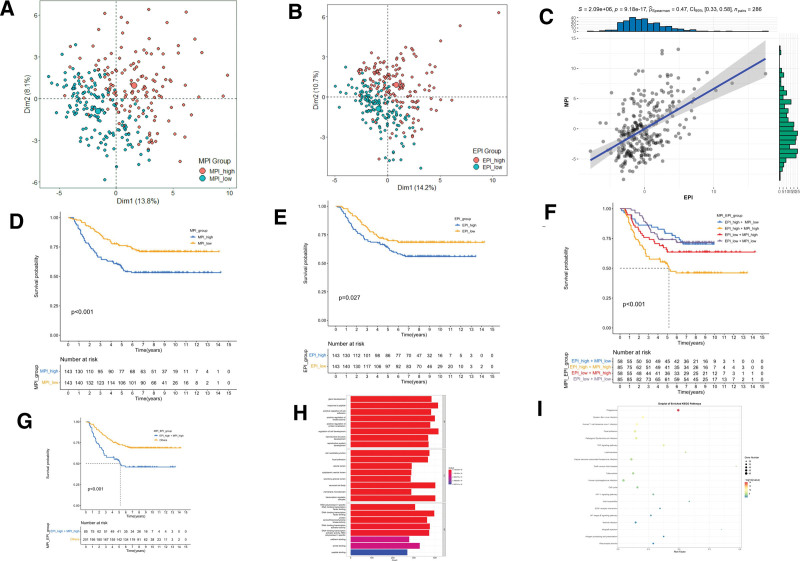
(A) PCA distribution plot of MPI in GSE2034 dataset. (B) PCA distribution plot of EPI in GSE2034 dataset. (C) Scatter plot of EPI and MPI in GSE2034 dataset. (D) Kaplan–Meier analysis of MPI_high and MPI_low group in GSE2034 dataset. (E) Kaplan–Meier analysis of EPI_high and EPI_low group in GSE2034 dataset. (F) Kaplan–Meier analysis of 4 subtypes about MPI and EPI in GSE2034 dataset. (G) Kaplan–Meier analysis of MPI_high + EPI_high subtype and other subtypes in GSE2034 dataset. (H) Box plot showing GO analysis results of the differential genes between MPI_high + EPI_high subtype and other subtypes in GSE2034 dataset. (I) Dot plot showing KEGG analysis results of the differential genes between MPI_high + EPI_high subtype and other subtypes in GSE2034 dataset. EPI = EMT Potential Index, GO = Gene Ontology, KEGG = Kyoto Encyclopedia of Genes and Genomes, MPI = MR Potential Index, PCA = principal component analysis.

To further study the crosstalk between ERG and MRG, EMT and MR levels in each tumor tissue of the GSE2034 dataset were quantified based on PCA. EPI and MPI were then defined separately (Fig. 2A and B). A scatter plot depicting the relationship between EPI and MPI in the GSE2034 dataset was generated (Fig. 2C). Survival analysis results revealed that the MPI_high group had a significantly lower survival rate compared to the MPI_low group (*P* < .0001, Fig. 2D). Similarly, the EPI_high group exhibited a significantly reduced survival rate compared to the EPI_low group (*P* < .05, Fig. 2E), suggesting that both high MPI and high EPI are associated with poor patient outcomes. Subsequently, patients from the GSE2034 dataset were categorized into 4 subtypes based on their EPI and MPI levels: EPI_high + MPI_high, EPI_high + MPI_low, EPI_low + MPI_high, and EPI_low + MPI_low. K-M analysis of the survival of the 4 subtypes (Fig. 2F) demonstrated that the survival rates of the other 3 subtypes were significantly higher than that of the EPI_high + MPI_high group (*P* < .001). The remaining 3 subtypes were combined and designated as “Other” before being compared to the EPI_high + MPI_high subtype. This comparison yielded consistent findings (Fig. 2G), further supporting the notion of an interaction between MPI and EPI, and their combined evaluation can effectively distinguish the prognosis of breast cancer patients with distant metastasis. Differential expression analysis was performed on genes between the EPI_high + MPI_high subtype and the “Other” subtype. Gene Ontology analysis revealed that the differentially expressed genes between the 2 subtypes were enriched in biological processes such as positive regulation of cell adhesion, cell-substrate junction, focal adhesion, and regulation of cell development (as shown in Fig. 2H). The Kyoto Encyclopedia of Genes and Genomes analysis results yielded similar conclusions (as shown in Fig. 2I).

### 3.3. The development of the Metastasis Score

**Figure 3.**
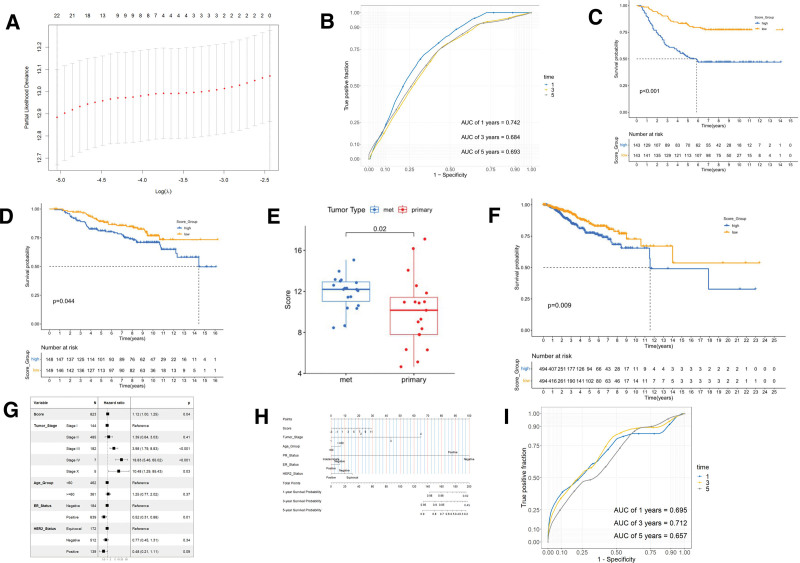
(A) The determination of the lambda value of LASSO regression in GSE2034 dataset. (B) ROC curve of Metastasis Score for predicting 1, 3, and 5-year survival rates in GSE2034 dataset. (C) Kaplan–Meier analysis of the high- and low-Metastasis Score group in GSE2034 dataset. (D) Kaplan–Meier analysis of the high- and low-Metastasis Score group in GSE17705 dataset. (E) Box plot showing the comparison of Metastasis Score between the metastatic and the non-metastatic tissues in GSE48837 dataset. (F) Kaplan–Meier analysis of the high- and low-Metastasis Score group in TCGA-BRCA dataset. (G) Forest plot showing the multivariate Cox regression results on disease-free survival in TCGA-BRCA dataset. (H) Nomogram of the Cox regression in TCGA-BRCA dataset. (I) ROC curve of the nomogram for predicting 1, 3, and 5-year survival in TCGA-BRCA dataset. BRCA = breast cancer, LASSO = Least Absolute Selection and Shrinkage Operator, ROC = receiver operating characteristic, TCGA = The Cancer Genome Atlas.

We then used the combined set of 80 genes from MRG and ERG as independent variables to construct a LASSO regression model for predicting distant metastasis/recurrence time based on the GSE2034 dataset. The relationship between the lambda value of LASSO regression and binomial deviance is shown in Figure 3A. Based on the trade-off between model complexity and performance, a metastasis risk scoring system consisting of 13 genes was ultimately constructed and named Metastasis Score. The calculation formula for Metastasis Score is provided in Table S4, Supplemental Digital Content, http://links.lww.com/MD/M871, with the weight being 10 times the corresponding LASSO regression coefficient. We further assessed the performance of Metastasis Score in predicting distant metastasis time using the GSE2034 dataset. The areas under the receiver operating characteristic curves (AUC) for 1, 3, and 5 years were 0.742, 0.684, and 0.693, respectively (Fig. 3B). Using the median Metastasis Score as the cutoff, patients in different cohorts were separated into the high and low groups. Figure 3C presents the K-M analysis results, demonstrating that the low-score group experienced significantly longer distant metastasis/recurrence time compared to the high-score group.

The GSE17705 dataset contains microarray data for 298 breast cancer patients along with corresponding information on distant metastasis and recurrence. Therefore, we chose to validate the Metastasis Score in both the GSE17705 and TCGA-BRCA datasets. The results for both datasets indicated that a higher Metastasis Score was associated with a poorer prognosis (Fig. 3D–F). In the GSE43837 dataset, containing 19 cases of brain metastasis tissue and 19 non-metastatic breast cancer samples, the comparison boxplot of the Metastasis Score (Fig. 3E) revealed significant differences (*P* < .05), suggesting that the Metastasis Score could, to some extent, differentiate non-metastatic and metastatic breast cancer. Furthermore, based on the TCGA-BRCA dataset, we employed multivariate Cox regression analysis to determine if the Metastasis Score was an independent prognostic factor for disease-free survival, independent of other clinical factors (Fig. 3G). We also constructed a nomogram incorporating the Metastasis Score and other clinical factors (Fig. 3H) and generated ROC curves. The AUCs for 1, 3, and 5 years of disease-free survival were 0.695, 0.712, and 0.657, respectively (Fig. 3I), indicating that the constructed Metastasis Score could reliably reflect the risk of metastasis and prognosis in breast cancer patients.

### 3.4. The immune landscape of high- and low-Metastasis Score group

**Figure 4.**
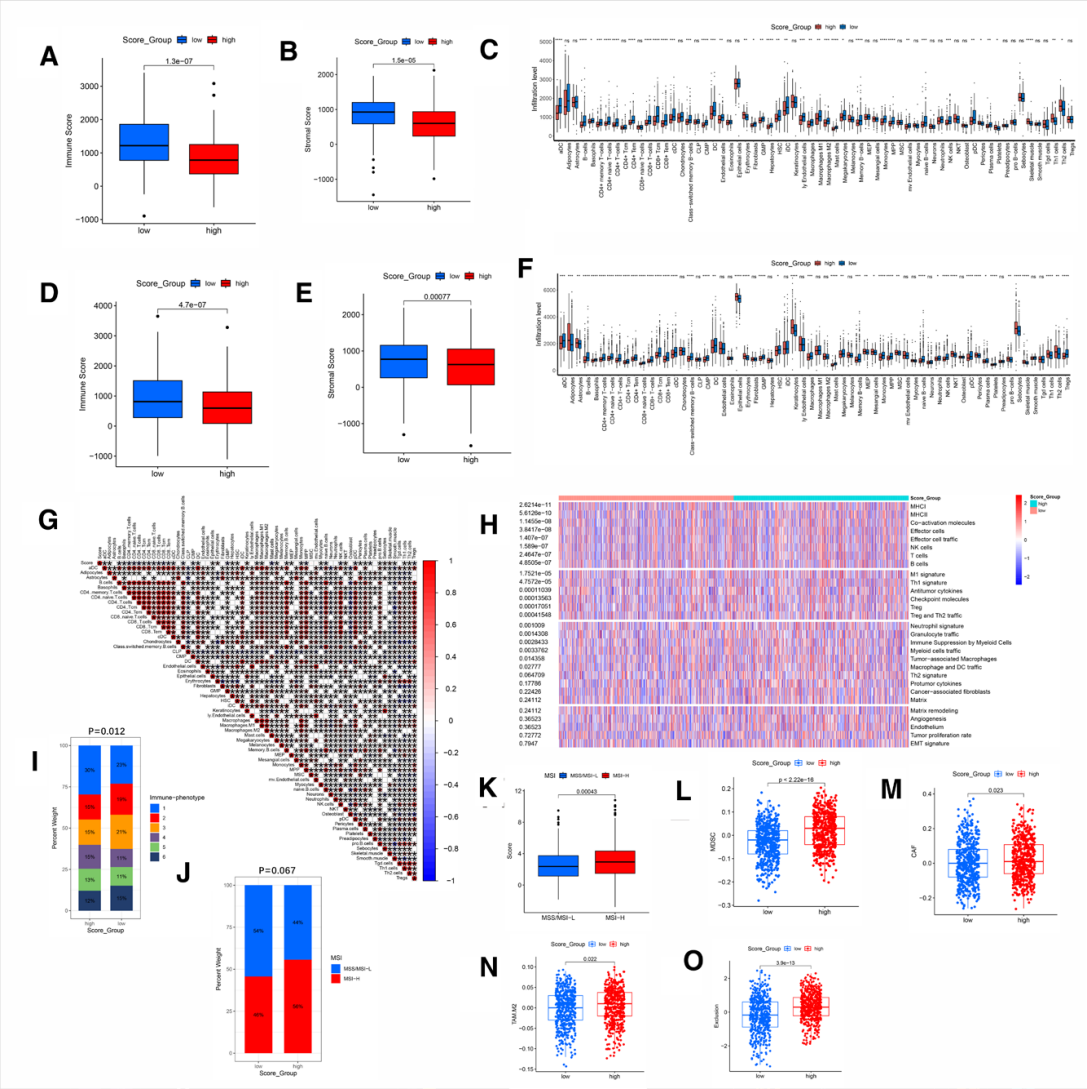
(A) Box plot comparing Immune Score between the high- and low-Metastasis Score group in GSE48837 dataset. Immune Score is estimated by the ESTIMATE algorithm. (B) Box plot showing the comparison of Stromal Score between the high- and low-score group in GSE48837 dataset. Stromal Score is estimated by the ESTIMATE algorithm. (C) Box plot showing the comparison of immune cell infiltration between two groups in GSE48837 dataset. The immune cell infiltration is estimated by the XCELL algorithm. (D) Box plot showing the comparison of Immune Score, estimated by the ESTIMATE algorithm, between the high- and low-Metastasis Score group in TCGA-BRCA dataset. (E) Box plot showing comparison of Stromal Score, estimated by the ESTIMATE algorithm, between the high- and low-score group in TCGA-BRCA dataset. (F) Box plot showing comparison of immune cell infiltration, estimated by the XCELL algorithm, between the two groups in TCGA-BRCA dataset. (G) Correlation coefficient plot of Metastasis Score with various immune cells in TCGA-BRCA dataset. (H) Heatmap showing the comparison of 29 Fregs between two groups in TCGA-BRCA dataset. (I) Comparison of 6 immune subtypes between the high- and low-score group in TCGA-BRCA dataset. (J) Comparison of MSI between the high- and low-score group in TCGA-BRCA dataset. (K) Comparison of Metastasis Score between different MSI subtypes in TCGA-BRCA dataset. (L) Comparison of MDSCs between the high- and low-Metastasis Score group in TCGA-BRCA dataset. (M) Comparison of CAFs between the two groups of Metastasis Score in TCGA-BRCA dataset. (N) Comparison of TAM.M2 between the high and low group of Metastasis Score in TCGA-BRCA dataset. (O) Comparison of Exclusion Score between the two groups of Metastasis Score in TCGA-BRCA dataset. BRCA = breast cancer, CAFs = Cancer-Associated Fibroblasts, MDSCs = myeloid-derived Suppressor Cells, MSI = Microsatellite Instability, TAM.M2 = tumor-associated M2 Macrophages, TCGA = The Cancer Genome Atlas.

The tumor microenvironment is a complex environment consisting of tumor cells, immune cells and stromal cells, which affects the invasion and metabolism of tumor cells.^[17]^ Based on the GSE48837 dataset, ESTIMATE analysis revealed that the low-score group had a significantly higher Immune Score compared to the high-score group (*P* < .001, Fig. 4A). This finding indicates a higher degree of immune cell infiltration in the low-score group. Similarly, the Stromal Score of the low-score group was also significantly higher (*P* < .001, Fig. 4B). Using the XCELL algorithm to evaluate immune cell infiltration, we found that the infiltration of 18 immune cell types, including activated dendritic cells, B cells, CD4 + memory T cells, CD8 + naive T cells, CD4 + naive T cells, CD8 + T cells, CD8 + Tem cells, CD4 + T cells, CD4 + Tem cells, M1 macrophages, and natural killer cells, was also significantly higher in the low-Metastasis Score group (Fig. 4C). Similar results were also observed when using MCPcounter algorithm (Figure S2A and B, Supplemental Digital Content, http://links.lww.com/MD/M873).

We conducted a similar analysis on the TCGA-BRCA dataset and discovered that the Immune Score and Stromal Score were also significantly higher (*P* < .001) in the low-Metastasis Score group (Fig. 4D and E). The infiltration of immune cell types was largely consistent with the results of the GSE48837 dataset (Fig. 4F). Furthermore, we generated a correlation coefficient graph between Metastasis Score and various immune cells in the TCGA-BRCA dataset. We found that the scores of 14 immune cell types, including CD4 + memory T cells, T cells, CD4 + T cells, CD4 + Tcm cells, CD8 naive T cells, CD8 + T cells, CD8 + Tcm cells, and natural killer cells, were negatively correlated with Metastasis Score (Fig. 4G). This finding further supports the observation that higher Metastasis Score is associated with poorer immune cell infiltration.

We compared the scores of 29 Fges between the 2 groups and found that scores for the Th2 signature, Treg, MHC II, Effector cell traffic, T cells, NK cells, Effector cells, Checkpoint molecules, Th1 signature, Co-activation molecules, MHCI, and other anti-tumor immune markers were significantly higher in the low-Metastasis Score group (Fig. 4H). This finding suggests a more favorable immune environment in the low-score group. Some studies have classified breast cancer into 1 to 6 immune phenotypes, where phenotypes 1 and 2 are associated with a poorly cytotoxic immune state, phenotypes 3 and 4 are associated with a moderately cytotoxic immune state, and phenotypes 5 and 6 are associated with a highly cytotoxic immune state. As shown in Figure 4I, the distribution of immune subtypes differed significantly between the high-score and low-score groups (chi-square test, *P* < .05). This suggests that the 2 groups have distinct immune profiles.

The response to immunotherapy is known to be heavily influenced by microsatellite instability. To explore the potential relationship between the Metastasis Score and microsatellite instability, we analyzed the distribution of these factors in the 2 groups. As shown in Figure 4J, the high-Metastasis Score group comprised 44% MSS/MSI-L and 56% MSI-H subtypes, while the low-score group consisted of 54% MSS/MSI-L and 46% MSI-H subtypes. Notably, a statistically significant difference in Metastasis Score was observed between the MSI-H and MSS/MSI-L subtypes (*P* = .0043, Fig. 4K). This finding suggests a potential link between high Metastasis Score and tumor immune suppression in the high-score group.

In addition, we observed that the scores of CAF, TAM.M2, MDSCs and Exclusion in the 2 groups of the TCGA-BRCA dataset were significantly different (*P* < .05, as shown in Fig. 4L–O). The higher the Metastasis Score, the higher the CAF, TAM.M2, MDSC and Exclusion Score, indicating that high Metastasis Score is associated with immune suppression.

### 3.5. Genetic variations

**Figure 5.**
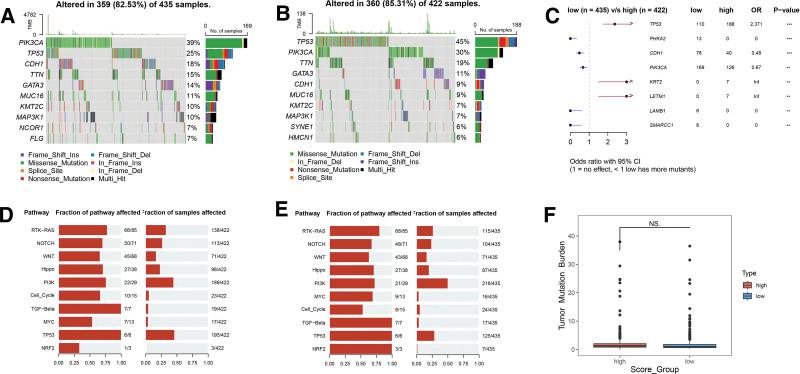
(A) Waterfall plot of the mutated genes of low-Metastasis Score group in TCGA-BRCA dataset. (B) Waterfall plot of the mutated genes of high-Metastasis Score group in TCGA-BRCA dataset. (C) Forest plot showing the comparison results of mutated genes between the high- and low-Metastasis Score group. (D) Significantly enriched pathways of mutated genes of the high-Metastasis Score group in TCGA-BRCA dataset. (E) Significantly enriched pathways of the low-score group in TCGA-BRCA dataset. (F) Box plot comparing tumor mutation burden between the high- and low-Metastasis Score group in TCGA-BRCA dataset. BRCA = breast cancer, TCGA = The Cancer Genome Atlas.

**Figure 6.**
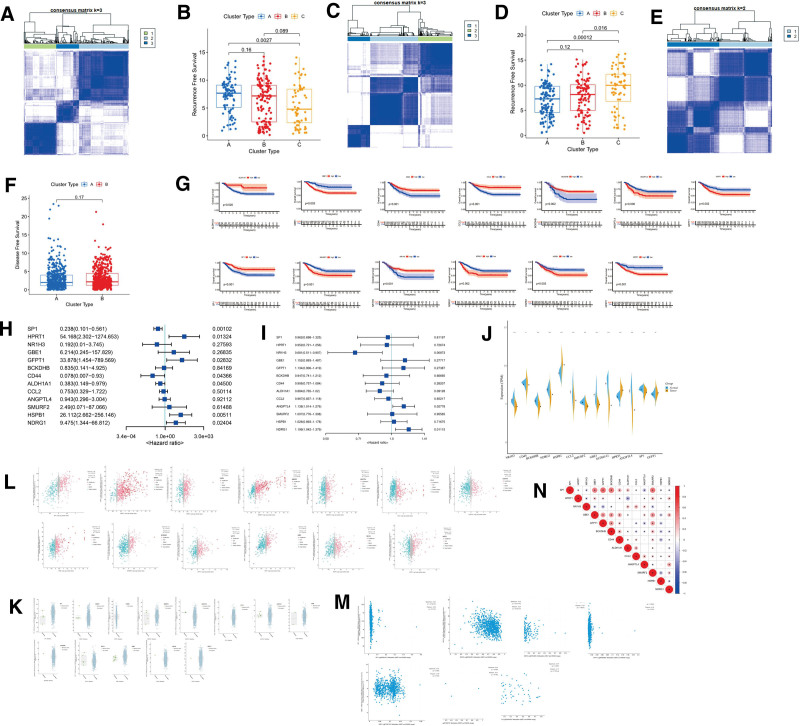
(A) Consensus clustering of the GSE2034 dataset. (B) Box plot comparing distant metastasis/recurrence time among different clusters in GSE2034 dataset. (C) Consensus clustering of the GSE17705 dataset. (D) Box plot comparing distant metastasis/recurrence time among different clusters in GSE17705 dataset. (E) Consensus clustering of the TCGA-BRCA dataset. (F) Box plot comparing disease-free survival between different clusters in TCGA-BRCA dataset. (G) Kaplan–Meier analysis of 13 genes involved in Metastasis Score in GSE2034 dataset. (H) Forest plot showing the result of single-factor Cox analysis of 13 selected genes in GSE17705 dataset. (I) Forest plot showing the result of single-factor Cox analysis of 13 selected genes in TCGA-BRCA dataset. (J) Box plot comparing the expression of 13 selected genes between adjacent and cancer tissue in TCGA-BRCA dataset. (K) Correlation coefficient graph showing the relation between gene expression and gene mutation for each selected gene based on TCGA-BRCA dataset. (L) Correlation coefficient graph with association between gene expression and copy number variation for 13 selected genes in TCGA-BRCA dataset. (M) Correlation coefficient graph with relation between gene expression and methylation for each gene in TCGA-BRCA dataset. (N) Correlation coefficient graph showing the co-expression relationship among 13 selected genes based on TCGA-BRCA dataset. BRCA = breast cancer, TCGA = The Cancer Genome Atlas.

We further investigated the genetic variations, specifically somatic mutations, within the high- and low-Metastasis Score groups using the TCGA-BRCA dataset. Waterfall plots depicting the mutated genes in each group are shown in Figure 5A and B, respectively. The prevalence of somatic mutations was higher in the high-score group (85.31%) compared to the low-score group (82.53%). The top 8 genes with the highest mutation frequency were the same in both groups, namely TP53, PIK3CA, TTN, CDH1, GATA3, MUC16, KMT2C, and MAP3K1. Figure 5C shows the differences in somatic gene mutations between the 2 groups. TP53 (*P* < 1.0 × 10^−3^), KRT2 (*P* < 1.0 × 10^−2^), and LETM1 (*P* < 1.0 × 10^−2^) mutations were significantly higher in the high-score group, while PHKA2 (*P* < 1.0 × 10^−3^), CDH1 (*P* < 1.0 × 10^−3^), PIK3CA (*P* < 1.0 × 10^−2^), LAMB1 (*P* < 1.0 × 10^−2^), and SMARCC1 (*P* < 1.0 × 10^−2^) mutations were higher in the low-Metastasis Score group. While both groups displayed enrichment in similar tumor-related signaling pathways like RTK-RAS, NOTCH, WNT, and Hippo (Fig. 6D and E), the high-score group demonstrated a significantly higher degree of enrichment in pathways associated with cell cycle progression (Cell_Cycle) and TGF-Beta signaling. Additionally, the high-Metastasis Score group exhibited a higher tumor mutation burden compared to the low-score group (Fig. 6F).

### 3.6. 13 selected genes

The 13 genes used in the Metastasis Score were employed for consensus clustering analysis in the GSE2034 dataset. Based on the results in Figure S3A, Supplemental Digital Content, http://links.lww.com/MD/M874, the optimal parameter K was chosen as 3. This value divided the samples into 3 distinct clusters (Fig. 6A). The distant metastasis/recurrence times for these clusters were compared and revealed significant differences (Fig. 6B). The same clustering approach was applied to the GSE17705 data. Similar to the GSE2034 analysis, the optimal K value was determined to be 3 in Figure S3B, Supplemental Digital Content, http://links.lww.com/MD/M874. Again, significant differences in distant metastasis/recurrence time were observed among the 3 identified clusters (Fig. 6C and D). Clustering analysis for the TCGA-BRCA dataset resulted in 2 subgroups when K was set to 2 (Figure S3C, Supplemental Digital Content, http://links.lww.com/MD/M874, Fig. 6E). While there was a difference in disease-free survival between these 2 subgroups (Fig. 6F), the difference did not reach statistical significance. Further analysis using K-M curves in the GSE2034 dataset revealed that all 13 genes were associated with patient distant metastasis/recurrence time (Fig. 6G). Notably, high expression of the HPRT1 gene was linked to a poorer prognosis. The K-M analysis results for the GSE17705 (Figure S3D, Supplemental Digital Content, http://links.lww.com/MD/M874) and TCGA-BRCA datasets (Figure S3E, Supplemental Digital Content, http://links.lww.com/MD/M874) further corroborated the association between the genes included in the Metastasis Score and patient prognosis. Additionally, single-factor Cox analysis of the 13 genes in the GSE17705 dataset identified SP1, CD44, and ALDH1A1 as favorable prognostic factors, while HPRT1 and NDRG1 emerged as unfavorable factors (Fig. 6H). The single-factor Cox analysis results for the TCGA-BRCA dataset are presented in Figure 6I.

We investigated the differential gene expression between breast cancer tumors and normal tissues in the TCGA-BRCA dataset. Three genes, *HSPB1, HPRT1*, and *GFPT1*, were found to be significantly upregulated in tumor samples compared to normal samples. Conversely, the remaining 10 genes exhibited the opposite trend, with higher expression observed in normal tissues (Fig. 6J). To explore the potential factors driving these gene expression changes, we utilized the cBioPortal database to analyze the correlations between the expression levels of the 13 genes and various genomic alterations, including gene mutations (Fig. 6K), CNVs (Fig. 6L), and methylation (Fig. 6M). Furthermore, the correlation analysis of the 13 gene expression levels revealed a strong positive correlation between *HPRT1, SMURF2*, and *NDRG1* (Fig. 6N).

### 3.7. Each selected gene and immune

**Figure 7.**
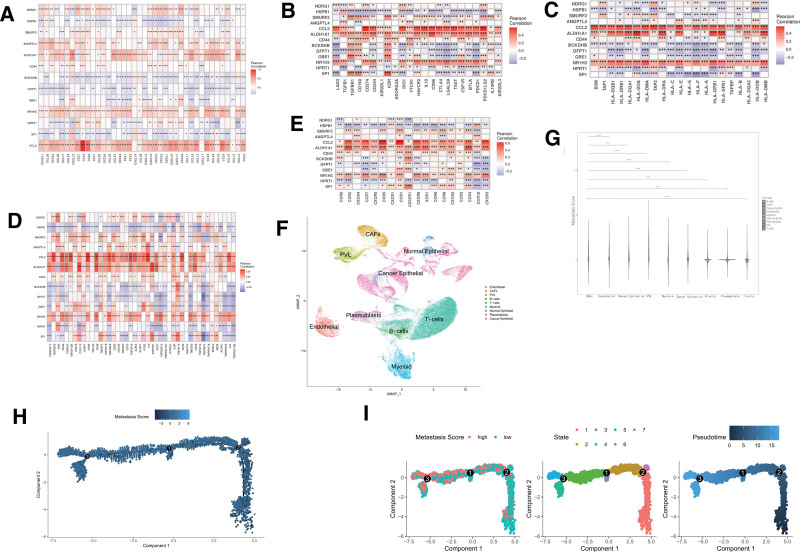
(A) Correlation coefficient graph showing the expression relationship between 13 selected genes and chemokine-related genes. (B) Correlation coefficient graph showing the expression relationship between 13 selected genes and immune-inhibitor genes based on TCGA-BRCA dataset. (C) Correlation coefficient graph with relationship between 13 selected genes and MHC-related genes. (D) Correlation coefficient graph with relationship between 13 genes and immune stimulation-related genes. (E) Correlation coefficient graph showing the relationship between 13 selected genes and receptor-related genes. (F) tSNE plot of 9 cell types in single-cell analysis. All cells are divided into 9 cell types: Cancer Associated Fibroblasts (CAFs), Myeloid, Plasma Cells, Endothelial, Perivascular-like Cells, Normal Epithelial, B-cells, T-cells, and Cancer Epithelial. (G) Comparison of Metastasis Score among 9 cell types. (H) Pseudo-time analysis result of CAFs. (I) Pseudo-time analysis result about Metastasis Score of CAFs. BRCA = breast cancer, tSNE = t-distributed stochastic neighbor embedding, TCGA = The Cancer Genome Atlas.

In this study, we explored the relationship between the expression levels of the 13 selected genes and the tumor microenvironment. This was achieved by analyzing their correlation with the expression of genes associated with various immune functions, including chemokines, immune inhibitors, MHC molecules, immune stimulation, and receptors. The correlation results presented in Figure 7A–E demonstrate that these 13 genes exhibit significant relationships with these 5 types of immune genes. Among the 13 genes, *HPRT1* stood out as negatively correlated with the expression of most chemokines, immune inhibitors, MHC-related genes, immune stimulation-related genes, and receptor-related genes. Conversely, genes such as *CCL2, ALDH1A1*, and *NR1H3* show positive correlations with most immune genes.

### 3.8. Single cell analysis

We analyzed 26 breast cancer single-cell sequencing samples comprising 3 subtypes (ER+, TNBC, and HER2+) and a total of 100,064 cells. All cells were classified into nine distinct cell types: CAFs, Myeloid, Plasma Cells, Endothelial, Perivascular-like Cells, Normal Epithelial, B-cells, T-cells, and Cancer Epithelial (Fig. 7F). Fibroblasts play a crucial role in driving stromal transformation, and the activation of CAFs within the tumor microenvironment is a key event in facilitating epithelial-to-mesenchymal transition (EMT). We calculated the Metastasis Score for each cell type and compared these scores (Fig. 7G). Notably, CAFs exhibited a significantly higher Metastasis Score than all other cell types. We then isolated CAFs for further analysis using the median Metastasis Score as a cutoff to separate them into high-scoring and low-scoring subgroups. To gain insights into CAF functionality, we employed Monocle 2 to determine their developmental trajectory. Pseudotime analysis revealed that CAF cells were distributed across different stages along the time trajectory (Fig. 7H), indicating distinct functional states. Interestingly, these states aligned with the Metastasis Score groupings (Fig. 7I). This finding suggests that the Metastasis Score reflects, to some degree, the functional state of CAFs.

### 3.9. Experimental results of HPRT1

**Figure 8.**
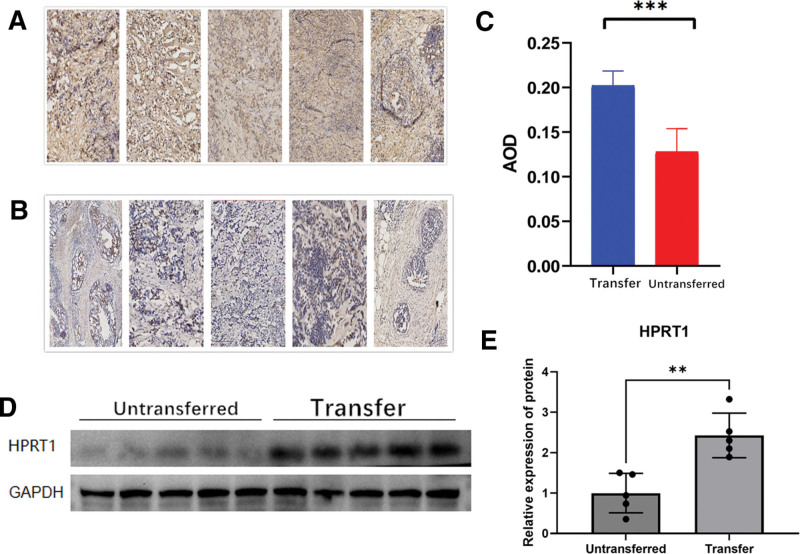
(A) Immunohistochemical results of breast cancer specimens in the transfer group. (B) Immunohistochemical results of breast cancer specimens in the untransferred group. (C) Comparison of AOD of immunohistochemistry of specimens between the transfer group and untransferred group. (D) Western Blot results show the expression of HPRT1 in breast cancer specimens in the transfer group and untransferred group. (E) Comparison of western blot results of HPRT1 expression between the transfer group and the untransferred group. AOD = average optical density.

Based on the results of immunohistochemistry, we found that the HPRT1 staining of breast cancer tissues in 5 cases of the transfer group was significantly deeper (Fig. 8A), while that in the untransferred group was lighter (Fig. 8B). The comparison results of AOD values showed that the HPRT1 expression of BRCA in the untransferred group was significantly lower than that in the transfer group (*P* < 0.001) (Fig. 8C). The Western blot results showed that the protein level of HPRT1 in the transfer group was relatively higher than that in the untransferred group (Fig. 8D), and the two had significant statistical differences (*P* < 0.01) (Fig. 8E). These results further indicate that the expression level of the HPRT1 gene can distinguish the prognosis of BRCA patients to some extent.

## 4. Discussion

Breast cancer is a devastating disease with a rapidly rising incidence rate, now ranking as the second most common malignancy in women after lung cancer.^[18]^ Distant metastasis significantly worsens prognosis in breast cancer patients. Despite advancements in treatment options, BRCA often spreads to vital organs like the lungs, severely impacting survival rates. Additionally, high recurrence rates contribute to mortality in BRCA patients. Therefore, identifying key genes involved in BRCA metastasis and recurrence is paramount for early clinical detection and the development of targeted therapies. Metabolic reprogramming is a hallmark of cancer, with various signaling pathways coordinating this metabolic shift to drive cancer development and progression. Çubuk et al^[19]^ modeled the metabolism and EMT activity of BRCA and found that there is complex crosstalk between the two.^[20,21]^ Recent research demonstrates that not only can metabolic reprogramming promote EMT and increase metastatic potential, but the induction of EMT can also reshape metabolic processes.^[22–26]^ In addition, studies have shown that mtROS are able to drive EMT^[27]^ and control cancer invasion.^[28,29]^ Crucially, mtROS are essential for hypoxia-induced cancer invasiveness,^[29]^ a process intrinsically linked to metabolic reprogramming.^[30]^ These findings collectively suggest a significant crosstalk between EMT and metabolic reprogramming, which jointly influence tumor invasion, metastasis, and other critical processes. Thus, investigating this crosstalk holds the potential of unveiling key genes involved in distant metastasis and recurrence.

In this study, we used PCA algorithm to construct MPI and EPI for quantifying the levels of MR and EMT activity in breast cancer, respectively. By integrating these indices, we classified the samples into 4 distinct subtypes. Notably, the EPI_high + MPI_high subtype exhibited the worst prognosis for distant metastasis/recurrence, highlighting the deleterious influence of co-occurring high MR and EMT activity. This finding aligns with previous research demonstrating the association between EMT and the acquisition of metastatic potential and drug resistance in cancer cells.^[31]^ During metastasis, cancer cells adapt their metabolic profiles to survive in diverse environments, often switching between different metabolic pathways.^[32–34]^ This metabolic flexibility enhances their invasive ability and survival, ultimately impacting patient treatment outcomes. Collectively, our results suggest that both high MR and EMT levels significantly worsen patient prognosis in breast cancer, and their combined assessment through MPI and EPI offers a more robust approach to predicting distant metastasis/recurrence.

We developed the Metastasis Score using the LASSO model to assess the risk of distant metastasis and recurrence in breast cancer patients, integrating both MR and EMT levels. The score demonstrated superior predictive performance in the GSE2034 and GSE17705 datasets (Fig. 3B and D), showcasing its ability to reflect BRCA metastasis and recurrence. Additionally, Kaplan–Meier analysis (Fig. 3F) and Cox multivariate regression analysis (Fig. 3G) in the TCGA-BRCA dataset confirmed the Metastasis Score’s strong association with disease-free survival in BRCA patients. Furthermore, its ability to differentiate between primary and metastatic breast cancer within the GSE43837 dataset further validates its discriminatory power. In conclusion, the constructed Metastasis Score effectively integrates MR and EMT levels in breast cancer, offering a powerful tool for predicting metastasis and prognosis in BRCA patients.

Given the close link between the tumor microenvironment and tumor metastasis, we further investigated the relationship between the Metastasis Score and tumor immunity. Analysis of immune cell infiltration revealed lower abundances of CD8+ naive T cells, CD8+ T cells, CD8+ Tem cells, CD4+ naive T cells, and CD4+ T cells in patients with high Metastasis Score (Fig. 4F). Consistent with this finding, correlation analysis demonstrated a negative correlation between the Metastasis Score and the infiltration of 14 immune cell types known to be positively correlated with anti-tumor immunity, including CD4+ memory T cells, CD4+ T cells, CD4+ Tcm cells, CD8 naive T cells, CD8+ T cells, CD8+ Tcm cells, and natural killer cells (Fig. 4G).^[35]^ Furthermore, analyses of 29 Fges (Fig. 4H), immune subtype composition ratio (Fig. 4I), CAF Score (Fig. 4L), TAM.M2 Score (Fig. 4M), MDSCs Score (Fig. 4N), and Exclusion Score (Fig. 4O) all pointed towards a weaker anti-tumor immune response in the high-Metastasis Score group compared to the low-score group. Single-cell analysis revealed a higher Metastasis Score in breast cancer-associated fibroblasts compared to other cell types (Fig. 7G). Studies have shown that these CAFs can induce glycolysis, which in turn facilitates the expression of EMT-related genes in breast cancer cells, ultimately promoting metastasis.^[36]^ Our Monocle 2 analysis (Fig. 7H and I) further supports the notion that the Metastasis Score reflects distinct functional states within CAF cells. In conclusion, the Metastasis Score not only reflects the metastatic potential of breast cancer but also exhibits a close relationship with CAFs. These findings suggest that CAFs play a key role in the crosstalk between metabolic reprogramming and EMT in breast cancer progression.

The Metastasis Score incorporates 13 genes, all of which are implicated in the complex interplay between metabolic reprogramming and EMT in breast cancer, warranting further investigation. Specifically, the score includes genes associated with EMT (SP1, CD44, CCL2, SMURF2, HSPB1, and NDRG1) and genes associated with MR (SP1, HPRT1, NR1H3, GBE1, GFPT1, BCKDHB, ALDH1A1, and ANGPTL4). Notably, SP1, CD44, and CCL2 exhibited involvement in both EMT and MR processes.

The SP1 gene encodes a zinc finger transcription factor crucial for various cellular processes, including cell growth and immune response.^[37]^ Upregulation of SP1 activity can induce EMT^[38]^ and contributes to drug resistance through metabolic reprogramming. Specifically, SP1 regulates prostaglandin E2 production, which activates the FAO and TCA cycles in mitochondria via EP1 and EP3 receptors. This activation ultimately leads to temozolomide resistance in glioblastoma.^[39]^ CD44, a known cancer stem cell marker in various cancers,^[40]^ and CCL2, a secreted protein, play pivotal roles in breast cancer metastasis and recurrence.^[41]^ These 2 genes are key players in metastasis and recurrence of breast cancer. Glycogen branching enzyme (GBE1) is a key gene involved in regulating glycogen metabolism and mediating metabolic reprogramming of cells.^[42]^ NR1H3, a nuclear receptor, functions as a key regulator of both energy and lipid homeostasis.^[43]^ Glutamine-fructose-6-phosphate transaminase 1 (GFPT1) encodes the rate-limiting enzyme of the hexosamine pathway, controlling glucose flux into this metabolic pathway.^[44]^ BCKDHB, encoding part of a multi-enzyme complex associated with the mitochondrial inner membrane, is involved in the catabolism of branched-chain amino acids. ALDH1A1, belonging to the aldehyde dehydrogenase family, plays a crucial role in alcohol metabolism,^[45]^ while Angiopoietin-like 4 (ANGPTL4) gene encodes a glycosylated secreted protein that participates in regulating glucose homeostasis and lipid metabolism.^[46]^ Hypoxanthine-guanine phosphoribosyltransferase 1 (HPRT1) is involved in purine metabolism, and can positively regulate genes associated with cancer-related pathways.^[47]^

SMURF2, a member of the HECT E3 ubiquitin ligase family, plays a significant role in the EMT process. Studies have indicated that silencing SMURF2 can reduce the proliferation of breast cancer cells.^[48]^ Heat shock protein β-1 (HSPB1) gene encodes a member of the small heat shock protein family, being an important biological marker of various cancer pathologies. HSPB1 also participates in epithelial-mesenchymal transition, thereby affecting breast cancer metastasis.^[49]^ NDRG1 is a member of the N-myc downregulated gene family. The protein encoded by this gene participates in stress response, cell growth, and differentiation. Research demonstrates a negative correlation between NDRG1 expression and breast cancer metastasis and progression, suggesting its potential as a prognostic biomarker for early metastasis prediction.^[50]^

Our analysis demonstrated that patients in the GSE2034 and GSE17705 datasets could be stratified into distinct prognostic subgroups based on the genotypes of these 13 genes. This 13-gene set, therefore, holds promise for classifying breast cancer patients and identifying those at higher risk of distant recurrence or metastasis. All 13 genes exhibited differential expression between breast cancer tumors and normal tissues. Furthermore, Kaplan–Meier analysis and single-factor Cox analysis in the GSE2034, GSE17705, and TCGA-BRCA datasets revealed a strong association with breast cancer metastasis and patient prognosis. Additionally, correlation analysis of chemokine, immune inhibitor, MHC-related, immune stimulation-related, and receptor-related gene expression revealed a close relationship between these 13 genes and tumor immunity. Collectively, these findings suggest the potential of these 13 genes as both tumor markers for diagnosis and as therapeutic targets for targeted therapies in breast cancer.

We investigated the potential drivers of differential expression of the 13 genes in BRCA tumors compared to adjacent tissues, considering factors such as mutations, CNVs, and methylation. Interestingly, these factors alone could not fully explain the observed differences in expression. We then focused on HPRT1, traditionally viewed as a housekeeping gene. However, recent studies have demonstrated its upregulation in various cancers and association with clinical outcomes. Subtype-specific analysis confirmed that HPRT1 expression was highest in triple-negative breast cancer.^[40]^ Our study further reinforces the link between HPRT1, breast cancer metastasis, and prognosis, revealing its negative correlation with tumor immunity. Experimental results support the association between HPRT1 and breast cancer metastasis risk. Collectively, these findings offer promising new avenues for the treatment of mid-to-late-stage breast cancer.

This study investigated the combined impact of EMT and metabolic reprogramming on breast cancer metastasis and prognosis. We developed the Metastasis Score, which accurately assesses the risk of distant metastasis/recurrence, enabling risk stratification and improved clinical management for breast cancer patients. We identified key genes involved in the crosstalk between MR and EMT, analyzing them from the perspectives of prognosis and tumor immunity to suggest promising new tumor markers or therapeutic targets. While this study offers valuable insights, it is important to acknowledge the following limitations. Firstly, the Metastasis Score was constructed and validated using TCGA and GEO datasets, which may have inherent biases and require further validation with real-world data. Secondly, the specific roles of the 13 identified genes in driving breast cancer metastasis warrant further experimental investigation. Despite these limitations, our findings hold significant value for understanding the complex interplay between EMT, MR, and breast cancer progression.

## Author contributions

**Conceptualization:** Yingyu Chen, Liyan Yu, Kangwei Luo.

**Data curation:** Liyan Yu, Yongni Chen, Kangwei Luo.

**Formal analysis:** Liyan Yu, Yongni Chen.

**Resources:** Kangwei Luo.

**Software:** Liyan Yu, Kangwei Luo.

**Supervision:** Kangwei Luo.

**Validation:** Yingyu Chen, Yongni Chen.

**Visualization:** Yingyu Chen, Yongni Chen.

**Writing – original draft:** Liyan Yu.

**Writing – review & editing:** Yingyu Chen, Yongni Chen, Kangwei Luo.

## Supplementary Material









**Figure:**
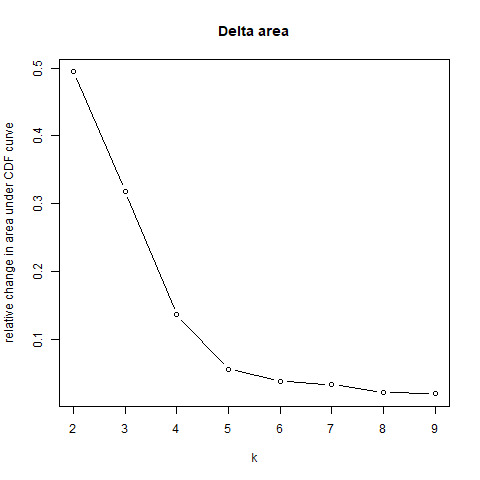


**Figure:**
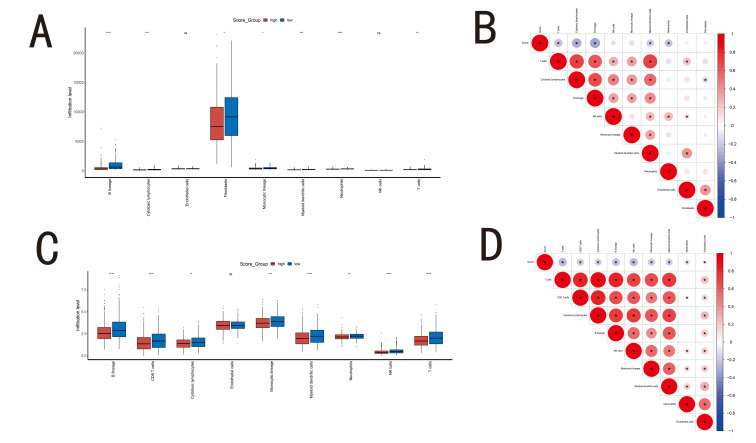


**Figure:**