# Supplementary material for: The crosstalk between metabolic reprogramming and epithelial-mesenchymal transition and their synergistic roles in distant metastasis in breast cancer
Source: Medicine (Baltimore). 2024 Jun 14;103(24):e38462. doi: 10.1097/MD.0000000000038462 (PMC11175907; doi:10.1097/MD.0000000000038462)
Supplement: Supplementary file 1 [file medi-103-e38462-s001.docx]

**Supplementary Table 1 The list of EMT and MR genes**

| **Epithelial-mesenchymal transition** | **Metabolic reprogramming** |
| --- | --- |
| CDH1 | INS |
| TGFB1 | APOE |
| TP53 | LINC01672 |
| CTNNB1 | PPARG |
| AKT1 | LEP |
| SNAI1 | ACADM |
| SNAI2 | MTHFR |
| ZEB1 | LINC-ROR |
| PTEN | MEG3 |
| H19 | MMACHC |
| MEG3 | BDNF-AS |
| TWIST1 | LDLR |
| PVT1 | H19 |
| MALAT1 | LPL |
| MIR7-3HG | MIR122 |
| GAS5 | IL6 |
| ERBB2 | PPARA |
| KRAS | CBS |
| HRAS | PPARGC1A |
| MAPK1 | TP53 |
| EGFR | ALB |
| VIM | SOD2-OT1 |
| RB1 | MIR33A |
| FGFR3 | SLC2A1 |
| PIK3CA | GCK |
| HOTAIR | AKT1 |
| SMAD3 | OTC |
| STAT3 | HADHA |
| XIST | SMAD5-AS1 |
| CCND1 | FH |
| MIR21 | SLC16A1 |
| BRAF | INSR |
| SMAD4 | DYRK1B |
| CDKN2A | IGF1 |
| ZEB2 | CRP |
| HIF1A | ACADVL |
| SMAD2 | UCP2 |
| TUG1 | TNF |
| MTOR | LDHA |
| FBXW7 | HMGCL |
| FGFR1 | GPT |
| HULC | APOA1 |
| CDK1 | HIF1A |
| GSK3B | TMX2-CTNND1 |
| MIR200C | PDHA1 |
| EP300 | MIR7-3HG |
| SCARNA5 | VDR |
| ERBB3 | PC |
| EZH2 | PCCB |
| NOTCH1 | ACADS |
| MIR203A | PPARD |
| TMX2-CTNND1 | CPT1A |
| ZFAS1 | HNF4A |
| CDKN1A | CPT2 |
| CDH2 | MYC |
| CERNA3 | G6PD |
| MIR205 | HSD11B1 |
| LINC-ROR | FASN |
| MIR145 | RETN |
| CD44 | ETFDH |
| HOTTIP | SLC25A13 |
| BRCA1 | LMNA |
| CDKN1B | XIST |
| MMP9 | PKM |
| YAP1 | HSD17B10 |
| MYC | IRS1 |
| MET | CD36 |
| NFE2L2 | HMGCR |
| SPRY4-IT1 | GCG |
| IL6 | IDH2 |
| CYTOR | G6PC1 |
| SRC | SIRT1 |
| MIR200B | CHKB-CPT1B |
| BANCR | SLC2A4 |
| FOXM1 | MIR125A |
| TP63 | PCCA |
| MIR493HG | SREBF1 |
| ENSG00000276919 | ALDH2 |
| MIR200A | MT-TL1 |
| TGFBR1 | FBP1 |
| CASC8 | ASS1 |
| MAP2K1 | ABCA1 |
| CDK2 | IVD |
| LINC01672 | HK2 |
| CREBBP | POR |
| TGFB2 | POMC |
| MIR125A | CYP27B1 |
| NRAS | IL10 |
| PDPN | LINC02605 |
| MIRLET7C | GSTP1 |
| UCA1 | GOT2 |
| MIR34C | MIR34A |
| DANCR | MTHFD1 |
| FN1 | DLD |
| PIK3R1 | ALDOB |
| ZEB2-AS1 | PDHX |
| SNHG16 | MIR21 |
| CCAT1 | PRKAA1 |
| MMP2 | OGDH |
| ATM | IL1B |
| AURKA | SERPINE1 |
| ENSG00000277577 | BCKDHA |
| MIR221 | MIR126 |
| MIR34A | HADHB |
| MIR133B | GAPDH |
| HMGA2 | BCKDHB |
| CCNB1 | ETFA |
| CRNDE | NR3C1 |
| ILK | COMT |
| MIR30A | CPS1 |
| NFKB1 | HPRT1 |
| ENSG00000275307 | CYP21A2 |
| ENSG00000276965 | GLUD1 |
| SIRT1 | PTEN |
| VEGFA | MIR155 |
| BCL2 | CERNA3 |
| MIR141 | EPHX1 |
| S100A4 | NOS3 |
| HNF1A-AS1 | GLS |
| EGF | ABCB1 |
| CCNE1 | PSAP |
| TNF | POU5F1 |
| CHEK2 | ADA |
| CCNA2 | FTO |
| MIR195 | AUH |
| MIR143 | ETFB |
| E2F1 | HADH |
| TGFBR2 | RPIA |
| SNHG5 | MCCC2 |
| CDK4 | PRKAA2 |
| HOXA11-AS | KRAS |
| MIR17 | SLC4A1 |
| BMI1 | CAT |
| BMP4 | MIR17 |
| MIR373 | NFE2L2 |
| SP1 | HIBCH |
| WWTR1 | SFTPC |
| AR | NR1H4 |
| LATS2 | ABCC8 |
| CDK6 | NR1H2 |
| MIR31 | HEXA |
| FAM3C | ACACA |
| CCAT2 | MT-TP |
| GLI1 | CS |
| CASC19 | GLDC |
| PCAT2 | BCS1L |
| PIK3CD | HMGCS2 |
| MIR215 | REN |
| RUNX2 | SOD1 |
| MIR10B | SDHB |
| MIR223 | GAS5 |
| APC | HFE |
| FGFR2 | IGFBP1 |
| SNHG1 | GBA1 |
| ARID1A | MTOR |
| BIRC5 | ENSG00000277577 |
| PCAT1 | GPI |
| SOD2-OT1 | LIPE |
| ESR1 | ACAT1 |
| ENSG00000276609 | LRPPRC |
| ENSG00000277469 | MIR22 |
| ENSG00000277553 | HCFC1 |
| ENSG00000278708 | MIRLET7C |
| MDM2 | MIR27A |
| KLF4 | CCL2 |
| ITGB1 | NPC1 |
| JAK2 | LPIN1 |
| NORAD | ESR1 |
| MIR26B | MIRLET7D |
| RELA | EMSLR |
| PTGS2 | ALDH6A1 |
| HDAC1 | HK1 |
| ID1 | ATP7B |
| SNHG6 | HMOX1 |
| MIR320A | MCCC1 |
| WNT5A | NAMPT |
| CCND3 | TCF7L2 |
| HAGLR | NQO1 |
| RHOA | SOX2 |
| CDKN2B | COX5A |
| LINC01618 | KCNJ11 |
| BAX | PRDX1 |
| MIR494 | SCO2 |
| MIR185 | MALAT1 |
| MIR155 | CUL3 |
| MIR31HG | PIK3CA |
| CDKN2B-AS1 | TTR |
| KCNQ1OT1 | MIR33B |
| KRT7 | CYP27A1 |
| MSH2 | MIR29A |
| SMAD7 | TF |
| MIR100 | MAOA |
| CCND2 | DHCR7 |
| PCNA | MIR145 |
| MIR10A | IDH1 |
| MAPK3 | MT-ATP6 |
| SOX9 | TRA-TGC7-1 |
| IDH1 | TRA-TGC5-1 |
| CXCL8 | MIR140 |
| MIR146B | EPHX2 |
| DNMT1 | PFKL |
| MIR23A | FABP4 |
| CUL1 | NPY |
| WEE1 | AR |
| EZR | ECHS1 |
| NOTCH2 | FGF21 |
| CASC2 | MIRLET7A1 |
| MIR214 | ALOX5 |
| BDNF-AS | ATP5F1A |
| SKP2 | UCP1 |
| RNY3 | EHHADH |
| SHH | GLUL |
| SOX2 | SCD |
| PGR-AS1 | PFKM |
| MIR99A | PDK4 |
| SNHG12 | MIR143 |
| JUN | ANGPTL3 |
| MIR23B | SDHA |
| MAPK14 | SHMT2 |
| SMARCA4 | ALDH4A1 |
| MIR451A | PTGS2 |
| CD274 | PDP1 |
| MMP14 | ABCD1 |
| MCRIP1 | GRHPR |
| MIR222 | PCK1 |
| MIR106B | ADRB2 |
| CXCR4 | ETHE1 |
| PLK1 | MIR30A |
| NEAT1 | MT-CO1 |
| MIR381 | PVT1 |
| LINC01133 | COX6B1 |
| LINC00958 | EPRS1 |
| SCUBE3 | SLC37A4 |
| MIR96 | MIR146B |
| NNT-AS1 | CYP7B1 |
| CBR3-AS1 | RYR1 |
| KRT18 | KL |
| PPP2CA | STAR |
| PLAUR | SLC25A4 |
| CDK7 | GNAS |
| MIR211 | TAFAZZIN |
| MIR183 | MIR27B |
| SPARC | SLC25A20 |
| MIR29A | SIRT3 |
| SNORD15A | IGF2 |
| TBXT | PNPLA3 |
| EPCAM | GAA |
| PPARG | SOD2 |
| IL1B | PRL |
| MIR182 | ACLY |
| CCNB2 | TALDO1 |
| BSG | TKT |
| LOXL2 | APP |
| MIAT | MIR29C |
| MKI67 | PGK1 |
| CAV1 | MDH2 |
| TNC | ACADSB |
| AKT2 | TGFB1 |
| ENSG00000274760 | PRKAB1 |
| ENSG00000277967 | SCARNA5 |
| MIR199A1 | SCARB1 |
| CCN2 | TRE-TTC3-1 |
| RAC1 | SHMT1 |
| IGF1R | PGR-AS1 |
| MIR29C | ALDH9A1 |
| SOX4 | STAT3 |
| TGFB3 | KLF4 |
| BMP7 | ATM |
| BRCA2 | AKT2 |
| PLAU | NDUFS1 |
| VCP | AHR |
| MIR27A | PKLR |
| MTDH | TLR4 |
| G6PD | RAB4B-EGLN2 |
| MIR148A | MT-CO2 |
| PROM1 | GLS2 |
| TRC-GCA24-1 | SECISBP2 |
| KRT19 | ACADL |
| CXCL12 | LONP1 |
| MSH6 | BRAF |
| TERT | OAT |
| PWAR1 | BDNF |
| HMGB1 | NR1H3 |
| GHET1 | NADK2 |
| MIR199B | HNF1A |
| FOXC1 | VEGFA |
| POU5F1 | ANGPTL4 |
| KDM1A | BCAT2 |
| CLDN1 | PYCR1 |
| U2AF1 | DNM1L |
| BUB1B | MPO |
| WT1 | AK2 |
| TRA-TGC7-1 | MAN2B1 |
| TRA-TGC5-1 | MIR130A |
| BTRC | MLXIPL |
| RXRA | SFTA3 |
| SMURF2 | GOT1 |
| TWIST2 | TPI1 |
| MAPK8 | MIR23A |
| MIR106A | BMP6 |
| MUC1 | MT-RNR1 |
| MIR27B | MIR148A |
| KDR | MIR181A1 |
| IGF1 | NR5A1 |
| CCL2 | GSR |
| MIR193A | NDUFS4 |
| NANOG | MIR30E |
| CDC20 | CAD |
| MIRLET7G | GNE |
| METTL3 | RXRA |
| PTHLH | PCK2 |
| MIR429 | TH |
| FEZF1-AS1 | MIR223 |
| SF3B1 | MIR493HG |
| MIR497 | ENSG00000276919 |
| HGF | CTNNB1 |
| MIR22 | MIR146A |
| WNT1 | STK11 |
| FOXQ1 | MIR221 |
| PTK2 | CLOCK |
| MIR146A | MIR222 |
| MIR98 | SOAT1 |
| MSN | PNPLA2 |
| MIR26A1 | SREBF2 |
| MIR296 | MIRLET7B |
| FGF2 | MIR125B1 |
| KLK3 | PGM1 |
| YBX1 | ALDH1A1 |
| KRT20 | MDH1 |
| GSTP1 | PDHB |
| MIR204 | DLAT |
| CDC25A | ALDH3A2 |
| MIRLET7D | NDUFV1 |
| FOXC2 | CFTR |
| TLR4 | ALDOA |
| LGALS1 | EIF2AK3 |
| MIR33B | TRC-GCA24-1 |
| TJP1 | KYNU |
| TBX2 | FOXO1 |
| GATA3 | AGL |
| CLDN7 | HSP90AA1 |
| WNT3A | SNORD15A |
| BMP6 | NEU1 |
| DICER1 | CAV1 |
| MIR30E | AASS |
| MIR9-1 | FGFR1 |
| MIRLET7B | SQSTM1 |
| PRECSIT | MPC1 |
| EPHB2 | SLC1A5 |
| MIR210 | RMRP |
| ELF3 | TYMP |
| MIR19A | PAICS |
| KIT | IGF2R |
| MLH1 | ALDH18A1 |
| BCAR4 | HBB |
| MIR140 | GBE1 |
| ESRP1 | SUCLG1 |
| SPP1 | IDUA |
| MIR126 | CDKN2A |
| POSTN | AMT |
| MIR424 | MIR142 |
| AXIN2 | MIR200A |
| CEMIP | GLP1R |
| RET | KCNQ1OT1 |
| ALK | AKR1C3 |
| CASC15 | PFKP |
| KRT8 | MB |
| MIR543 | PRODH |
| MIR15A | ODC1 |
| MIR29B2 | TNFSF11 |
| JUP | PTPN1 |
| CCR7 | MIR214 |
| MIR142 | ACOX1 |
| MIR498 | MFN2 |
| AKT3 | PDX1 |
| PRKCA | MT-CYB |
| BCYRN1 | MIR25 |
| MIR375 | MIR93 |
| FABP4 | ALDH7A1 |
| MIR29B1 | DNMT3A |
| PANDAR | H6PD |
| MIR20A | GPX1 |
| PTTG1 | MIR335 |
| RNU6-1 | CA2 |
| RNU6-2 | ABAT |
| RNU6-9 | TIGAR |
| MIR137 | CDKN2B-AS1 |
| RNU6-7 | SCN1A |
| RNU6-8 | PIK3R1 |
| RNU6-1-001 | COX4I1 |
| RNU6-1-002 | LBR |
| RNU6-1-003 | EPO |
| RNU6-1-004 | AGPAT2 |
| TEAD4 | MT-TK |
| NOTCH3 | NSDHL |
| CA9 | MAPK1 |
| CCNE2 | DCXR |
| MIR655 | FOXO3 |
| ENSG00000277966 | MIR342 |
| ENSG00000278592 | MIR9-1 |
| ENSG00000273961 | PLA2G4A |
| ENSG00000276496 | OXCT1 |
| ENSG00000278020 | UQCRFS1 |
| ENSG00000278334 | ACO2 |
| BICDL3P | SLC9A3 |
| AFAP1-AS1 | ABCC1 |
| HDAC2 | PFKFB3 |
| CUL7 | UQCRC2 |
| ABL1 | DNMT1 |
| HSP90AA1 | SIRT6 |
| EPAS1 | MIRLET7E |
| HNF1B | YAP1 |
| FOXA1 | TFRC |
| BLACAT1 | GFPT1 |
| OIP5-AS1 | GM2A |
| TOP2A | IFNG |
| NRP1 | SLC25A12 |
| BMP2 | MIR378A |
| CTNNA1 | HRAS |
| MIR192 | GLA |
| SNORD44 | DGAT1 |
| MIR25 | AK1 |
| MIR130B | MCOLN1 |
| SLC25A25-AS1 | COX8A |
| CDC16 | MIR150 |
| EMSLR | ICAM1 |
| MIRLET7A1 | TPO |
| UBC | PDK1 |
| NME1 | GYS1 |
| DUXAP9 | MIR144 |
| SMAD5-AS1 | TNFRSF11B |
| MIR139 | B2M |
| PKM | MIR210 |
| WSPAR | NFU1 |
| PTPN11 | GFM1 |
| FAP | DLEU2 |
| BRD4 | AKR1B1 |
| KLF17 | ERCC2 |
| ZEB1-AS1 | PARP1 |
| PAK1 | HULC |
| TGM2 | NNMT |
| SERPINE1 | SI |
| SIX1 | CREBBP |
| STK11 | ACSL4 |
| CDKN3 | IDO1 |
| LEF1 | ME1 |
| MIR32 | PGD |
| ENO2 | TUFM |
| MTA1 | SLC25A10 |
| CUL4A | ALOX12 |
| PRRX1 | DSP |
| TCF3 | ABHD5 |
| SERPINB5 | SPP1 |
| GREM1 | ATAD3A |
| PKMYT1 | HLA-B |
| VIM-AS1 | PRKN |
| PARP1 | SLC7A5 |
| MIR18A | SLC2A3 |
| MIR127 | MIR15A |
| SKP1 | FABP5 |
| ERCC2 | ERCC6 |
| MIR302A | MIR199A1 |
| LINC00673 | BMAL1 |
| NDRG1 | LINC01554 |
| OVOL2 | PTPN11 |
| CD36 | CXCL8 |
| ITGA5 | CEBPA |
| ETS1 | ENO1 |
| MIR452 | AKR1C2 |
| CTNND1 | MIR200C |
| RBBP4 | MIR195 |
| LINC00511 | MIR182 |
| MIR124-1 | MIRLET7I |
| MSX2 | ESRRA |
| SNORD118 | NOS2 |
| FOS | MIR124-1 |
| PGM5-AS1 | HSD17B4 |
| NLRP3 | CRAT |
| ANXA2 | GYS2 |
| JAG1 | CYCS |
| TGFA | SNCA |
| VDAC1 | MIR31 |
| MIR379 | PCSK1 |
| MIR144 | SLC16A4 |
| CHEK1 | SUCLA2 |
| THBD | TSC2 |
| CDC27 | PDK3 |
| MIR150 | HK3 |
| MIR103A1 | ATP5F1D |
| XBP1 | MIR127 |
| PTCH1 | GALNT2 |
| WNT4 | ALDH1B1 |
| LINC02605 | WRN |
| FHIT | DGUOK |
| NUS1 | ACO1 |
| CCEPR | DLST |
| BECN1 | MGAM |
| MIR149 | MIR532 |
| MIR16-1 | TXNIP |
| TNS4 | DECR1 |
| MIR206 | RORA |
| MIR93 | DHCR24 |
| ERBB4 | TNXB |
| HDAC3 | ATIC |
| PWAR4 | OPA1 |
| FOSL1 | BRCA1 |
| HSPA5 | MIR20A |
| GJA1 | FTL |
| SUZ12 | KCNJ5 |
| LINC00312 | PHGDH |
| MIR128-2 | PLA2G6 |
| SNHG7 | WFS1 |
| RASSF1 | MIR23B |
| LINC00472 | TRP-AGG2-5 |
| LCN2 | ATP5F1E |
| RGCC | TRP-AGG2-6 |
| MIR22HG | TRP-AGG2-1 |
| XPO1 | TRP-AGG2-2 |
| CDK5 | TRP-AGG2-3 |
| MIR15B | TRP-AGG2-4 |
| ABCB1 | TRP-AGG2-7 |
| TCF7L2 | TRP-AGG2-8 |
| MIR216A | NLRP3 |
| HNF4A | VCP |
| EGR1 | MTHFD1L |
| CASC9 | MAPK8 |
| MIR181A1 | MIR212 |
| MIR503 | GART |
| PPIF | MIR133B |
| DNM3OS | SLX1A-SULT1A3 |
| RPS6KB1 | GATA3 |
| P4HA2 | ACTB |
| MCM2 | GCSH |
| ADAM17 | PLA2G7 |
| AXL | PSPH |
| HSPB1 | CYB5A |
| THBS1 | ARID1A |
| SLC25A4 | LDHB |
| PSME3 | C1QBP |
| ROCK1 | ATF4 |
| FOXD2-AS1 | AKR1C1 |
| IL17A | IAPP |
| LINC00460 | BMP2 |
| FSCN1 | IGF1R |
| SMARCB1 | FXN |
| TTN-AS1 | SIRT2 |
| PRMT5 | EIF2S1 |
| CCNH | NEUROG3 |
| TEAD1 | LIN28A |
| RUNX3 | KEAP1 |
| TINCR | FTH1 |
| NES | PANK2 |
| MIR130A | TERT |
| CLU | ABCG1 |
| CTBP1 | RNY5 |
| LINC00504 | EGFR |
| TTTY10 | MIR193B |
| LINC02882 | MIR130B |
| ARF4-AS1 | SCP2 |
| LINC01258 | FABP1 |
| LINC01725 | MMP1 |
| LINC02955 | MIR302A |
| LINC02895 | WWOX |
| ENSG00000230490 | PPA2 |
| ENSG00000245768 | KDM4C |
| ENSG00000250519 | HKDC1 |
| ENSG00000258081 | MIR30D |
| ENSG00000235450 | GSK3B |
| ENSG00000251216 | NDUFS7 |
| LOC101927560 | HSPA8 |
| ENSG00000253288 | LRP1 |
| lnc-IQCM-2 | MEN1 |
| lnc-HMGXB4-8 | MAT2A |
| MK280073-022 | SLC9A1 |
| MK280073-023 | ZMPSTE24 |
| MK280073-058 | FGFR4 |
| MK280073-120 | HMGA1 |
| MK280073-197 | MAPK14 |
| MK280073-202 | FOXK1 |
| MK280073-346 | NRF1 |
| MK280073-501 | SMARCA4 |
| MK280073-521 | SHPK |
| MK280073-008 | SPTLC1 |
| MK280073-013 | CCND1 |
| MK280073-020 | MIR499A |
| MK280073-025 | MTHFD2 |
| MK280073-049 | PPAT |
| MK280073-052 | FOXA2 |
| MK280073-055 | CDKN3 |
| MK280073-059 | MIR103A1 |
| MK280073-060 | TSFM |
| MK280073-063 | PPARGC1B |
| MK280073-083 | NDUFA6 |
| MK280073-093 | GMPS |
| MK280073-121 | RB1 |
| MK280073-164 | BRCA2 |
| MK280073-175 | GCH1 |
| MK280073-199 | VDAC1 |
| MK280073-203 | NDUFS2 |
| MK280073-206 | PIK3CG |
| MK280073-216 | PSEN1 |
| MK280073-273 | KCNH2 |
| MK280073-296 | KCNQ1 |
| MK280073-300 | IL18 |
| MK280073-334 | GNA11 |
| MK280073-351 | NDUFA13 |
| MK280073-353 | SLC25A1 |
| MK280073-460 | PEX10 |
| MK280073-461 | MTO1 |
| MK280073-475 | KARS1 |
| MK280073-493 | HNF1B |
| MK280073-510 | TFAM |
| MK280073-513 | PCAT1 |
| MK280073-522 | YARS2 |
| MK280073-524 | MIR34C |
| MK280073-527 | MIR26A1 |
| MK280073-571 | MIR139 |
| MK280073-621 | CLPB |
| MK280073-623 | CTSD |
| MK280073-001 | CHKA |
| MK280073-007 | COA6 |
| MK280073-018 | BCL2 |
| MK280073-027 | NKX2-1 |
| MK280073-028 | TXN |
| MK280073-030 | MIR133A1 |
| MK280073-037 | SLC4A4 |
| MK280073-040 | SLC5A5 |
| MK280073-044 | EP300 |
| MK280073-046 | HNF1A-AS1 |
| MK280073-047 | FOXP3 |
| MK280073-051 | DRD2 |
| MK280073-053 | APPL1 |
| MK280073-056 | ENO3 |
| MK280073-057 | MIR24-1 |
| MK280073-071 | PRKAG1 |
| MK280073-072 | GPAM |
| MK280073-073 | NDUFS8 |
| MK280073-075 | PTGS1 |
| MK280073-079 | TNFRSF11A |
| MK280073-081 | DIO2 |
| MK280073-082 | HOTTIP |
| MK280073-089 | STXBP1 |
| MK280073-090 | MIR204 |
| MK280073-091 | PON2 |
| MK280073-092 | AIFM1 |
| MK280073-103 | NDUFS3 |
| MK280073-106 | PRKACA |
| MK280073-115 | FDXR |
| MK280073-116 | AFP |
| MK280073-117 | NRAS |
| MK280073-119 | ASPH |
| MK280073-123 | TET2 |
| MK280073-125 | MME |
| MK280073-140 | UQCRQ |
| MK280073-149 | MIR193A |
| MK280073-150 | SOCS1 |
| MK280073-153 | UQCRB |
| MK280073-154 | CARM1 |
| MK280073-168 | TERC |
| MK280073-174 | PSAT1 |
| MK280073-176 | PNPT1 |
| MK280073-179 | GUCY2C |
| MK280073-180 | LRP5 |
| MK280073-182 | ARNT |
| MK280073-183 | DNMT3B |
| MK280073-185 | MIR330 |
| MK280073-190 | HSPA4 |
| MK280073-194 | ASNS |
| MK280073-205 | SETD2 |
| MK280073-207 | CPOX |
| MK280073-208 | SLC27A1 |
| MK280073-221 | CD46 |
| MK280073-233 | RELA |
| MK280073-243 | HSPA1A |
| MK280073-247 | FGFR2 |
| MK280073-258 | TM6SF2 |
| MK280073-280 | TIMP1 |
| MK280073-283 | MIR338 |
| MK280073-295 | ATG7 |
| MK280073-301 | MIR28 |
| MK280073-302 | EPAS1 |
| MK280073-313 | MIR451A |
| MK280073-345 | MIR203A |
| MK280073-349 | MIR30B |
| MK280073-352 | NFKB1 |
| MK280073-354 | ISCA2 |
| MK280073-355 | JUN |
| MK280073-357 | PTGIS |
| MK280073-359 | CYB5R3 |
| MK280073-364 | IDH3A |
| MK280073-384 | PGR |
| MK280073-389 | MGLL |
| MK280073-396 | SARS2 |
| MK280073-397 | EZH2 |
| MK280073-414 | CCAT1 |
| MK280073-434 | SMARCA2 |
| MK280073-445 | MAPT |
| MK280073-453 | PGAM1 |
| MK280073-455 | HNRNPA1 |
| MK280073-456 | ENSG00000275307 |
| MK280073-464 | ENSG00000276965 |
| MK280073-465 | HNRNPA2B1 |
| MK280073-466 | CD44 |
| MK280073-467 | MIR486-1 |
| MK280073-468 | MIR15B |
| MK280073-470 | ACSL1 |
| MK280073-473 | NPAS2 |
| MK280073-476 | HSPD1 |
| MK280073-486 | HSPG2 |
| MK280073-487 | SIRT4 |
| MK280073-490 | STX3 |
| MK280073-495 | FOS |
| MK280073-496 | NEUROD1 |
| MK280073-499 | PEX1 |
| MK280073-500 | MT-TW |
| MK280073-512 | HDAC1 |
| MK280073-515 | MIRLET7G |
| MK280073-516 | AXIN1 |
| MK280073-519 | PBX1 |
| MK280073-523 | TUG1 |
| MK280073-525 | C9orf72 |
| MK280073-530 | MTAP |
| MK280073-531 | CAVIN1 |
| MK280073-532 | HTRA2 |
| MK280073-533 | SPR |
| MK280073-538 | GSTO1 |
| MK280073-540 | PRDX2 |
| MK280073-543 | MIAT |
| MK280073-544 | STXBP2 |
| MK280073-551 | CRY2 |
| MK280073-554 | MET |
| MK280073-556 | SLC25A3 |
| MK280073-557 | VHL |
| MK280073-558 | DNAH8 |
| MK280073-559 | MIR29B1 |
| MK280073-561 | CPT1C |
| MK280073-564 | RIPK3 |
| MK280073-574 | HMOX2 |
| MK280073-578 | GNPAT |
| MK280073-585 | NR1D2 |
| MK280073-586 | FLT3 |
| MK280073-590 | MIR98 |
| MK280073-592 | SP1 |
| MK280073-593 | FOXM1 |
| MK280073-596 | TRN-GTT2-1 |
| MK280073-597 | TRN-GTT2-5 |
| MK280073-609 | TRN-GTT2-6 |
| MK280073-618 | TRN-GTT2-2 |
| MK280073-655 | TRN-GTT2-3 |
| MK280073-659 | TRN-GTT2-4 |
| MK280073-688 | TRN-GTT2-7 |
| MK280073-689 | TRN-GTT2-8 |
| lnc-LRP5L-15 | ESR2 |
| MK280073-002 | MMP2 |
| MK280073-003 | MIR92A1 |
| MK280073-004 | IL2 |
| MK280073-005 | SQOR |
| MK280073-006 | GLO1 |
| MK280073-009 | CD38 |
| MK280073-010 | LINC00504 |
| MK280073-011 | KIT |
| MK280073-012 | FABP3 |
| MK280073-014 | MIR18A |
| MK280073-015 | CRH |
| MK280073-016 | THBS1 |
| MK280073-017 | PYCR2 |
| MK280073-019 | MUC1 |
| MK280073-021 | NDUFB10 |
| MK280073-024 | NT5E |
| MK280073-026 | MIR196A1 |
| MK280073-029 | DVL2 |
| MK280073-031 | LINC01258 |
| MK280073-032 | LINC01725 |
| MK280073-033 | RUNX2 |
| MK280073-034 | ACOT1 |
| MK280073-035 | APC |
| MK280073-036 | LINC02882 |
| MK280073-038 | ARF4-AS1 |
| MK280073-039 | LINC02955 |
| MK280073-041 | LINC02895 |
| MK280073-042 | ENSG00000230490 |
| MK280073-043 | ENSG00000245768 |
| MK280073-045 | ENSG00000250519 |
| MK280073-048 | ENSG00000258081 |
| MK280073-050 | ENSG00000235450 |
| MK280073-054 | ENSG00000251216 |
| MK280073-061 | LOC101927560 |
| MK280073-062 | ENSG00000253288 |
| MK280073-064 | lnc-IQCM-2 |
| MK280073-065 | lnc-HMGXB4-8 |
| MK280073-066 | MK280073-022 |
| MK280073-068 | MK280073-023 |
| MK280073-069 | MK280073-058 |
| MK280073-070 | MK280073-008 |
| MK280073-074 | MK280073-013 |
| MK280073-076 | MK280073-020 |
| MK280073-077 | MK280073-025 |
| MK280073-078 | MK280073-049 |
| MK280073-080 | MK280073-052 |
| MK280073-084 | MK280073-055 |
| MK280073-085 | MK280073-059 |
| MK280073-086 | MK280073-060 |
| MK280073-087 | MK280073-063 |
| MK280073-088 | MK280073-083 |
| MK280073-094 | MK280073-001 |
| MK280073-095 | MK280073-007 |
| MK280073-096 | MK280073-018 |
| MK280073-097 | MK280073-027 |
| MK280073-098 | MK280073-028 |
| MK280073-101 | MK280073-030 |
| MK280073-102 | MK280073-037 |
| MK280073-104 | MK280073-040 |
| MK280073-105 | MK280073-044 |
| MK280073-107 | MK280073-046 |
| MK280073-108 | MK280073-047 |
| MK280073-109 | MK280073-051 |
| MK280073-110 | MK280073-053 |
| MK280073-111 | MK280073-056 |
| MK280073-112 | MK280073-057 |
| MK280073-113 | MK280073-071 |
| MK280073-114 | MK280073-072 |
| MK280073-118 | MK280073-073 |
| MK280073-122 | MK280073-075 |
| MK280073-124 | MK280073-079 |
| MK280073-126 | MK280073-081 |
| MK280073-127 | MK280073-082 |
| MK280073-128 | MK280073-089 |
| MK280073-131 | lnc-LRP5L-15 |
| MK280073-132 | MK280073-002 |
| MK280073-133 | MK280073-003 |
| MK280073-134 | MK280073-004 |
| MK280073-135 | MK280073-005 |
| MK280073-136 | MK280073-006 |
| MK280073-137 | MK280073-009 |
| MK280073-138 | MK280073-010 |
| MK280073-139 | MK280073-011 |
| MK280073-141 | MK280073-012 |
| MK280073-142 | MK280073-014 |
| MK280073-143 | MK280073-015 |
| MK280073-144 | MK280073-016 |
| MK280073-145 | MK280073-017 |
| MK280073-146 | MK280073-019 |
| MK280073-147 | MK280073-021 |
| MK280073-148 | MK280073-024 |
| MK280073-151 | MK280073-026 |
| MK280073-152 | MK280073-029 |
| MK280073-155 | MK280073-031 |
| MK280073-156 | MK280073-032 |
| MK280073-157 | MK280073-033 |
| MK280073-158 | MK280073-034 |
| MK280073-159 | MK280073-035 |
| MK280073-160 | MK280073-036 |
| MK280073-161 | MK280073-038 |
| MK280073-162 | MK280073-039 |
| MK280073-163 | MK280073-041 |
| MK280073-165 | MK280073-042 |
| MK280073-169 | MK280073-043 |
| MK280073-170 | MK280073-045 |
| MK280073-171 | MK280073-048 |
| MK280073-172 | MK280073-050 |
| MK280073-173 | MK280073-054 |
| MK280073-177 | MK280073-061 |
| MK280073-178 | MK280073-062 |
| MK280073-181 | MK280073-064 |
| MK280073-184 | MK280073-065 |
| MK280073-186 | MK280073-066 |
| MK280073-187 | MK280073-068 |
| MK280073-188 | MK280073-069 |
| MK280073-189 | MK280073-070 |
| MK280073-191 | MK280073-074 |
| MK280073-192 | MK280073-076 |
| MK280073-193 | MK280073-077 |
| MK280073-195 | MK280073-078 |
| MK280073-196 | MK280073-080 |
| MK280073-198 | MK280073-084 |
| MK280073-200 | MK280073-085 |
| MK280073-201 | MK280073-086 |
| MK280073-204 | MK280073-087 |
| MK280073-209 | MK280073-088 |
| MK280073-210 | MK280073-067 |
| MK280073-211 | NDUFA9 |
| MK280073-212 | SMAD3 |
| MK280073-213 | DYRK1A |
| MK280073-214 | BOLA3 |
| MK280073-215 | PDK2 |
| MK280073-217 | MIR574 |
| MK280073-218 | YWHAE |
| MK280073-219 | NDUFB9 |
| MK280073-220 | AMPD2 |
| MK280073-222 | MECP2 |
| MK280073-224 | FUS |
| MK280073-225 | SLC25A11 |
| MK280073-226 | NDUFB11 |
| MK280073-227 | MIR199B |
| MK280073-228 | AGA |
| MK280073-229 | PINK1 |
| MK280073-230 | SLC3A2 |
| MK280073-231 | GPX4 |
| MK280073-232 | FDX1 |
| MK280073-234 | CDKN2B |
| MK280073-235 | SAT1 |
| MK280073-236 | NDUFC2 |
| MK280073-237 | GPT2 |
| MK280073-238 | COA3 |
| MK280073-239 | PFAS |
| MK280073-240 | PIK3CB |
| MK280073-241 | NDUFA4 |
| MK280073-242 | MMP9 |
| MK280073-244 | PRDX5 |
| MK280073-245 | TTTY10 |
| MK280073-246 | PLAAT1 |
| MK280073-248 | LAMP2 |
| MK280073-249 | SLC7A11 |
| MK280073-250 | PTPN22 |
| MK280073-251 | IL6R |
| MK280073-252 | IMPDH2 |
| MK280073-253 | BCAP31 |
| MK280073-255 | UGP2 |
| MK280073-257 | PPP1CA |
| MK280073-259 | HNRNPK |
| MK280073-260 | STAT1 |
| MK280073-261 | JAK2 |
| MK280073-262 | OGT |
| MK280073-264 | TREM2 |
| MK280073-265 | NEAT1 |
| MK280073-266 | NANOG |
| MK280073-267 | JPX |
| MK280073-268 | HMGCS1 |
| MK280073-269 | MIR99A |
| MK280073-270 | NCOA2 |
| MK280073-271 | PLIN2 |
| MK280073-272 | EGF |
| MK280073-274 | GATA6 |
| MK280073-275 | LTA4H |
| MK280073-276 | TET3 |
| MK280073-277 | SNHG1 |
| MK280073-278 | HSP90B1 |
| MK280073-279 | LCN2 |
| MK280073-281 | NR5A2 |
| MK280073-282 | LPCAT1 |
| MK280073-284 | MPI |
| MK280073-285 | SCN5A |
| MK280073-286 | COQ9 |
| MK280073-287 | UAP1 |
| MK280073-288 | MK280073-120 |
| MK280073-289 | MK280073-197 |
| MK280073-290 | MK280073-202 |
| MK280073-291 | MK280073-346 |
| MK280073-292 | MK280073-501 |
| MK280073-293 | MK280073-521 |
| MK280073-294 | MK280073-093 |
| MK280073-297 | MK280073-121 |
| MK280073-298 | MK280073-164 |
| MK280073-299 | MK280073-175 |
| MK280073-303 | MK280073-199 |
| MK280073-304 | MK280073-203 |
| MK280073-305 | MK280073-206 |
| MK280073-306 | MK280073-216 |
| MK280073-307 | MK280073-273 |
| MK280073-308 | MK280073-296 |
| MK280073-309 | MK280073-300 |
| MK280073-310 | MK280073-334 |
| MK280073-311 | MK280073-351 |
| MK280073-312 | MK280073-353 |
| MK280073-314 | MK280073-460 |
| MK280073-315 | MK280073-461 |
| MK280073-316 | MK280073-475 |
| MK280073-317 | MK280073-493 |
| MK280073-318 | MK280073-510 |
| MK280073-319 | MK280073-513 |
| MK280073-320 | MK280073-522 |
| MK280073-321 | MK280073-524 |
| MK280073-322 | MK280073-527 |
| MK280073-323 | MK280073-571 |
| MK280073-324 | MK280073-621 |
| MK280073-325 | MK280073-623 |
| MK280073-326 | MK280073-090 |
| MK280073-328 | MK280073-091 |
| MK280073-329 | MK280073-092 |
| MK280073-330 | MK280073-103 |
| MK280073-331 | MK280073-106 |
| MK280073-332 | MK280073-115 |
| MK280073-333 | MK280073-116 |
| MK280073-335 | MK280073-117 |
| MK280073-336 | MK280073-119 |
| MK280073-337 | MK280073-123 |
| MK280073-338 | MK280073-125 |
| MK280073-339 | MK280073-140 |
| MK280073-340 | MK280073-149 |
| MK280073-341 | MK280073-150 |
| MK280073-342 | MK280073-153 |
| MK280073-343 | MK280073-154 |
| MK280073-344 | MK280073-168 |
| MK280073-347 | MK280073-174 |
| MK280073-348 | MK280073-176 |
| MK280073-350 | MK280073-179 |
| MK280073-356 | MK280073-180 |
| MK280073-358 | MK280073-182 |
| MK280073-360 | MK280073-183 |
| MK280073-361 | MK280073-185 |
| MK280073-362 | MK280073-190 |
| MK280073-363 | MK280073-194 |
| MK280073-365 | MK280073-205 |
| MK280073-366 | MK280073-207 |
| MK280073-367 | MK280073-208 |
| MK280073-368 | MK280073-221 |
| MK280073-369 | MK280073-233 |
| MK280073-370 | MK280073-243 |
| MK280073-371 | MK280073-247 |
| MK280073-372 | MK280073-258 |
| MK280073-373 | MK280073-280 |
| MK280073-374 | MK280073-283 |
| MK280073-375 | MK280073-295 |
| MK280073-376 | MK280073-301 |
| MK280073-377 | MK280073-302 |
| MK280073-378 | MK280073-313 |
| MK280073-379 | MK280073-345 |
| MK280073-380 | MK280073-349 |
| MK280073-381 | MK280073-352 |
| MK280073-382 | MK280073-354 |
| MK280073-383 | MK280073-355 |
| MK280073-385 | MK280073-357 |
| MK280073-386 | MK280073-359 |
| MK280073-387 | MK280073-364 |
| MK280073-388 | MK280073-384 |
| MK280073-390 | MK280073-389 |
| MK280073-391 | MK280073-396 |
| MK280073-395 | MK280073-397 |
| MK280073-398 | MK280073-414 |
| MK280073-399 | MK280073-434 |
| MK280073-402 | MK280073-445 |
| MK280073-403 | MK280073-453 |
| MK280073-404 | MK280073-455 |
| MK280073-405 | MK280073-456 |
| MK280073-406 | MK280073-464 |
| MK280073-407 | MK280073-465 |
| MK280073-409 | MK280073-466 |
| MK280073-410 | MK280073-467 |
| MK280073-411 | MK280073-468 |
| MK280073-412 | MK280073-470 |
| MK280073-413 | MK280073-473 |
| MK280073-415 | MK280073-476 |
| MK280073-416 | MK280073-486 |
| MK280073-417 | MK280073-487 |
| MK280073-418 | MK280073-490 |
| MK280073-419 | MK280073-495 |
| MK280073-420 | MK280073-496 |
| MK280073-427 | MK280073-499 |
| MK280073-428 | MK280073-500 |
| MK280073-430 | MK280073-512 |
| MK280073-431 | MK280073-515 |
| MK280073-432 | MK280073-516 |
| MK280073-433 | MK280073-519 |
| MK280073-435 | MK280073-523 |
| MK280073-436 | MK280073-525 |
| MK280073-437 | MK280073-530 |
| MK280073-438 | MK280073-531 |
| MK280073-439 | MK280073-532 |
| MK280073-440 | MK280073-533 |
| MK280073-441 | MK280073-538 |
| MK280073-442 | MK280073-540 |
| MK280073-443 | MK280073-543 |
| MK280073-444 | MK280073-544 |
| MK280073-446 | MK280073-551 |
| MK280073-447 | MK280073-554 |
| MK280073-448 | MK280073-556 |
| MK280073-449 | MK280073-557 |
| MK280073-450 | MK280073-558 |
| MK280073-451 | MK280073-559 |
| MK280073-452 | MK280073-561 |
| MK280073-454 | MK280073-564 |
| MK280073-457 | MK280073-574 |
| MK280073-458 | MK280073-578 |
| MK280073-459 | MK280073-585 |
| MK280073-462 | MK280073-586 |
| MK280073-463 | MK280073-590 |
| MK280073-469 | MK280073-592 |
| MK280073-471 | MK280073-593 |
| MK280073-472 | MK280073-596 |
| MK280073-474 | MK280073-597 |
| MK280073-477 | MK280073-609 |
| MK280073-478 | MK280073-618 |
| MK280073-479 | MK280073-655 |
| MK280073-480 | MK280073-659 |
| MK280073-481 | MK280073-688 |
| MK280073-482 | MK280073-689 |
| MK280073-483 | MK280073-094 |
| MK280073-484 | MK280073-095 |
| MK280073-485 | MK280073-096 |
| MK280073-488 | MK280073-097 |
| MK280073-491 | MK280073-098 |
| MK280073-492 | MK280073-101 |
| MK280073-494 | MK280073-102 |
| MK280073-497 | MK280073-104 |
| MK280073-498 | MK280073-105 |
| MK280073-502 | MK280073-107 |
| MK280073-503 | MK280073-108 |
| MK280073-504 | MK280073-109 |
| MK280073-505 | MK280073-110 |
| MK280073-506 | MK280073-111 |
| MK280073-507 | MK280073-112 |
| MK280073-508 | MK280073-113 |
| MK280073-509 | MK280073-114 |
| MK280073-511 | MK280073-118 |
| MK280073-514 | MK280073-122 |
| MK280073-517 | MK280073-124 |
| MK280073-518 | MK280073-126 |
| MK280073-526 | MK280073-127 |
| MK280073-528 | MK280073-128 |
| MK280073-529 | MK280073-131 |
| MK280073-534 | MK280073-132 |
| MK280073-535 | MK280073-133 |
| MK280073-536 | MK280073-134 |
| MK280073-537 | MK280073-135 |
| MK280073-541 | MK280073-136 |
| MK280073-542 | MK280073-137 |
| MK280073-545 | MK280073-138 |
| MK280073-546 | MK280073-139 |
| MK280073-547 | MK280073-141 |
| MK280073-548 | MK280073-142 |
| MK280073-549 | MK280073-143 |
| MK280073-550 | MK280073-144 |
| MK280073-552 | MK280073-145 |
| MK280073-553 | MK280073-146 |
| MK280073-555 | MK280073-147 |
| MK280073-560 | MK280073-148 |
| MK280073-562 | MK280073-151 |
| MK280073-563 | MK280073-152 |
| MK280073-565 | MK280073-155 |
| MK280073-566 | MK280073-156 |
| MK280073-567 | MK280073-157 |
| MK280073-568 | MK280073-158 |
| MK280073-569 | MK280073-159 |
| MK280073-570 | MK280073-160 |
| MK280073-572 | MK280073-161 |
| MK280073-573 | MK280073-162 |
| MK280073-575 | MK280073-163 |
| MK280073-577 | MK280073-165 |
| MK280073-579 | MK280073-169 |
| MK280073-580 | MK280073-170 |
| MK280073-581 | MK280073-171 |
| MK280073-582 | MK280073-172 |
| MK280073-583 | MK280073-173 |
| MK280073-584 | MK280073-177 |
| MK280073-588 | MK280073-178 |
| MK280073-589 | MK280073-181 |
| MK280073-591 | MK280073-184 |
| MK280073-594 | MK280073-186 |
| MK280073-595 | MK280073-187 |
| MK280073-598 | MK280073-188 |
| MK280073-606 | MK280073-189 |
| MK280073-615 | MK280073-191 |
| MK280073-624 | MK280073-192 |
| MK280073-625 | MK280073-193 |
| MK280073-629 | MK280073-195 |
| MK280073-654 | MK280073-196 |
| MK280073-656 | MK280073-198 |
| MK280073-670 | MK280073-200 |
| MK280073-674 | MK280073-201 |
| MK280073-676 | MK280073-204 |
| MK280073-678 | MK280073-209 |
| MK280073-683 | MK280073-210 |
| MK280073-686 | MK280073-211 |
| MK280073-691 | MK280073-212 |
| MK280073-067 | MK280073-213 |
| MK280073-099 | MK280073-214 |
| MK280073-100 | MK280073-215 |
| MK280073-129 | MK280073-217 |
| MK280073-130 | MK280073-218 |
| MK280073-166 | MK280073-219 |
| MK280073-167 | MK280073-220 |
| MK280073-223 | MK280073-222 |
| MK280073-254 | MK280073-224 |
| MK280073-256 | MK280073-225 |
| MK280073-263 | MK280073-226 |
| MK280073-327 | MK280073-227 |
| MK280073-392 | MK280073-228 |
| MK280073-393 | MK280073-229 |
| MK280073-394 | MK280073-230 |
| MK280073-400 | MK280073-231 |
| MK280073-401 | MK280073-232 |
| MK280073-408 | MK280073-234 |
| MK280073-421 | MK280073-235 |
| MK280073-422 | MK280073-236 |
| MK280073-423 | MK280073-237 |
| MK280073-424 | MK280073-238 |
| MK280073-425 | MK280073-239 |
| MK280073-426 | MK280073-240 |
| MK280073-429 | MK280073-241 |
| MK280073-489 | MK280073-242 |
| MK280073-520 | MK280073-244 |
| MK280073-539 | MK280073-245 |
| MK280073-576 | MK280073-246 |
| MK280073-587 | MK280073-248 |
| MK280073-599 | MK280073-249 |
| MK280073-600 | MK280073-250 |
| MK280073-601 | MK280073-251 |
| MK280073-602 | MK280073-252 |
| MK280073-603 | MK280073-253 |
| MK280073-604 | MK280073-255 |
| MK280073-605 | MK280073-257 |
| MK280073-607 | MK280073-259 |
| MK280073-608 | MK280073-260 |
| MK280073-610 | MK280073-261 |
| MK280073-611 | MK280073-262 |
| MK280073-612 | MK280073-264 |
| MK280073-613 | MK280073-265 |
| MK280073-614 | MK280073-266 |
| MK280073-616 | MK280073-267 |
| MK280073-617 | MK280073-268 |
| MK280073-619 | MK280073-269 |
| MK280073-620 | MK280073-270 |
| MK280073-622 | MK280073-271 |
| MK280073-626 | MK280073-272 |
| MK280073-627 | MK280073-274 |
| MK280073-628 | MK280073-275 |
| MK280073-630 | MK280073-276 |
| MK280073-631 | MK280073-277 |
| MK280073-632 | MK280073-278 |
| MK280073-633 | MK280073-279 |
| MK280073-634 | MK280073-281 |
| MK280073-635 | MK280073-282 |
| MK280073-636 | MK280073-284 |
| MK280073-637 | MK280073-285 |
| MK280073-638 | MK280073-286 |
| MK280073-639 | MK280073-287 |
| MK280073-640 | MK280073-288 |
| MK280073-641 | MK280073-289 |
| MK280073-642 | MK280073-290 |
| MK280073-643 | MK280073-291 |
| MK280073-644 | MK280073-292 |
| MK280073-645 | MK280073-293 |
| MK280073-646 | MK280073-294 |
| MK280073-647 | MK280073-297 |
| MK280073-648 | MK280073-298 |
| MK280073-649 | MK280073-299 |
| MK280073-650 | MK280073-303 |
| MK280073-651 | MK280073-304 |
| MK280073-652 | MK280073-305 |
| MK280073-653 | MK280073-306 |
| MK280073-657 | MK280073-307 |
| MK280073-658 | MK280073-308 |
| MK280073-660 | MK280073-309 |
| MK280073-661 | MK280073-310 |
| MK280073-662 | MK280073-311 |
| MK280073-663 | MK280073-312 |
| MK280073-664 | MK280073-314 |
| MK280073-665 | MK280073-315 |
| MK280073-666 | MK280073-316 |
| MK280073-667 | MK280073-317 |
| MK280073-668 | MK280073-318 |
| MK280073-669 | MK280073-319 |
| MK280073-671 | MK280073-320 |
| MK280073-672 | MK280073-321 |
| MK280073-673 | MK280073-322 |
| MK280073-675 | MK280073-323 |
| MK280073-677 | MK280073-324 |
| MK280073-679 | MK280073-325 |
| MK280073-680 | MK280073-326 |
| MK280073-681 | MK280073-328 |
| MK280073-682 | MK280073-329 |
| MK280073-684 | MK280073-330 |
| MK280073-685 | MK280073-331 |
| MK280073-687 | MK280073-332 |
| MK280073-690 | MK280073-333 |
| MK280073-692 | MK280073-335 |
| MK280073-693 | MK280073-336 |
| MK280073-694 | MK280073-337 |
| MK280073-695 | MK280073-338 |
| MK280073-696 | MK280073-339 |
| MK280073-697 | MK280073-340 |
| MK280073-698 | MK280073-341 |
| MK280073-699 | MK280073-342 |
| MK280073-700 | MK280073-343 |
| MK280073-701 | MK280073-344 |
| MK280073-702 | MK280073-347 |
| MK280073-703 | MK280073-348 |
| MK280073-704 | MK280073-350 |
| MK280073-705 | MK280073-356 |
| MK280073-706 | MK280073-358 |
| MK280073-707 | MK280073-360 |
| MK280073-708 | MK280073-361 |
| MK280073-709 | MK280073-362 |
| MK280073-710 | MK280073-363 |
| MK280073-711 | MK280073-365 |
| MK280073-712 | MK280073-366 |
| MK280073-713 | MK280073-367 |
| MK280073-714 | MK280073-368 |
| MK280073-715 | MK280073-369 |
| MK280073-716 | MK280073-370 |
| MK280073-717 | MK280073-371 |
| MK280073-718 | MK280073-372 |
| MK280073-719 | MK280073-373 |
| MK280073-720 | MK280073-374 |
| MK280073-721 | MK280073-375 |
| MK280073-722 | MK280073-376 |
| MIR9-2 | MK280073-377 |
| SNHG14 | MK280073-378 |
| CDK3 | MK280073-379 |
| APP | MK280073-380 |
| YWHAZ | MK280073-381 |
| E2F3 | MK280073-382 |
| MMP7 | MK280073-383 |
| NTRK2 | MK280073-385 |
| PSCA | MK280073-386 |
| MIR485 | MK280073-387 |
| NUMB | MK280073-388 |
| ABCG2 | MK280073-390 |
| HBEGF | MK280073-391 |
| JUNB | MK280073-395 |
| SQSTM1 | MK280073-398 |
| ID2 | MK280073-399 |
| LNCRNA-ATB | MK280073-402 |
| MIR30B | MK280073-403 |
| FENDRR | MK280073-404 |
| STAT1 | MK280073-405 |
| MIR132 | MK280073-406 |
| EIF5A2 | MK280073-407 |
| NBN | MK280073-409 |
| ESR2 | MK280073-410 |
| GRHL2 | MK280073-411 |
| MMP1 | MK280073-412 |
| PTPN14 | MK280073-413 |
| LATS1 | MK280073-415 |
| RMRP | MK280073-416 |
| SDC1 | MK280073-417 |
| ENSG00000277444 | MK280073-418 |
| SOX5 | MK280073-419 |
| MIR345 | MK280073-420 |
| DNMT3B | MK280073-427 |
| TMPRSS4 | MK280073-428 |
| MAPK7 | MK280073-430 |
| GATA6 | MK280073-431 |
| CDH3 | MK280073-432 |
| CD24 | MK280073-433 |
| MDC1-AS1 | MK280073-435 |
| MIR370 | MK280073-436 |
| ITGAV | MK280073-437 |
| AQP5 | MK280073-438 |
| TM4SF5 | MK280073-439 |
| MUC16 | MK280073-440 |
| GOLM1 | MK280073-441 |
| DDR1 | MK280073-442 |
| NOTCH4 | MK280073-443 |
| MIR101-1 | MK280073-444 |
| CDC42 | MK280073-446 |
| MIR186 | MK280073-447 |
| TTC3 | MK280073-448 |
| NR2F2 | MK280073-449 |
| FOXO1 | MK280073-450 |
| SREBF1 | MK280073-451 |
| IGFBP7 | MK280073-452 |
| KLF5 | MK280073-454 |
| PDCD4 | MK280073-457 |
| MIR486-1 | MK280073-458 |
| BCL2L1 | MK280073-459 |
| ITGA2 | MK280073-462 |
| MIR122 | MK280073-463 |
| GDF15 | MK280073-469 |
| ARRB1 | MK280073-471 |
| FOXO3 | MK280073-472 |
| NPTN-IT1 | MK280073-474 |
| MAD2L2 | MK280073-477 |
| LINC00261 | MK280073-478 |
| DDR2 | MK280073-479 |
| TIAM1 | MK280073-480 |
| EIF4E | MK280073-481 |
| CRYAB | MK280073-482 |
| MIR422A | MK280073-483 |
| MIR495 | MK280073-484 |
| IGF2 | MK280073-485 |
| TYMS | MK280073-488 |
| SPRY2 | MK280073-491 |
| MACC1 | MK280073-492 |
| ACTL6A | MK280073-494 |
| HS3ST3B1 | MK280073-497 |
| IFI27 | MK280073-498 |
| HOXA-AS2 | MK280073-502 |
| MSX1 | MK280073-503 |
| FZD7 | MK280073-504 |
| DAB2IP | MK280073-505 |
| PDGFRA | MK280073-506 |
| BUB1 | MK280073-507 |
| NF2 | MK280073-508 |
| HDAC6 | MK280073-509 |
| GMNN | MK280073-511 |
| LEP | MK280073-514 |
| EPHA2 | MK280073-517 |
| SEMA4C | MK280073-518 |
| MIR506 | MK280073-526 |
| CASP3 | MK280073-528 |
| CEP131 | MK280073-529 |
| MIR129-2 | MK280073-534 |
| SFRP1 | MK280073-535 |
| RAF1 | MK280073-536 |
| MIR125B1 | MK280073-537 |
| SMARCAL1 | MK280073-541 |
| MIR542 | MK280073-542 |
| MIR129-1 | MK280073-545 |
| SOD2 | MK280073-546 |
| MRTFA | MK280073-547 |
| KLF6 | MK280073-548 |
| MIR508 | MK280073-549 |
| HIF1A-AS2 | MK280073-550 |
| PIK3CG | MK280073-552 |
| UHRF1 | MK280073-553 |
| MIR26A2 | MK280073-555 |
| PARD6A | MK280073-560 |
| CTNNA3 | MK280073-562 |
| NONHSAG046336.2 | MK280073-563 |
| AGER | MK280073-565 |
| AOC4P | MK280073-566 |
| SIRT2 | MK280073-567 |
| PGR | MK280073-568 |
| MIR409 | MK280073-569 |
| MIR134 | MK280073-570 |
| FOXA2 | MK280073-572 |
| FAS | MK280073-573 |
| ADAM10 | MK280073-575 |
| CFTR | MK280073-577 |
| MIR224 | MK280073-579 |
| FLNA | MK280073-580 |
| CCL5 | MK280073-581 |
| MIR4435-2HG | MK280073-582 |
| DELEC1 | MK280073-583 |
| CLDN3 | MK280073-584 |
| L1CAM | MK280073-588 |
| TEAD3 | MK280073-589 |
| CD82 | MK280073-591 |
| DAB2 | MK280073-594 |
| RGS3 | MK280073-595 |
| TRIM33 | MK280073-598 |
| MYB | MK280073-606 |
| RPS27A | MK280073-615 |
| MIR202 | MK280073-624 |
| TBX3 | MK280073-625 |
| MIR663A | MK280073-629 |
| APEX1 | MK280073-654 |
| XPC | MK280073-656 |
| LINC00963 | MK280073-670 |
| MDK | MK280073-674 |
| MIR335 | MK280073-676 |
| FOXF1 | MK280073-678 |
| CCN1 | MK280073-683 |
| BDNF | MK280073-686 |
| HEIH | MK280073-691 |
| SATB1 | MK280073-099 |
| RAB4B-EGLN2 | MK280073-100 |
| TACC3 | MK280073-129 |
| TLR2 | MK280073-130 |
| PIM1 | MK280073-166 |
| CUL4B | MK280073-167 |
| RBBP7 | MK280073-223 |
| CTBP2 | MK280073-254 |
| LINC00355 | MK280073-256 |
| MIR30C1 | MK280073-263 |
| PEBP1 | MK280073-327 |
| TEAD2 | MK280073-392 |
| MIR302C | MK280073-393 |
| SMO | MK280073-394 |
| SDCBP | MK280073-400 |
| CREB1 | MK280073-401 |
| ELAVL1 | MK280073-408 |
| EPIST | MK280073-421 |
| PSMC4 | MK280073-422 |
| NEDD9 | MK280073-423 |
| MIR190A | MK280073-424 |
| MRE11 | MK280073-425 |
| CXCL1 | MK280073-426 |
| CCL20 | MK280073-429 |
| LGALS3 | MK280073-489 |
| SPOCK1 | MK280073-520 |
| HMMR | MK280073-539 |
| VDR | MK280073-576 |
| MIR92A2 | MK280073-587 |
| WNT7A | MK280073-599 |
| ITGB3 | MK280073-600 |
| TBX5 | MK280073-601 |
| MIR124-3 | MK280073-602 |
| CDH6 | MK280073-603 |
| LRP6 | MK280073-604 |
| RBX1 | MK280073-605 |
| MIR454 | MK280073-607 |
| AATBC | MK280073-608 |
| RUNX1 | MK280073-610 |
| ALDH1A1 | MK280073-611 |
| NEK2 | MK280073-612 |
| ESRRA | MK280073-613 |
| USP37 | MK280073-614 |
| FALEC | MK280073-616 |
| MIR153-1 | MK280073-617 |
| MIR191 | MK280073-619 |
| MIR449A | MK280073-620 |
| LINC-PINT | MK280073-622 |
| MCM5 | MK280073-626 |
| RBL2 | MK280073-627 |
| MUTYH | MK280073-628 |
| VEGFC | MK280073-630 |
| YY1 | MK280073-631 |
| SCHLAP1 | MK280073-632 |
| KAT5 | MK280073-633 |
| SPG7 | MK280073-634 |
| PIK3CB | MK280073-635 |
| PKD1 | MK280073-636 |
| PROX1 | MK280073-637 |
| FGF1 | MK280073-638 |
| CHUK | MK280073-639 |
| AGO2 | MK280073-640 |
| GAPDH | MK280073-641 |
| EDN1 | MK280073-642 |
| LOX | MK280073-643 |
| FGFR4 | MK280073-644 |
| DCLK1 | MK280073-645 |
| MIR342 | MK280073-646 |
| YWHAG | MK280073-647 |
| AGR2 | MK280073-648 |
| MIR24-1 | MK280073-649 |
| TP73 | MK280073-650 |
| HNF1A | MK280073-651 |
| MIR138-1 | MK280073-652 |
| DLG5 | MK280073-653 |
| MIR331 | MK280073-657 |
| ACTA2 | MK280073-658 |
| CUL3 | MK280073-660 |
| GATA4 | MK280073-661 |
| MECP2 | MK280073-662 |
| TDGF1 | MK280073-663 |
| MIR675 | MK280073-664 |
| CCDC144NL-AS1 | MK280073-665 |
| CASP8 | MK280073-666 |
| RIOK2 | MK280073-667 |
| AURKB | MK280073-668 |
| NR1D2 | MK280073-669 |
| KHDRBS1 | MK280073-671 |
| MIR128-1 | MK280073-672 |
| SPHK1 | MK280073-673 |
| MIR30D | MK280073-675 |
| SUMO1P3 | MK280073-677 |
| AXIN1 | MK280073-679 |
| MCAM | MK280073-680 |
| F2R | MK280073-681 |
| IL6R | MK280073-682 |
| NRP2 | MK280073-684 |
| CGB3 | MK280073-685 |
| SNHG20 | MK280073-687 |
| FAT1 | MK280073-690 |
| PRMT1 | MK280073-692 |
| PTK6 | MK280073-693 |
| LUCAT1 | MK280073-694 |
| PPP2R2A | MK280073-695 |
| RRM2 | MK280073-696 |
| PIK3R3 | MK280073-697 |
| MIR519D | MK280073-698 |
| BACH1 | MK280073-699 |
| HMOX1 | MK280073-700 |
| MIR194-1 | MK280073-701 |
| KRT19P3 | MK280073-702 |
| DAPK1 | MK280073-703 |
| IFNG | MK280073-704 |
| LYN | MK280073-705 |
| MIR135B | MK280073-706 |
| CDX2 | MK280073-707 |
| STUB1 | MK280073-708 |
| TCF4 | MK280073-709 |
| CRK | MK280073-710 |
| MIR378A | MK280073-711 |
| TRAF6 | MK280073-712 |
| MIR338 | MK280073-713 |
| FZD2 | MK280073-714 |
| MMP3 | MK280073-715 |
| TNFSF10 | MK280073-716 |
| PRKDC | MK280073-717 |
| MIR490 | MK280073-718 |
| PSMD1 | MK280073-719 |
| CDC6 | MK280073-720 |
| MECOM | MK280073-721 |
| EED | MK280073-722 |
| MIR590 | MYH7 |
| ANXA1 | LACTB |
| NT5E | PGM3 |
| FAM83D | RARS1 |
| FASN | PDSS2 |
| WNT2 | HSPA5 |
| CCL18 | ELAVL1 |
| CUX1 | MBOAT7 |
| PDGFD | HSPB1 |
| HOXA11 | TK1 |
| CXADR | CYC1 |
| HCCAT5 | MT-TI |
| CIP2A | ELN |
| B3GALT5-AS1 | SLC16A3 |
| USF3 | BCAT1 |
| PPP2CB | GAST |
| ANG | TUBA1A |
| SPRED2 | BSG |
| FERMT1 | ANPEP |
| ITGB4 | SETX |
| ENG | DELEC1 |
| ANO1 | SUCLG2 |
| MYBL2 | LARS2 |
| CTSL | HSD17B7 |
| RACK1 | IMMT |
| FOXP3 | ALDH1A3 |
| HOTAIRM1 | MYH9 |
| PRNCR1 | CTPS1 |
| SOX10 | GDF15 |
| S100A9 | SDHC |
| SHC1 | MRPL44 |
| CDH11 | SRC |
| RMST | GSK3A |
| IL4 | COQ4 |
| S100A8 | KRT18 |
| CSNK2A1 | MIR107 |
| LINC01186 | GANAB |
| IKBKB | SIRT7 |
| COL4A1 | XBP1 |
| PAX6 | MIRLET7A2 |
| ROR1 | MAF |
| TIMP2 | HCCAT5 |
| IRS1 | MIR128-2 |
| MIR135A1 | MIR16-1 |
| PDGFA | SLC27A2 |
| MIR483 | DBI |
| CTNNBIP1 | IGFBP2 |
| BTG2 | ATP5PO |
| MST1R | EIF2AK4 |
| DDX3X | ACAA1 |
| NSD2 | PCDH19 |
| PRKAA1 | RPS6KB1 |
| PXN | MT-TE |
| DLX6-AS1 | ZFAS1 |
| PSMC1 | MYD88 |
| JAK1 | MIR373 |
| PARD3 | NFS1 |
| MIRLET7I | ACE2 |
| SLC2A1 | RPS27A |
| CDH16 | SLC20A2 |
| SMARCA2 | NCOR2 |
| SRA1 | NIT2 |
| ROR2 | SIRT5 |
| YWHAE | TREX1 |
| KIFC1 | CALR |
| NTRK1 | RPE |
| MIR92A1 | GFPT2 |
| ERG | PPP1CC |
| PSMC2 | MIRLET7F2 |
| MIR487B | MIR124-2 |
| NFKBIA | PFKFB4 |
| DDX5 | SCARB2 |
| NTS | ATP1A1 |
| MIR515-1 | NDUFV2 |
| HEY2 | GRIN1 |
| MCC | YWHAG |
| MIR181A2 | PARK7 |
| PLK4 | VAPB |
| COL1A1 | THORLNC |
| UBE2T | DUT |
| DKK1 | DANCR |
| ATR | AMH |
| HIF1A-AS1 | TLR2 |
| TSC1 | CREB1 |
| STAT5A | DMD |
| ROCK2 | MIR215 |
| DGCR5 | SF3B1 |
| ENO1 | IARS1 |
| TET1 | ATP5F1B |
| DLEU7-AS1 | APOC4-APOC2 |
| MIR330 | TARDBP |
| VTCN1 | MIR205 |
| IL10 | TXNRD1 |
| SNHG17 | PTHLH |
| PIN1 | PDGFRB |
| ARF6 | ST3GAL3 |
| CDKN1C | SAMHD1 |
| MIR181D | DDOST |
| XIAP | FOXA1 |
| SOCS1 | TXNRD2 |
| ARID1B | SPHK1 |
| TUBA1B | SPTAN1 |
| AJUBA | RAB7A |
| MIR328 | UCA1 |
| NFATC1 | NOTCH2 |
| CCNG1 | MCU |
| MIR199A2 | IL1RN |
| ITGA6 | ACAA2 |
| KRT13 | MOGS |
| TRE-TTC3-1 | NOX4 |
| RPA1 | MIR125B2 |
| CALR | IL4 |
| FBXL19-AS1 | CYP51A1 |
| STIM1 | SPHK2 |
| MIR133A1 | HGF |
| PSMB8 | FAM210B |
| HEY1 | LOX |
| ENSG00000285988 | UBA1 |
| ZNF496-DT | HLA-A |
| SKIL | LRRK2 |
| SMURF1 | HYOU1 |
| SOX2-OT | TFEB |
| NCOA3 | MECOM |
| FMNL2 | TSC1 |
| PRKN | CDKN1A |
| TNFSF11 | MAPK3 |
| BAG3 | KAT5 |
| LINC00887 | PPP1CB |
| TERC | MAGED2 |
| NDRG2 | OSBP |
| NF1 | AK4 |
| FZD4 | ANXA5 |
| SMARCC1 | TGFBR1 |
| LASP1 | ELOVL2 |
| DLC1 | PMPCA |
| PFN2 | WRAP53 |
| IQGAP1 | SFXN1 |
| MIR184 | GNAI2 |
| DDX17 | CCN2 |
| NODAL | PCAT2 |
| PLOD2 | CASC19 |
| KIF11 | PRKCA |
| DEPDC1 | CASP3 |
| HDGF | FGF2 |
| PPP2R1A | FLNA |
| UBE2C | ACTA1 |
| LINC00941 | DPM1 |
| PLA2G4A | ERBB2 |
| SOD1 | RDH12 |
| DSP | TFAP2A |
| MIR518B | GGH |
| CTSB | PWAR1 |
| MIR125B2 | SNHG12 |
| PSMA3 | PDHA2 |
| CEP162 | NBN |
| HK2 | RHOA |
| HDAC9 | ESRRB |
| SEPTIN9 | GATA1 |
| GSN | SRA1 |
| TPX2 | NUP107 |
| PDGFRB | PDE3B |
| C11orf65 | NAXE |
| KDM6A | CDK4 |
| PSMD3 | CLPX |
| CYP19A1 | MT-TF |
| MIR153-2 | KDM1A |
| TRPS1 | NAT8L |
| CENPE | ATP6V0A2 |
| SOCS3 | CDA |
| LAMC2 | SOX9 |
| ERCC1 | HOTAIR |
| TACSTD2 | PRKAR2B |
| FERMT2 | PFKFB2 |
| MIR372 | ITGB1 |
| PSMD5 | STT3A |
| NRG1 | ACSL3 |
| NOS3 | MIR494 |
| ESPL1 | HLA-G |
| RHOC | CEL |
| PCAT6 | MGST3 |
| PDPK1 | HDAC4 |
| IHH | CLU |
| DLGAP1-AS1 | ITLN1 |
| CCL21 | MIR206 |
| MIR491 | CALM1 |
| PADI4 | IMPDH1 |
| TIMP3 | TRIM71 |
| LAMA3 | ABCB6 |
| CXCL5 | MIR10B |
| RAB25 | ENPP2 |
| NLRP1 | CSF3 |
| HSPA4 | ABCA4 |
| BARD1 | UQCRH |
| FLOT1 | NPPB |
| ALX1 | TWIST1 |
| ITGA3 | BAX |
| USP22 | TBP |
| FOXF2 | MARS1 |
| NKILA | MACROH2A1 |
| CRKL | HOXA11-AS |
| ZBTB7A | SLC6A14 |
| TUSC3 | P4HB |
| PMS2 | PTBP1 |
| TFAP4 | ATP5MK |
| SPINK1 | SSR4 |
| MIR340 | MIR19A |
| MIR663B | FBXW7 |
| MIR532 | CES2 |
| ALB | KDM6A |
| MIR155HG | MBTPS1 |
| BMPR1A | AURKA |
| NOX4 | NPM1 |
| USP7 | IGF2-AS |
| ESRP2 | PROX1 |
| SMAD1 | ACSS2 |
| SMC1A | TTN |
| STMN1 | CDK1 |
| PGK1 | LOC654780 |
| EMP2 | HNRNPU |
| KLK4 | FN1 |
| TBX1 | HTR2A |
| SPRY1 | GATA4 |
| GPC3 | NAGK |
| FGF7 | ZNRF3 |
| BCL9L | CEBPB |
| MAP3K7 | MIR369 |
| OSM | CTSB |
| TXN | IKBKB |
| PTPN12 | ATP6V1A |
| NQO1 | MIR181B1 |
| SPAAR | TP63 |
| MIR551A | HDAC3 |
| MIR212 | CDC42 |
| AGTR1 | RNU6-1 |
| HOXA10 | GNAO1 |
| UPF1 | HSD17B13 |
| GFRA1 | TCIRG1 |
| PHF8 | SMAD2 |
| LINC00665 | LOC106780800 |
| BTBD7 | EIF2AK1 |
| SRF | MIR320A |
| MIR361 | SPINK1 |
| ICAM1 | RNU6-2 |
| SLCO4A1-AS1 | RNU6-9 |
| SFRP2 | TPP1 |
| CPS1-IT1 | ALDOC |
| CEBPB | DGCR5 |
| IDO1 | PIK3R2 |
| PTP4A3 | MRPS34 |
| WNT2B | DPEP1 |
| STC2 | NCOR1 |
| CTDSP1 | MT-TH |
| CEACAM1 | SLC22A1 |
| NOS2 | SDC1 |
| CTHRC1 | AARS2 |
| IGF2BP1 | MIR9-2 |
| KMT2C | M6PR |
| CSF2 | RAD51 |
| MIR124-2 | AP1S1 |
| TIMP1 | CLN3 |
| CSF3 | TGM2 |
| AIFM1 | YWHAZ |
| CXCR2 | HSD17B12 |
| DPP4 | ELANE |
| CXCL10 | CYLD |
| MCM7 | NDUFB8 |
| GLI2 | TLR3 |
| SALL4 | VIM |
| NTRK3 | SNORD118 |
| MIR181B1 | MGP |
| SYP | CSF1 |
| KIF20B | LTF |
| XRCC1 | RNU6-7 |
| ALDOA | RNU6-8 |
| EPHA3 | RNU6-1-001 |
| PRKCD | RNU6-1-002 |
| HSF1 | RNU6-1-003 |
| KMT5A | RNU6-1-004 |
| SETDB1 | MIR216A |
| MIR339 | PTGES |
| FZD5 | AKT3 |
| IL1A | EHMT2 |
| DCN | NOTCH1 |
| CLDN4 | COPG2IT1 |
| MCL1 | UGDH |
| MT-TL1 | ERN1 |
| ECM1 | PKD2 |
| LRG1 | ECI1 |
| SLC25A5 | NCOA3 |
| PRSS8 | SESN2 |
| TRIM24 | ACSF2 |
| AREG | CYBA |
| FGF8 | NDUFS6 |
| MIR1271 | IL15 |
| NUAK1 | SMCHD1 |
| HNRNPK | TOP2A |
| MIR34B | TIMM50 |
| LTBP1 | VLDLR-AS1 |
| CXCR3 | YWHAQ |
| FLOT2 | HSF1 |
| H2AX | NDUFA2 |
| MIR9-3 | E2F1 |
| DLEU2 | CDH1 |
| WWOX | MEG8 |
| SFTA3 | GPC3 |
| SNORA66 | SNHG6 |
| RASAL2 | ACOT12 |
| LRP5 | NSD2 |
| DNM1L | SMAD4 |
| SERPINF1 | CD274 |
| C3 | PNKP |
| UBA52 | PRKCZ |
| PDCD1 | GLUD2 |
| OLR1 | PPA1 |
| ANPEP | CA9 |
| EPB41L3 | SELENBP1 |
| CCR2 | FLNC |
| CFL1 | IL4I1 |
| SETD2 | ERCC3 |
| KAT2B | ARID2 |
| CERNA2 | PAX2 |
| USP47 | DCTN1 |
| MIR100HG | SOCS3 |
| TGIF1 | PAPPA-AS1 |
| HAS2 | NCOA1 |
| ATF3 | MIR224 |
| MIR33A | NFKBIA |
| MIR95 | AGPS |
| STAT5B | WT1 |
| BACE1-AS | DIO3 |
| MIR7-3 | RAC1 |
| ATL1 | MLH1 |
| TFF1 | ITGAM |
| HOXA13 | MAGT1 |
| GRN | SEMA4D |
| MIR19B1 | DDIT3 |
| FER1L4 | DDX5 |
| WNT7B | PTGES2 |
| JARID2 | SOD3 |
| ELF5 | APEX1 |
| VASH2 | BICD2 |
| S100P | NMNAT1 |
| HNRNPA1 | YWHAB |
| SND1 | MAEL |
| VHL | FLG |
| TNFSF15 | LMNB1 |
| MIR301B | PRPSAP1 |
| EGOT | MLX |
| TCF21 | NDUFA11 |
| ACKR3 | AGO2 |
| SLC25A6 | YY1 |
| VCL | ACSL5 |
| MIF | LARS1 |
| DLEU1 | ENO2 |
| PSMD10 | PLIN3 |
| MIR196A1 | LINC01139 |
| MIR138-2 | IDH3B |
| IL7 | IKBKG |
| SMARCD3 | CERK |
| PSMD2 | COL4A5 |
| SPI1 | KCNJ8 |
| PAX8 | CYTOR |
| ETS2 | GLRX5 |
| NKX2-1 | MIR24-2 |
| PRKCI | HDAC2 |
| PAX2 | NBR2 |
| FH | GPC4 |
| TMSB4X | DDX3X |
| SPTBN1 | MIR211 |
| FANCI | STUB1 |
| TRPM7 | APLN |
| MIR218-1 | EEF2 |
| MIR30C2 | IGFBP4 |
| CENPF | SRR |
| MIR152 | PABPC1 |
| OLFM4 | RAD50 |
| OVCH1-AS1 | HNRNPC |
| MIR7-1 | TGFBR2 |
| ANXA5 | PAX6 |
| S100A7 | H3-3A |
| TRERNA1 | ATP6AP1 |
| SST | DDAH1 |
| LINP1 | MRPL39 |
| SYK | H3C1 |
| MIR638 | MIPEP |
| MIR455 | CUL4B |
| IGF2BP3 | DDAH2 |
| SERPINB3 | FDX2 |
| KRT5 | ADHFE1 |
| EPHA4 | DHPS |
| PSMD14 | LINC00092 |
| AHR | MIR1291 |
| NOX1 | ADAR |
| MIR363 | CYBRD1 |
| UBE2D3 | TUBB6 |
| HDAC5 | FA2H |
| RECK | PPP2R1A |
| ERP29 | GUK1 |
| TUSC8 | XPC |
| NBR2 | BECN1 |
| ADAR | NME1 |
| FOXK1 | TIMM8A |
| GDNF | SHC1 |
| PGF | LSS |
| SEMA3C | RIPK1 |
| MIR377 | SMN1 |
| SERPINA3 | HMGB1 |
| MIR187 | NF1 |
| MUC2 | ARAF |
| TSC2 | AK3 |
| MIR501 | GNB1 |
| HOXD9 | XRCC3 |
| MEG8 | GARS1 |
| ITCH | KLF11 |
| DLK1 | HDAC9 |
| TMPO-AS1 | MVD |
| SOS1 | SLC1A1 |
| RICTOR | NNT |
| HPSE | CNP |
| NGF | CEACAM5 |
| COPS5 | AADAT |
| IRS2 | SPTBN1 |
| MCM3AP-AS1 | NDUFB3 |
| CEP164 | HNRNPD |
| DHFR | ACTN3 |
| MIR574 | TRV-AAC1-1 |
| MIR520G | TRV-AAC1-3 |
| CUL2 | TRV-AAC1-4 |
| KDM5B | TRV-AAC1-2 |
| MCM3 | TRV-AAC1-5 |
| HIC1 | DNM2 |
| FOXD3 | NDUFAF3 |
| EDNRA | EEF1A1 |
| SKI | UQCRC1 |
| LINC01116 | TRIB1 |
| TRIP13 | MGST1 |
| KLF8 | SLC25A5 |
| LGR4 | SATB1 |
| MIR615 | TOMM40 |
| GDF9 | DICER1 |
| MIR217 | TNFAIP3 |
| KDM4B | SLC2A5 |
| PTPN13 | NDUFB7 |
| GPER1 | RRM2 |
| PDK1 | SNX10 |
| NR2F1-AS1 | IARS2 |
| KDM6B | PSEN2 |
| CCN5 | MROCKI |
| ERLNC1 | GNB2 |
| DNMT3A | UPP1 |
| IL17RD | ALDH16A1 |
| MIR1236 | H4C1 |
| CD276 | OGA |
| MIR496 | TCF3 |
| TBL1XR1 | CD47 |
| NHERF1 | LAMP1 |
| SOX21-AS1 | NCDN |
| HOXB7 | MYCN |
| MIR551B | STING1 |
| SENP1 | FALEC |
| MIR486-2 | TRIB3 |
| MIR371A | PRKCSH |
| MIR410 | TBC1D24 |
| TRP-AGG2-5 | NDUFA8 |
| TRP-AGG2-6 | SELENOI |
| TRP-AGG2-1 | POLR3H |
| TRP-AGG2-2 | HHEX |
| TRP-AGG2-3 | CTBP1 |
| TRP-AGG2-4 | ME2 |
| TRP-AGG2-7 | LIF |
| TRP-AGG2-8 | PLCG2 |
| MBD3 | PLD1 |
| OPRM1 | ALPG |
| PBK | CSNK2A1 |
| LINC00922 | NUP93 |
| XRCC5 | TRAP1 |
| SOS2 | SIN3A |
| HPGD | IL17A |
| TNFRSF19 | NDUFAB1 |
| MUC4 | MIR186 |
| TAGLN | GRIN2A |
| MIR107 | RIOX2 |
| MIR197 | CDK5 |
| ZNF217 | RANBP2 |
| MYH9 | ASCL1 |
| MIR3622A | GABRA1 |
| CASC11 | PRDX6 |
| RNF8 | SELL |
| F11R | ENSG00000276609 |
| HSP90B1 | ENSG00000277469 |
| TET3 | ENSG00000277553 |
| WDR5 | ENSG00000278708 |
| OGT | KDR |
| PRKCB | UGT2B28 |
| HSPA9 | FILNC1 |
| DLX4 | ENSG00000232995 |
| IL15 | METTL16 |
| ATF2 | CASC2 |
| NPM1 | GFAP |
| LINC00339 | CCAT2 |
| KRT14 | CXCR4 |
| ELL3 | GJA1 |
| MIR876 | MIR96 |
| PTK2B | MT-TA |
| BAP1 | PML |
| TRIM28 | LGALS3 |
| FANCD2 | RPS6 |
| MVP | MUC4 |
| PRDX1 | METTL3 |
| TGFBR3 | PRKDC |
| SPRY4 | ONECUT1 |
| EOMES | RAF1 |
| CYP1B1 | NKX2-5 |
| NR5A2 | MIR103A2 |
| LMNA | UBE3A |
| LAMP1 | ITGB2 |
| SDHB | TLX1NB |
| PCAT7 | COX6C |
| PIAS3 | DDX6 |
| RCC2-AS1 | GATA2 |
| ARID2 | PTGES3 |
| FUT4 | TRPV4 |
| EDN3 | KDM5C |
| SIRT3 | TUBB |
| BLM | EGR1 |
| USP9X | IRF4 |
| USP2 | CDK2 |
| RGS6 | FMR1 |
| LINC02446 | FOXK2 |
| DYRK2 | MIR1-2 |
| LINC00467 | KCNJ2 |
| HOOK1 | MDM2 |
| TRIM62 | LAMTOR5 |
| TNFRSF11A | PDCD1 |
| MEF2D | CANX |
| MIR299 | DKC1 |
| BHLHE40 | LGALS1 |
| HDAC8 | STC1 |
| KIF4A | MBOAT2 |
| BCAR1 | PLPP3 |
| FZD8 | NME2 |
| GH1 | MATR3 |
| LINC00960 | SATB2 |
| GAPLINC | SOX9-AS1 |
| PTP4A1 | CACNA1G-AS1 |
| ASCL2 | CD8A |
| ENSG00000285959 | BTK |
| RTN4 | PRKD1 |
| MAP2K4 | ROCR |
| MSH3 | ENSG00000288605 |
| TP53INP1 | LOC102723517 |
| INHBA | HOXA13 |
| MIR423 | S100A8 |
| RHOD | PRPS2 |
| PKD2 | MIR485 |
| EFEMP1 | SERPING1 |
| SLC25A3 | TRIM28 |
| AMFR | MIR375 |
| GPI | FDPS |
| LINC00313 | PTK2B |
| PTPN1 | IL3 |
| LINC00857 | COL7A1 |
| GLIPR2 | MIR181D |
| IL11 | SIK2 |
| FGF10 | PYURF |
| ERAS | MFN1 |
| DEK | NPTN-IT1 |
| RCOR1 | YME1L1 |
| CLDN17 | KMT2D |
| F2 | FARSA |
| PPARGC1A | ZFP42 |
| HES1 | EIF4G1 |
| MMP13 | CXCL12 |
| UBD | ACTG1 |
| GAS1RR | SLC43A1 |
| MIR539 | CALU |
| RDX | CCL5 |
| IL37 | HNRNPF |
| LINC01139 | ADSS2 |
| CSTF2 | CLCN7 |
| CALM1 | ATP5IF1 |
| LDHA | ATF6 |
| S100A2 | YTHDF2 |
| LIN28A | TTF2 |
| CHKA | SNHG3 |
| UBE2I | ARX |
| CD47 | MIR370 |
| GCNT2 | COX7C |
| MIR301A | HACD3 |
| SLC39A6 | MIRLET7F1 |
| MIR219A1 | IL2RG |
| CKS2 | ACIN1 |
| NKX6-1 | CDC73 |
| MIR367 | FLCN |
| PRKAR2B | MIR124-3 |
| MAEL | RBPJ |
| KRT1 | LRP6 |
| B2M | PHB1 |
| UBE2E2 | PPP2CA |
| TUBB3 | GPX3 |
| RHOB | PLP1 |
| LRIG1 | SMARCAL1 |
| ENSG00000271204 | KRT14 |
| MCM6 | GRB2 |
| TP53BP2 | TMEM165 |
| FZD10 | IL4R |
| H4C16 | TBK1 |
| AFP | DHX9 |
| MCRS1 | PDIA3 |
| SNHG3 | QPRT |
| DCST1-AS1 | OLR1 |
| PLS3 | ACTN1 |
| EHMT2 | PCBP2 |
| CHI3L1 | MIR202 |
| BTF3 | SHH |
| CENPW | SLC1A2 |
| HHIP-AS1 | KPNA2 |
| PRDX5 | CEMIP |
| NEDD1 | FUT4 |
| MROCKI | MIR181B2 |
| SLIT2 | PTK2 |
| TNNC1 | AARS1 |
| MYOSLID | POLR1C |
| SIRT6 | RNU1-1 |
| KDM2A | PRKCD |
| PITPNA-AS1 | DEGS1 |
| MIR382 | JMJD8 |
| YTHDF2 | OSBPL9 |
| SGK2 | DDR1 |
| MIR489 | RNU1-4 |
| PTENP1 | RNVU1-18 |
| TNFAIP8L2 | RNU1-2 |
| GDF10 | RNU1-3 |
| MIR1291 | RNVU1-29 |
| CDKL2 | LOC124904613 |
| CA2 | SH2B1 |
| LINC01638 | NR4A2 |
| STEAP1 | SLC38A2 |
| AKAP9 | CTLA4 |
| CEACAM6 | GPER1 |
| PRL | CASC8 |
| GAS6 | UCHL1 |
| TUNAR | EIF4E |
| HOXB13 | ACTN4 |
| MIR509-1 | TRPM6 |
| ST6GAL1 | MT-RNR2 |
| CATIP-AS1 | ENG |
| LINC01592 | SMN2 |
| DUXAP10 | PRDM16 |
| SERPINH1 | DNMT3L |
| COPB1 | ATP6V0A1 |
| DLX2 | TGFB2 |
| TYMP | FARSB |
| CD151 | HELLS |
| IL33 | FMOD |
| BIRC7 | BRD2 |
| ERCC4 | ACVR1 |
| NANOS3 | H4C16 |
| FZD6 | CARMN |
| ANGPT2 | DYNC1H1 |
| NUBPL | YWHAH |
| CTDSPL | FOSL1 |
| MYCN | ATRX |
| SH2B1 | TOP1 |
| RYR3 | BMP4 |
| MIR1294 | PRDX4 |
| VCAN | POLR2A |
| ANGPTL4 | PSMC4 |
| GATA2 | MIR511 |
| METTL13 | STAT5B |
| MIR885 | METTL23 |
| ADPGK-AS1 | IL13 |
| INSR | ARHGAP1 |
| FOXP2 | CMPK1 |
| KCNH2 | ZEB1 |
| SATB2 | SNHG14 |
| CTNNAL1 | CAV2 |
| PRPF4B | MITF |
| HAVCR2 | WBP11 |
| EDNRB | MBL2 |
| E2F5 | FEN1 |
| LIF | NORAD |
| ACVR1 | UBC |
| PRKCE | MIRLET7A3 |
| ADAM9 | MIR208B |
| FURIN | PRPF19 |
| RAB27A | EMC1 |
| CLDN23 | KISS1 |
| IGFBP3 | S100A9 |
| LINC01234 | FAS-AS1 |
| WNT5B | ALK |
| CLCA2 | USP7 |
| GLI3 | TRPM7 |
| SET | LINC01191 |
| FAM107A | DCAF7 |
| MIR1246 | STAT5A |
| LIMS1 | ABL1 |
| APOC1 | MIR26A2 |
| RCC2 | LIN28B |
| PML | CX3CR1 |
| TYR | CHI3L1 |
| NAMPT | SFN |
| FADD | IGF2BP1 |
| PTPA | CUL4A |
| FYN | FZR1 |
| RAB1A | CSF2 |
| GPC5 | THOP1 |
| TFAP2A | NSF |
| ITGB6 | SNAI1 |
| CDK14 | SLC23A2 |
| USP4 | PRDX3 |
| TPT1-AS1 | MTCH2 |
| LINC01191 | REST |
| EPB41L4A-DT | POMGNT2 |
| FASLG | CDK9 |
| CLDN11 | APOBEC1 |
| COMP | ENSG00000274430 |
| EHMT1 | ENSG00000276784 |
| HIPK2 | VAPA |
| AKIP1 | KLF5 |
| RPA2 | CAMTA1 |
| PDE4A | PRMT5 |
| CEACAM5 | STRA6 |
| IL18 | SEC24C |
| EREG | CXCL10 |
| SNORD43 | IRF1 |
| MAD2L1 | OGG1 |
| NEDD4 | GNPNAT1 |
| LINC01587 | SELENOP |
| FHL2 | BAZ1B |
| NGFR | MT3 |
| HPRT1 | NR4A1 |
| ERN1 | HAGLR |
| DACH1 | MIR519D |
| CDK5RAP3 | WARS1 |
| BCL9 | LINC00926 |
| NTN1 | KRT8 |
| CKB | MBP |
| ZFYVE9 | CHRM3 |
| LYPD3 | KMT2C |
| FLT1 | STIM1 |
| PTN | OSM |
| KMT2D | RHO |
| HOXB-AS3 | TRAF6 |
| ADIPOR1 | HMGCLL1 |
| ENSG00000232995 | SETSIP |
| MME | NUCB2 |
| PTBP1 | SPARC |
| KLK6 | TAOK1 |
| HSP90AB1 | VDAC3 |
| CCN4 | MPC2 |
| FOXP1 | SOX2-OT |
| NNMT | SVIL |
| CEP55 | VPS35 |
| ACTA1 | RNU4ATAC |
| FABP5 | HBEGF |
| FPR2 | NUDT5 |
| MIR874 | CD59 |
| SIRT7 | ADPGK |
| TM4SF1 | TP53COR1 |
| PXDN | IGF2BP3 |
| ENSG00000269966 | TRAF3 |
| SREBF2 | VCL |
| KLF2 | CCR5 |
| MIRLET7E | CDK6 |
| ADIPOQ | P2RX7 |
| ARHGDIB | SFPQ |
| MIR425 | ATP1B1 |
| IL32 | PCNA |
| CFAP251 | CDX2 |
| SIM2 | EGLN1 |
| EIF3E | SEC23B |
| MEN1 | SUMF2 |
| MIR431 | PF4 |
| CRABP2 | IATPR |
| SFN | EIF4B |
| SLC9A1 | DEK |
| VWCE | WNT5A |
| LINC01315 | MSH2 |
| MIR24-2 | WWTR1 |
| PLAGL2 | DAXX |
| EWSAT1 | MIR137 |
| MIR509-3 | VTRNA1-1 |
| PRKAA2 | CFL1 |
| MILIP | ANXA2 |
| TAFAZZIN | TUBB2A |
| S100A11 | BMP7 |
| PBXIP1 | MYOD1 |
| NMRAL2P | NOTCH3 |
| POLE2 | DLEU1 |
| AHNAK | PDPK1 |
| ROBO1 | HAS2 |
| EYA2 | GSTK1 |
| MED28 | EMD |
| TRAP1 | MIR152 |
| PSMC3 | NUP133 |
| JAG2 | TST |
| GATA1 | ATF3 |
| CEBPA | POU1F1 |
| PRKCZ | DHRS2 |
| TUFM | RICTOR |
| POU2F1 | IL1R1 |
| CDT1 | ADAM10 |
| ABCC1 | HSP90AB1 |
| SNORD24 | GLI3 |
| F3 | KMT2A |
| FFAR4 | RPN1 |
| PSMC5 | HNRNPM |
| NCAM1 | TET1 |
| GMPS | ESRRG |
| FNDC5 | CA4 |
| WNT11 | PAX5 |
| MIR196A2 | ATP6V1E1 |
| MIR300 | MIR379 |
| KISS1 | COX6A1 |
| MAP2K3 | ERBB4 |
| RNY5 | FLT4 |
| CLCA4 | PLD2 |
| MAPKAP1 | COX5B |
| RAB43 | MAPK9 |
| TLN1 | MIR181C |
| CHGA | ETS1 |
| CCL15-CCL14 | YBX1 |
| MMP15 | MIR7-1 |
| CDH5 | TOMM20 |
| TBK1 | PIN1 |
| EEF2 | HDLBP |
| DPP10-AS1 | MEF2C |
| LZTS1 | SNORD44 |
| FTL | ADAM17 |
| HNRNPM | DKK1 |
| MSI2 | SEC61A1 |
| MIR362 | VDAC2 |
| SNHG4 | ROCK1 |
| WNT10B | CACNA1C |
| ALKBH5 | IKZF1 |
| TUBB | ILF3 |
| MIR644A | EIF4A1 |
| SASH1 | ITPR2 |
| MYO5A | BMPR2 |
| GDF5 | UBXN1 |
| BAD | JAG1 |
| S100A16 | PSMB4 |
| MIR4458 | CD34 |
| IL13 | OSBPL1A |
| HOXB9 | SALL4 |
| TRD-GTC9-1 | TIMP3 |
| MIR20B | BIRC3 |
| UBE3C | AUP1 |
| MAGEC2 | CBX5 |
| KCNN4 | COX7A2L |
| GOLPH3 | TMEM30A |
| ACP1 | EWSR1 |
| MIR503HG | ATP6AP2 |
| MIR1269A | COX17 |
| FGD5-AS1 | TGFBI |
| FBXO31 | SPI1 |
| AQP3 | GTPBP3 |
| CCR6 | HSPA6 |
| FOXJ2 | TBX1 |
| FOXS1 | XRCC6 |
| FOXR2 | ATP6V1B2 |
| MIR613 | PTCHD1-AS |
| FBXW11 | IRAK1 |
| TMOD3 | ERLIN2 |
| SOX3 | ATP1A3 |
| PBRM1 | BANCR |
| ENSG00000271590 | IL5 |
| ENSG00000285016 | MIR106A |
| WNT3 | OXTR |
| P4HA3 | ENSG00000250362 |
| LHPP | ENSG00000272021 |
| LEFTY2 | LOC102724720 |
| CSK | GPS2 |
| ZBTB33 | SARS1 |
| EHD1 | DOLK |
| SLC16A1 | KRT19 |
| PFKFB3 | BLM |
| UBE2S | KPNB1 |
| ING4 | OVCH1-AS1 |
| IGFBP2 | NNT-AS1 |
| GKN2 | KIF1A |
| ROR1-AS1 | ID2 |
| MIR2392 | GABRE |
| NIHCOLE | UBE2I |
| MAX | NUP98 |
| GIT1 | ATP5PD |
| GGCT | SNHG5 |
| ACTN4 | KDM6B |
| MIR1290 | ELP1 |
| AKAP8 | NCAPH2 |
| KMT2A | PPP2R1B |
| CLDN5 | GNG5 |
| MIR630 | CBR3-AS1 |
| USP11 | PAK1 |
| ZNF667-AS1 | MFGE8 |
| MITF | PGRMC1 |
| CEP192 | IL33 |
| E2F8 | BIRC5 |
| SLX1A-SULT1A3 | MIR302D |
| RIPK4 | HCN2 |
| NKD2 | DRD3 |
| SNW1 | CCT2 |
| AFG3L2 | MYT1L |
| AICDA | MPST |
| ATG7 | TUBB3 |
| SSB | TLR8 |
| RARB | SEC13 |
| TUFT1 | RARG |
| PHACTR2-AS1 | SKP2 |
| MIR205HG | ZEB2-AS1 |
| COL11A1 | P2RX5-TAX1BP3 |
| COL4A2 | HNRNPH1 |
| LINC02231 | MIR148B |
| TPM1 | LINC00941 |
| MIR369 | RPL18 |
| ACTG1 | NOD2 |
| OCLN | METTL14 |
| VANGL1 | INHBA |
| NUCB2 | EIF2AK2 |
| CHPF | ATP5F1C |
| CCN6 | RTN4 |
| CGAS | THPO |
| TRIM16 | ERBB3 |
| RALA | MIR22HG |
| ELK1 | CGB5 |
| PEAK1 | STT3B |
| FSHR | DOT1L |
| USP17L2 | CD14 |
| WDR77 | RNF217-AS1 |
| TK1 | CDH2 |
| MNX1 | JAK3 |
| MIR577 | FOXP1 |
| MIR3127 | RPN2 |
| MIR203B | CHUK |
| CLDN14-AS1 | PRDM14 |
| NBAT1 | NCL |
| AMH | ENSG00000274760 |
| LINC01567 | ENSG00000277967 |
| SDC2 | CTCF |
| RUFY3 | MST1R |
| CXCL9 | TARS1 |
| PDX1 | PSMD2 |
| ISL1 | H2BC21 |
| RELB | H4C5 |
| S100A1 | SNHG16 |
| FBP1 | LETM1 |
| IGF2-AS | RFC1 |
| LINC00668 | KHSRP |
| MTA3 | KHDRBS1 |
| MFN1 | CCNE1 |
| IRF1 | CDKN1C |
| MIR421 | NDRG1 |
| TET2 | NR2F2 |
| RAD50 | PABPC4 |
| AIM2 | LAMTOR2 |
| AKR1B1 | BRD4 |
| ARF1 | HSPA9 |
| MCM4 | PRECSIT |
| RFC3 | ANXA6 |
| ADRB2 | ANXA1 |
| H3C1 | ATP5PB |
| TRN-GTT2-1 | SLC7A8 |
| TRN-GTT2-5 | LINC00538 |
| TRN-GTT2-6 | PWAR4 |
| TRN-GTT2-2 | HOXB-AS3 |
| TRN-GTT2-3 | PTPA |
| TRN-GTT2-4 | IQGAP1 |
| TRN-GTT2-7 | JUP |
| TRN-GTT2-8 | CCAR2 |
| DTL | NEDD4L |
| VLDLR-AS1 | FEZF1-AS1 |
| RBPJ | LINC00665 |
| NRF1 | XRN2 |
| UBE2V1 | VANGL1 |
| MIR151A | PAK2 |
| FEN1 | FLT1 |
| FOXG1 | TPT1 |
| S1PR1 | MSTO1 |
| UBAP2L | GNB4 |
| TRIM21 | SLC38A3 |
| KTN1-AS1 | CAMKK2 |
| PODXL | GCN1 |
| UBA1 | TNFSF13B |
| NCOA1 | LINC00473 |
| BIRC2 | PCMT1 |
| PAX5 | CEACAM1 |
| CTH | CNTF |
| FGF19 | CARS2 |
| EEF2K | PLK1 |
| H2BC21 | TRD-GTC9-1 |
| PWAR6 | GSN |
| SMAD6 | NOP56 |
| MIR16-2 | ATP2A1 |
| DAXX | C11orf65 |
| LGR5 | XRCC5 |
| SLC2A3 | ULK1 |
| MIR526B | SYK |
| BHLHE41 | CCT7 |
| KRT23 | NCF1 |
| LOXL3 | BCAR4 |
| EIF4A3 | CSF1R |
| CTSZ | SMARCD3 |
| IKBKE | DBP |
| CD8A | PRMT1 |
| SFRP4 | CCT3 |
| ASCL1 | THEM4 |
| LINC01554 | IFNB1 |
| KITLG | BCR |
| RAB22A | ACOT9 |
| HSPA1A | MICB |
| MARVELD3 | LINC-PINT |
| MAOA | STEAP3 |
| UHRF2 | RNF2 |
| MIR99B | SSBP1 |
| PCA3 | EPHB4 |
| HOXA9 | MIR367 |
| GLIS2 | ENSG00000271204 |
| EEF1D | ACSS1 |
| SSTR5-AS1 | MAFA |
| COPS6 | MT2A |
| UIMC1 | RAN |
| IGFBP5 | TNFSF10 |
| TMEM238L | LOC110806263 |
| CDK11B | H2AZ1 |
| HIP1 | PRLR |
| MIR374C | MCL1 |
| DDX11-AS1 | THRAP3 |
| HNRNPAB | PSMD10 |
| VTN | DDB1 |
| CLDN2 | PIM1 |
| SCNN1A | SIL1 |
| RAMP2 | NUP155 |
| RAD9A | CKAP4 |
| RBBP5 | H4C15 |
| NR2F1 | EEF1G |
| MIR103A2 | PSMD12 |
| MIR589 | ZEB2 |
| STX2 | PTX3 |
| PIMREG | RIGI |
| MYCNOS | RAB5A |
| CDK11A | NPHS2 |
| HRNR | PMPCB |
| MAGI2-AS3 | NUP37 |
| MIR323A | TXN2 |
| LINC01705 | PDIA6 |
| DES | CYB5B |
| FERMT3 | PSMC1 |
| ABCC3 | NUP88 |
| PTPRA | RPSA |
| AEBP1 | RARB |
| LIMA1 | PITRM1 |
| EDA | NIPSNAP3B |
| MRTFB | PRPF8 |
| SDHAF2 | LHPP |
| CTAG1B | RPL13A |
| KLB | H4C14 |
| LONP1 | NR2E3 |
| ME1 | LNCARSR |
| PBX3 | PLEC |
| AGT | UQCC3 |
| PCBP1 | ESYT1 |
| TRIM29 | ACP2 |
| ULK2 | PLAUR |
| EGFL7 | MIR196A2 |
| SPRR2A | RAB6A |
| TMEM45A | PGLS |
| H2AC20 | MACROD1 |
| RNH1 | ME3 |
| MIRLET7A2 | SYNCRIP |
| MIRLET7A3 | NFATC1 |
| MGLL | AICDA |
| MIR136 | TBL2 |
| WASF3 | PRTN3 |
| MIR641 | RMST |
| G3BP1 | HOXB-AS1 |
| ATP6V0C | TMEM51-AS1 |
| MPC1 | PPT2-EGFL8 |
| SGK3 | HOTAIRM1 |
| LMCD1 | BABAM1 |
| PPP4R1 | MIR184 |
| LBH | POU3F2 |
| SPZ1 | PSMA6 |
| HTRA1 | PLOD2 |
| SERPINA1 | LINC00963 |
| TFDP3 | PLN |
| TEK | STOML2 |
| P4HB | PKP2 |
| DKK3 | SEC23A |
| JAKMIP2 | ETV2 |
| TOPAZ1 | MBD3 |
| AFAP1L2 | NONO |
| RHBDD1 | CASC15 |
| TRIM66 | CHD3 |
| CHD4 | VPS16 |
| LEF1-AS1 | PACSIN2 |
| ETV4 | RPL14 |
| PCMT1 | TUT7 |
| MIR511 | COPA |
| ITGBL1 | FHL2 |
| PCDHGA9 | DNA2 |
| BATF2 | H4C3 |
| MIER3 | AURKB |
| GATA2-AS1 | CCT6A |
| DLL1 | HCAR1 |
| PRNP | TP73 |
| KDM5A | SOS1 |
| PAQR3 | FLI1 |
| VTRNA1-1 | GIGYF2 |
| TMPRSS11A | RUNX1 |
| JPX | IDH3G |
| GRP | TFG |
| MTA2 | FBXO32 |
| STAT6 | MIR187 |
| TFRC | NF2 |
| LAMA5 | NDUFA12 |
| NCOR1 | CNOT1 |
| NMI | MIR1-1 |
| SLFN5 | EEF1D |
| USP3 | PSMC6 |
| MIR493 | KAT2B |
| MIR708 | ATP2A2 |
| AP2B1 | SFRP4 |
| ANXA2R-OT1 | SIN3B |
| LINC02154 | MIEF2 |
| LINC02599 | CHD7 |
| LINC02862 | RPL6 |
| UBE2V2-AS1 | CELSR2 |
| ENSG00000274430 | AGPAT4 |
| ENSG00000276784 | TUBB4B |
| LOC654780 | CSNK1A1 |
| TPBG | MIR99B |
| MGAT5 | ST6GAL1 |
| BMPR2 | IKBKE |
| PTPN6 | MMP14 |
| IQGAP3 | USP11 |
| CNP | NME7 |
| HTATIP2 | EPHA2 |
| GKN1 | H4C9 |
| BUB3 | LCK |
| CHKB-CPT1B | FABP7 |
| SOCS2 | TTLL4 |
| CDC73 | BCYRN1 |
| GASAL1 | EIF4A2 |
| FKBP1A | MAP1LC3B |
| MIR219A2 | MT4 |
| FAM3B | FIS1 |
| MEX3C | MLXIP |
| DEDD | ZFP91-CNTF |
| PCGEM1 | CXCR3 |
| MIR622 | LMAN1 |
| GFER | TAP1 |
| ZC3HAV1 | SKP1 |
| PPM1D | GNG12 |
| MIR650 | SLC16A7 |
| TUSC7 | H4C11 |
| AKR1C3 | H4C2 |
| ITGA4 | H4C8 |
| MAP2K5 | H4C12 |
| ERO1A | H4C4 |
| FXYD5 | H4C6 |
| LINC01614 | H4C13 |
| GNL3 | NSD1 |
| PSMA4 | PPP3CA |
| MIR194-2 | NDUFA5 |
| KRT17 | GTF2I |
| MMP19 | LAMTOR1 |
| BIRC6 | OLA1 |
| TES | C3AR1 |
| NFATC2 | CYB5R1 |
| SENP3 | ILK |
| ZFP36 | G3BP1 |
| FAM13A | SRSF2 |
| MIR18B | EIF3M |
| KRT9 | SMARCC1 |
| ESRRB | MCFD2 |
| PSMD6 | ASCC3 |
| HDLBP | CAMK2B |
| CAMK2G | PHF5A |
| ATP5F1B | KLHL25 |
| FLT4 | EIF3E |
| FTX | SEC61B |
| LINC00858 | AGMAT |
| RNF43 | COPS6 |
| MIR374A | RPL29 |
| MIR545 | CCT5 |
| TAF1 | SNRPN |
| GJB1 | ACOT13 |
| PSMD4 | DDX17 |
| LOXL1-AS1 | IFI27 |
| FSTL1 | FAF2 |
| ZNF750 | CCN1 |
| MIR302B | SELENOM |
| EPRS1 | UBE2D2 |
| C5AR1 | LYN |
| HSPB2 | CCL20 |
| HSPA8 | NFYA |
| GPX2 | MAP1LC3A |
| HEYL | MUC16 |
| PDLIM2 | KISS1R |
| MIR616 | ATP5MF |
| ZFHX3 | FABP5P3 |
| MIR105-1 | ATP6V0D1 |
| BRMS1 | RPL15 |
| CACNA1G-AS1 | EZR |
| PAK5 | SETDB1 |
| ITGA9 | FANCD2 |
| LINC01235 | MX1 |
| MIR4732 | PLIN4 |
| PPARA | NFE2L1 |
| SMARCC2 | MAPK7 |
| PAK4 | WT1-AS |
| MAPK10 | MUL1 |
| LINC01088 | PTGER2 |
| MIR572 | AAAS |
| RPTOR | NUDT19 |
| ACE2 | CASC9 |
| METTL14 | LINC01018 |
| FNDC3B | WDR5 |
| EIF4G1 | HIBADH |
| MIR877 | KRT7 |
| NR1I2 | RUVBL1 |
| TGM3 | SUMO1 |
| SERPINC1 | LINC00958 |
| DDX21 | SND1 |
| MIR661 | TRMT1 |
| STMN2 | GLIS1 |
| MMP10 | RBX1 |
| TGFBI | DUSP1 |
| AKR1B10 | RANGAP1 |
| TWF1 | MFF |
| MAPK12 | ANXA11 |
| SEMA4D | POLR2L |
| POMC | SRF |
| HSPD1 | SRSF1 |
| KPNB1 | CBX2 |
| GSC | RBBP4 |
| HMGA1 | KDM2B |
| SUFU | RBBP7 |
| GAB1 | CPSF7 |
| PFKP | MIR105-1 |
| PBX1 | MIR105-2 |
| CAPNS1 | GZMB |
| PTGS1 | GDF9 |
| F2RL1 | RACK1 |
| GZMB | SELENON |
| MTAP | ADORA2B |
| IDH2 | PKN1 |
| YWHAQ | UPF1 |
| TUBB4A | CCT4 |
| PRKD1 | PEA15 |
| SCRIB | MIR124-1HG |
| LINCMD1 | RPL10A |
| SPAG9 | COL4A3 |
| EFEMP2 | MRI1 |
| RSPO2 | CX3CL1 |
| STYK1 | DDX39B |
| CXCR6 | MYL2 |
| CXCL16 | IFIH1 |
| CTCF | PLAU |
| MICAL2 | PTPN2 |
| PLD1 | JMJD1C |
| RPL34-DT | USP13 |
| LINC01433 | SLC4A7 |
| MIR573 | LATS1 |
| RORA | NUP188 |
| BVES | MIR302C |
| CPEB3 | SPCS2 |
| MIR188 | LAP3 |
| FILIP1L | POFUT1 |
| LINC00589 | SUB1 |
| ZNF407-AS1 | ATXN10 |
| ELN | PGF |
| NUP62 | LMO2 |
| S100A14 | RPS3A |
| PDCD10 | GATAD1 |
| MIR193B | PPP2CB |
| FBN1 | NCBP1 |
| MIR499A | DDX1 |
| NOG | KITLG |
| MIR198 | TBCE |
| TPD52 | TIMM44 |
| MMP12 | CDK8 |
| RGS2 | TMPO |
| LINC00342 | HNRNPA3 |
| LINC01089 | MIR365A |
| FUT8 | SMARCB1 |
| RECQL4 | NENF |
| SLC12A6 | ARRB1 |
| MIR520H | CD28 |
| LZTFL1 | AIFM2 |
| PAWR | NAA40 |
| MIR302D | SEC24D |
| TAS1R3 | XPO1 |
| EPO | HLTF |
| GLS | CAMK2G |
| RBFOX2 | TPM1 |
| DIRAS3 | THBS2 |
| FLVCR1-DT | PPME1 |
| ING5 | TCF4 |
| TP53BP1 | PCBP2-OT1 |
| MST1 | UBE2D3 |
| GIT2 | SERPINH1 |
| CSNK2B | CTNNA1 |
| NACC1 | NELFB |
| RF03967-002 | LINC01618 |
| TCF7 | CENPA |
| PPIA | WDR48 |
| HAX1 | DDX46 |
| GPBAR1 | CTNND1 |
| GALC | SMARCA5 |
| RFLNB | CHCHD4 |
| BCL2L11 | COX7B |
| MIR505 | LRPAP1 |
| TUBA1A | GRB10 |
| CLDN6 | MICU1 |
| CTTN | PKN2 |
| IL23A | CLTC |
| TIMELESS | SCAF4 |
| ESM1 | WDR1 |
| HOXA-AS3 | FAHD1 |
| RASSF6 | SUMO2 |
| CLEC3B | SLC16A1-AS1 |
| H4C1 | LATS2 |
| MED19 | MSN |
| SRCIN1 | PRPF31 |
| RBBP8 | LOC110806262 |
| HOXB8 | PNN |
| FBXL7 | RPL7A |
| CRB2 | DES |
| ATP2B4 | OGDHL |
| ABCA1 | COL18A1 |
| MIR765 | RPS6KA3 |
| TOPBP1 | KAT2A |
| LNCNEF | CASP10 |
| RRBP1 | TNFRSF9 |
| LINC00520 | MIR7-2 |
| CSE1L | ERO1A |
| BRD3 | WDTC1 |
| HOXA5 | PIP5K1C |
| AMBRA1 | SFRP5 |
| MDC1 | H2AC4 |
| NR2C2 | LYPLA1 |
| LINC00106 | CGAS |
| PARK7 | ATP5MG |
| MIR1296 | DARS1 |
| LIMK1 | GMPPA |
| ASF1B | STK39 |
| MORC2 | H3-3B |
| VSIG4 | BPNT2 |
| STK39 | ECH1 |
| REC8 | KPNA1 |
| PLG | MLEC |
| LINC00092 | AB196722-001 |
| FGL1 | AF420032-001 |
| STIM2 | MIR498 |
| ATP5F1A | NRP1 |
| CD74 | FCGRT |
| SENP2 | MT1A |
| PIAS1 | LUM |
| FZD9 | PROM1 |
| EIF2AK3 | KDM1B |
| DUSP6 | USP14 |
| TRPM4 | DIABLO |
| ACTB | TOMM22 |
| SLFN11 | TBXT |
| PIM2 | ALKBH8 |
| KDM8 | PLOD1 |
| ADM | TKTL1 |
| RORC | MIR9-3 |
| STK26 | NRL |
| MAP3K1 | BCL11A |
| IL22 | ELAC2 |
| S100B | MAX |
| ARHGEF2 | IRF5 |
| PLD2 | KDM4A |
| LAMB3 | EPB41L4A-AS1 |
| THBS4 | PSMC5 |
| H2AC18 | CCL7 |
| HECTD1 | CUL1 |
| LBR | AQP3 |
| RGS5 | ERG |
| DSC2 | PRPH2 |
| MAP3K2 | BIRC2 |
| MIR1343 | USP25 |
| TXNIP | RPL36A-HNRNPH2 |
| FBXO11 | PACS2 |
| UBR5 | S1PR2 |
| CCR5 | SURF4 |
| ANXA10 | PSMC2 |
| GLIDR | DNAJA1 |
| MIR148B | CXCL9 |
| ONECUT2 | CCT8 |
| MAP3K20 | FST |
| HDAC4 | TAF5L |
| AKNA | LAS1L |
| FBXL5 | ATP5PF |
| TNK2 | CD81 |
| CCDC66 | CD19 |
| PLEC | CHD1 |
| JAK3 | FUT7 |
| PLAC8 | PGAM5 |
| GANAB | NTRK1 |
| CCL19 | TMX1 |
| ZC3H12A | IRF3 |
| PRDX2 | POLR2B |
| GSK3A | KLRK1 |
| SRXN1 | MIR301B |
| WT1-AS | TSG101 |
| PLOD3 | MIR128-1 |
| MIR216B | AKAP13 |
| ACTN1 | ZMYM2 |
| HNRNPH1 | ACP6 |
| NR4A2 | P4HA2 |
| RAD51 | LINC01587 |
| MIR873 | MAFB |
| OR51E2 | SMARCD1 |
| MIR627 | CRYAB |
| GLO1 | REL |
| COL6A3 | FGF9 |
| TFAP2C | SENP2 |
| CCL22 | GNAS-AS1 |
| MYD88 | ACTA2-AS1 |
| FANK1 | PCAT29 |
| FLNB | DRAIC |
| SIX2 | MAP2K7 |
| UBE2N | MAP3K7 |
| MAZ | NPHP3-ACAD11 |
| SMYD3 | HYMAI |
| NSD1 | NODAL |
| RYR2 | RTCB |
| STEAP2 | RAD23B |
| TRAF4 | MTREX |
| CAVIN1 | TRR-TCT2-1 |
| RPN2 | SLC25A6 |
| ZNF281 | KDM5A |
| SNHG8 | UBE2E3 |
| SPDEF | MIR590 |
| MIR605 | HMMR |
| TMOD1 | CHEK2 |
| MIR944 | ARCN1 |
| MALT1 | EFNB2 |
| TNFRSF6B | CEBPD |
| ADAM8 | PPIA |
| TLK1 | SRPK1 |
| ATAD2 | KTN1 |
| GNA12 | MINPP1 |
| ELK3 | NAP1L1 |
| MCUR1 | BCL6 |
| PLXND1 | MRE11 |
| MGAT3 | TRRAP |
| SCUBE2 | COPB2 |
| SFMBT1 | ITGA5 |
| PCNP | TEX10 |
| MIR639 | BAG6 |
| CHRM2 | EIF2B2 |
| TRAF2 | HAND2 |
| MIR758 | TCP1 |
| CIB1 | PAF1 |
| ATRX | KYAT3 |
| KNSTRN | ATG5 |
| TP53TG1 | MEF2A |
| SYNE2 | EIF3A |
| MIR7-2 | BZW2 |
| AFF2 | UBE2N |
| CTSD | MCM7 |
| GFAP | H2AC20 |
| PHB1 | RAB11A |
| USP13 | USP48 |
| RAD18 | HILPDA |
| COPS3 | LYPLAL1 |
| NEBL | POLDIP2 |
| SULF2 | S1PR1 |
| SEMA3E | INPP4B |
| CHST11 | HGS |
| NR3C2 | FIRRE |
| TYRO3 | IPO7 |
| CACNA1G | RRP8 |
| ACTG2 | ZBTB7A |
| DNAJC6 | RPL9 |
| MIR671 | MIR524 |
| USP29 | HMCN1 |
| CALB2 | BRAT1 |
| MMP11 | HSPA1B |
| COPS8 | RPS8 |
| BRF2 | FSTL1 |
| CCNDBP1 | METTL5 |
| MOAP1 | NRG1 |
| MIR598 | PTGDR2 |
| APOA1 | P3H1 |
| G3BP2 | BPHL |
| EIF3I | E2F3 |
| LINC01503 | ENSG00000285988 |
| CTDSP2 | PLAA |
| PURPL | TNFAIP8 |
| CAHM | COPS4 |
| EHHADH-AS1 | TFE3 |
| RBM5-AS1 | EDARADD |
| CDH13 | CTR9 |
| TKT | NFATC4 |
| CCBE1 | HDAC6 |
| GAP43 | BID |
| MAGI2 | ENSG00000277966 |
| ZIC1 | ENSG00000278592 |
| AKT1S1 | ENSG00000273961 |
| DYNC1H1 | ENSG00000276496 |
| LINC00707 | ENSG00000278020 |
| ENSG00000285743 | ENSG00000278334 |
| ENSG00000285845 | BAZ2B |
| LINC00473 | CIB1 |
| TPM2 | BAG5 |
| EPS8 | SOX10 |
| NFIB | MIR147B |
| PITX2 | SOX4 |
| NKX2-5 | IGFBP6 |
| SLCO1B3 | EIF5B |
| HCP5 | CASP7 |
| MANCR | TIAM1 |
| MIR653 | PDE4B |
| LOC124906209 | GDNF |
| GPC1 | RBFOX1 |
| MIR346 | PSMD7 |
| OSMR | LGR5 |
| IFITM1 | MYF5 |
| PHLDA2 | NPEPPS |
| SOX9-AS1 | UNC45A |
| BCL6 | AGBL4 |
| NR3C1 | DDB2 |
| BIN1 | ADAMTS10 |
| NPPA | RPL19 |
| MIR942 | PREB |
| APPAT | CLPP |
| ENSG00000277701 | COPS3 |
| LOC100506076 | DAPK1 |
| S1PR3 | AGTPBP1 |
| IMMT | PRMT3 |
| PSTPIP1 | ERAL1 |
| ERBIN | H3C12 |
| ZNRF3 | TAF6L |
| KIF23 | H3C3 |
| CEACAM3 | TUBB4A |
| PAK2 | TBX5 |
| WNT6 | CEP55 |
| THY1 | PEBP1 |
| PRLR | IPO9 |
| LINC01082 | SCN10A |
| MIR662 | NUCB1 |
| SLC12A2 | CCNB1 |
| HCK | NUDT9 |
| ABI1 | GABPA |
| DUSP4 | EIF5A2 |
| GFPT2 | NECTIN2 |
| CCL17 | PRKCE |
| DCBLD2 | MIR155HG |
| IL13RA2 | ATP2B1 |
| REPIN1 | ERH |
| TRPV6 | NFYB |
| FOLR1 | U2SURP |
| GDF2 | MAPKAPK2 |
| IGFL2-AS1 | UBE2L3 |
| MAGEA3 | SPATA20 |
| HSPB8 | RTF1 |
| ZBTB16 | MIR3662 |
| RAC3 | FRK |
| NFATC3 | MTX1 |
| LOXL1 | IFI16 |
| SOX6 | CAMK4 |
| STK33 | OVOL2 |
| ST8SIA1 | MYB |
| CRIP1 | SUZ12 |
| GPR32 | EIF2B3 |
| ELSPBP1 | UBB |
| MIR384 | BMI1 |
| SNX27 | ATP6V1G1 |
| TLR9 | MYOF |
| EIF5A | CHCHD2 |
| KARS1 | ABHD11 |
| NRTN | WNT4 |
| CXCR1 | KRT1 |
| CD14 | SPCS1 |
| MAPK11 | BPTF |
| DUSP5 | H1-0 |
| RALB | SLC5A8 |
| SLC22A1 | MIR381 |
| RBFOX3 | TMPRSS2 |
| TP53I11 | MDM4 |
| MIR374B | TRIM25 |
| FOXC2-AS1 | CTTN |
| MIR507 | IRF7 |
| MAP4K4 | EEF1B2 |
| S100A6 | GABARAPL1 |
| TJP2 | MT1G |
| IRX2-DT | SMYD2 |
| UGDH | NOC4L |
| DLL4 | HINT1 |
| ARHGAP35 | DDX24 |
| IATPR | TJP1 |
| KIF5B | DPPA3 |
| MIR889 | MIR31HG |
| MCTS1 | PHB2 |
| MRPS7 | CASD1 |
| SULT2B1 | UTRN |
| PDLIM1 | HDAC7 |
| MPHOSPH8 | TP53BP1 |
| PTBP3 | MIR4443 |
| H2AZ1 | RO60 |
| PLAT | PITPNC1 |
| MIR744 | BCORL1 |
| MIR3609 | FCGR3A |
| LINC01305 | SP3 |
| MIR642A | CHD4 |
| NCOR2 | TTC4 |
| RPL4 | LINCMD1 |
| PSORS1C3 | EBF1 |
| RRM2B | TUBA4A |
| SERPINB2 | H3C4 |
| REL | FZD6 |
| ABHD5 | PDLIM5 |
| RELN | CDK19 |
| FHOD1 | SNTA1 |
| GSDMD | MIR101-1 |
| NCOA2 | RAB1A |
| STK4 | KDM3A |
| LMNB1 | CBX1 |
| PELP1 | MYO18A |
| MIR19B2 | CCR7 |
| MYOCD | FXR1 |
| RAE1 | SUPT5H |
| FLG2 | MIR561 |
| TTTY15 | ILVBL |
| MDM4 | INO80 |
| FAM83A-AS1 | ASF1A |
| CUL5 | TLR9 |
| CNTN1 | ASXL1 |
| TRAF5 | MBNL1 |
| WDR48 | MIR519C |
| USP19 | SSR1 |
| NOC4L | LINP1 |
| MIR487A | MCAM |
| YWHAH | EPCAM |
| H4C14 | PBRM1 |
| CCT5 | REXO2 |
| ADGRG1 | DAB2IP |
| HSPG2 | IL9 |
| TUBB4B | NR6A1 |
| OS9 | MRPS9 |
| MIR4521 | NEDD4 |
| JDP2 | USP15 |
| CARMIL2 | SETD1A |
| FUS | ISG15 |
| KDM2B | ZNHIT1 |
| VAV2 | GTF2E2 |
| CYBRD1 | MTPN |
| HERC3 | TUBA1B |
| DUSP26 | PGRMC2 |
| MIR544A | KDM8 |
| SCARNA14 | PRKCI |
| MCU | THOC1 |
| MGAT1 | NDUFB6 |
| MAGED1 | MBD2 |
| LAMTOR5 | IL23A |
| USP5 | TBX20 |
| RPL12 | VAMP2 |
| SYNE1 | H3C6 |
| KIF18B | BACH1 |
| MAP3K9 | PRRX1 |
| CLTC | GIMAP8 |
| ACAT1 | OTUB1 |
| USP18 | MIR675 |
| PHB2 | RRAGC |
| CHFR | WAS |
| UBQLN1 | TRIP12 |
| MAVS | MIR512-1 |
| RBBP6 | NDRG2 |
| ENSG00000285517 | H1-4 |
| GLIPR1 | RPS4X |
| SERPINI1 | CDC25A |
| CELF1 | IGFBP7 |
| MIR133A2 | DCTN4 |
| HCG18 | PPM1F |
| H4C13 | PTF1A |
| H4C15 | SCAMP2 |
| RNF2 | ATP5ME |
| NVL | GTPBP4 |
| PPP1R10 | MIR642A |
| CS | SNRNP70 |
| PPP3CB | H2AX |
| HEG1 | C5 |
| PCAT19 | PXN |
| MIR1469 | FBL |
| STRAP | ACVRL1 |
| KLK2 | RUVBL2 |
| H4C9 | PIWIL4 |
| LIN28B | CAPRIN1 |
| GSTM3 | RAB5C |
| SRGN | SMAD7 |
| ACOT12 | ITGA6 |
| EFHD2 | PIWIL2 |
| EPB41L4A-AS1 | NTF3 |
| MIR147A | RBM4 |
| LINC00515 | COL11A1 |
| PPP6C | H3C7 |
| PKHD1 | KRT3 |
| MMP8 | TMED10 |
| PCSK1 | TRIM35 |
| H4C3 | SNORA73B |
| H4C11 | ITGAV |
| H4C2 | ITGB4 |
| H4C5 | LINC00472 |
| H4C8 | CBX7 |
| H4C12 | SLC2A14 |
| H4C4 | H3C2 |
| H4C6 | ITK |
| UBE2D1 | ZBTB16 |
| PTGER4 | NOTCH4 |
| ITGB2 | STAT2 |
| SRGAP1 | MIR340 |
| TNS1 | TBC1D5 |
| FGF9 | FGF4 |
| BGLAP | PCYOX1 |
| TMPRSS2 | UHRF1 |
| DIDO1 | H3C11 |
| SBF2-AS1 | H3C10 |
| UNC5B-AS1 | H3C8 |
| LINC02893 | MRTFA |
| ENSG00000259222 | FKBP4 |
| FOXK2 | FOXG1 |
| NCAPG | NAE1 |
| PRKACA | NDUFS5 |
| SNORD88C | CRMA |
| LINC01354 | RECQL |
| GREP1 | MYH10 |
| LINC01637 | LOC109504728 |
| PDGFB | IPW |
| GADD45A | BICDL3P |
| CA3 | COL6A1 |
| CHERP | CCS |
| MIR17HG | PARP2 |
| WNT10A | CIITA |
| CHRM1 | HCK |
| CST1 | LYVE1 |
| DIP2C | VEGFC |
| NUPR1 | EIF2S2 |
| ADAM12 | WDR77 |
| JUND | HLX |
| MIR599 | MIR526B |
| FOXCUT | MANF |
| CDH4 | HNRNPR |
| CD81 | SLC25A24 |
| KLK14 | FLOT2 |
| CASR | RPL17 |
| RAB11A | CALCR |
| MIR326 | EIF5A |
| HAS2-AS1 | LEMD3 |
| MARVELD2 | MIR522 |
| HOXC6 | MSX1 |
| MAL2 | PPIF |
| LINC00671 | SMO |
| DNAJA1 | ERP29 |
| CRMA | KRR1 |
| TRAF3IP2 | DPY30 |
| SMG1 | MARK4 |
| MIR196B | RNF20 |
| BRAP | GPC6 |
| MIR1204 | SNORD24 |
| MIR2116 | BRIX1 |
| FAM88B | GTPBP10 |
| CCL11 | UNG |
| FBLN5 | MLLT10 |
| NFE2L3 | PSMA5 |
| PSAT1 | PDE6A |
| VSIG1 | RBM20 |
| LINC00115 | STK4 |
| PANTR1 | CRX |
| MIR588 | USP26 |
| LOC101928626 | PTPRD |
| ENSG00000238009 | TAF4 |
| ENSG00000239945 | LIMA1 |
| ENSG00000290385 | CCR2 |
| SMC3 | ITGA2B |
| DHX9 | PSMD6 |
| TNFAIP6 | EED |
| TF | MIR302B |
| CHD3 | ZBTB7B |
| PRPF4 | CSTF1 |
| SNORD95 | ARHGEF2 |
| MYO7A | DNAJC10 |
| MMP16 | MYOCD |
| BCLAF1 | AGO1 |
| CTBP1-DT | CHAF1A |
| MIR652 | TOX4 |
| SRP68 | HCCS |
| MIR802 | EIF3L |
| MIR1284 | P2RY2 |
| LINC00462 | ARMT1 |
| LINC00702 | CYFIP2 |
| MIR527 | RAB2A |
| MIR934 | ABHD10 |
| RBM47 | CD86 |
| ACTA2-AS1 | SAFB |
| ASH2L | BRD3 |
| NCAPD3 | SPCS3 |
| EFNB1 | LY6D |
| POMGNT1 | AP2B1 |
| SDC4 | TTLL1 |
| TRIM37 | RNA18SN1 |
| DNAJB4 | CISH |
| LUC7L2 | PTPRC |
| SFTPC | RAB21 |
| F2RL2 | ACTL6A |
| MANF | PAX8-AS1 |
| MT1M | AREG |
| PTH | NKX6-1 |
| PECAM1 | SNHG7 |
| SRSF6 | WNT1 |
| SOX17 | RBL2 |
| PTPRC | MIR507 |
| LRRC8A | TUBA8 |
| APLF | ZNF322 |
| NR4A1 | DST |
| CA12 | ITCH |
| IGFBP6 | RBFOX2 |
| P4HA1 | PRIM2 |
| CNPY2 | GDI2 |
| IL27 | LORICRIN |
| BNC1 | MYO1C |
| ASAP1-IT1 | MIR372 |
| CHD8 | RBM10 |
| RAB8A | PEG10 |
| CRP | SMC4 |
| ROCR | CASP6 |
| ENSG00000288605 | NPLOC4 |
| LOC102723517 | ZFP91 |
| TNNT1 | NPM2 |
| XPO5 | PWAR6 |
| HYAL1 | TMEM43 |
| SRPK1 | UBR4 |
| VASN | CSE1L |
| ULK1 | TIMM13 |
| ARPC2 | DDX4 |
| HHIP | JUNB |
| SCARA5 | NEK9 |
| TRAPPC9 | CPSF1 |
| LAMP3 | FAM98A |
| OAS2 | RPL21 |
| STAMBPL1 | PTP4A2 |
| NDUFAF5 | RAB15 |
| MESP1 | SUPT6H |
| IFIT5 | TEAD1 |
| ARRDC1 | RBBP8 |
| MIR1224 | RUNX3 |
| MIR1228 | SIX1 |
| SELL | GABARAPL2 |
| ACAN | RBM39 |
| UBE2E3 | LINC03033 |
| LRRFIP1 | MIR551A |
| CSNK1E | NCLN |
| MIR500A | CHEK1 |
| HMGB3 | BCL7B |
| NR1H4 | FADD |
| CRTAC1 | PPP1R1B |
| TCF15 | DNAJC5 |
| WIF1 | AP3B1 |
| MYL2 | CAMK1 |
| CHN1 | ARF6 |
| CYP7B1 | SGCE |
| IKZF3 | RNF40 |
| NEUROG3 | NDUFB4 |
| SNX1 | NDUFB5 |
| CLIC4 | RP2 |
| DPY30 | KCMF1 |
| DERL1 | UBXN7 |
| FOXL2 | CDK7 |
| CKAP5 | RAB32 |
| USP48 | PTGER4 |
| MAML3 | GLI1 |
| OPN3 | PTTG1 |
| SNRPC | FGF3 |
| FAM83A | RRBP1 |
| MIR135A2 | STOM |
| MIR105-2 | KLF9 |
| USP17L9P | KLF16 |
| PTX3 | BHLHE40 |
| CPEB2 | TUBA1C |
| SH3PXD2B | FHL1 |
| PHACTR1 | FGF1 |
| RND1 | COPG2 |
| SUPT20H | HDGFL2 |
| RAN | NBR1 |
| CBR1 | CBX4 |
| CHD2 | MT1X |
| IL13RA1 | CD276 |
| MBD1 | FKBP10 |
| OGA | GAS6 |
| WDHD1 | SRP14 |
| ERH | ST7-OT3 |
| CDK2AP1 | TAF1 |
| HNRNPLL | CLK3 |
| TMEM88 | MARK2 |
| CBX7 | PNO1 |
| SCN5A | PPP1R8 |
| MIR181C | POMK |
| SPOP | MAPK13 |
| ARNT | RSL1D1 |
| TBX20 | ATP1B3 |
| ALDOB | DHX36 |
| CCR3 | PLOD3 |
| MARK2 | TRG-GCC3-1 |
| ASXL1 | FOXL2 |
| DUOX2 | ATP6V0C |
| ANXA7 | GIPC1 |
| IL17F | SEC22B |
| PUF60 | PLUT |
| DGKZ | POU2F1 |
| PEA15 | GDPD1 |
| RCE1 | PSMA3 |
| TNFSF12 | TAB1 |
| NUB1 | FUBP3 |
| BHLHA15 | LGALS9 |
| FBXL14 | MLLT1 |
| TSLP | EXO1 |
| RASSF10 | SCCPDH |
| MIR766 | CHRM2 |
| LINC00886 | RBBP5 |
| MIR3175 | MIR663A |
| MIR515-2 | MIR10A |
| LINC01134 | PCAT19 |
| LINC01819 | KLK1 |
| MIR3141 | FKBP5 |
| ENSG00000236283 | TUBA1B-AS1 |
| SMAD5 | STAG2 |
| XPR1 | OTX2 |
| FNBP1 | MIR135B |
| ARHGEF10L | IRAK3 |
| KEAP1 | COA7 |
| MIRLET7F1 | MKRN3 |
| PHF13 | SP110 |
| MSLN | BNIP3L |
| AGK | ZAP70 |
| FABP12 | TRAF2 |
| EIF2S1 | ERG28 |
| TSPAN1 | RTRAF |
| SPHK2 | AHNAK |
| PLCD1 | DVL3 |
| TPM3 | HINT2 |
| ITGB5 | LMCD1 |
| DVL3 | TBC1D8 |
| CPB2 | SF3B3 |
| ATXN1 | RCN2 |
| GATAD2B | XPO5 |
| KPNA3 | LNMICC |
| TXN2 | MPHOSPH8 |
| SLC25A10 | ACTR3 |
| EXOC7 | MRPS23 |
| OLA1 | LYAR |
| TRIM5 | RNY1 |
| DECR1 | LINC00461 |
| CHAF1B | MIR371A |
| AFAP1 | PSMA4 |
| AFF1 | WEE1 |
| LUC7L3 | EIF3B |
| NCAPG2 | UBE2E1 |
| SYPL1 | SRSF3 |
| TRA2A | FER |
| AKAP10 | DOCK2 |
| MSRB1 | RAB5IF |
| RAD51AP1 | SERPINB5 |
| DNASE1L2 | MAG |
| JADE2 | IPO5 |
| SNORD47 | BCL2A1 |
| PGRMC1 | SF3A3 |
| TFF3 | KDM2A |
| CSF1R | TYK2 |
| ODC1 | HRNR |
| MACROH2A1 | IL18R1 |
| MIR4443 | STARD3NL |
| SOX1 | CSTF3 |
| TUBG1 | UTP20 |
| MIR665 | SEPTIN2 |
| BFSP1 | MBD5 |
| DLGAP1-AS2 | ADPRS |
| CAMK1D | ACBD3 |
| SPTLC1 | TUBG1 |
| CBLB | RALY |
| PTPRB | GDI1 |
| SEMA4A | OSBP2 |
| USP1 | RELB |
| EYA4 | YTHDF3 |
| RB1CC1 | HDGF |
| SLC22A2 | MCM3 |
| ARHGEF11 | MSRB2 |
| CDCA7 | UBR5 |
| PTAFR | RAB11B |
| CAP2 | ZFP57 |
| CRMP1 | CHRNA7 |
| KIF3A | H3-4 |
| RHOG | TRIB2 |
| TTC5 | NEUROG2 |
| EPS8L2 | ADGRB1 |
| PEG10 | DNM3OS |
| TSPAN5 | SAP18 |
| C1D | NES |
| CHMP3 | GABARAP |
| MED27 | KLF2 |
| MEMO1 | RAE1 |
| SCYL2 | FEV |
| TRIM9 | NOP16 |
| ASCC2 | CRNKL1 |
| KCNIP3 | TMED2 |
| NKX3-2 | PXDN |
| SCIN | FHOD3 |
| ULBP1 | SNX27 |
| MARCKSL1 | MRPS27 |
| RIOX2 | TOR1AIP2 |
| SPSB1 | TMC8 |
| TRIM55 | H2BC12L |
| DNTTIP1 | MORF4L1 |
| EVA1A | UBA2 |
| LECT2 | CCL15-CCL14 |
| PPP2R2D | TMED9 |
| DND1 | TP53TG1 |
| MUC15 | ACTA2 |
| MARVELD1 | GDF3 |
| SAT1 | MIR4492 |
| MAP3K3 | APAF1 |
| NRCAM | CCNA1 |
| PPP1R13B | SETD1B |
| NCL | P4HA1 |
| BBC3 | DPP9 |
| ENAH | SMARCE1 |
| TNFRSF11B | RNY4 |
| ETV1 | CLIC4 |
| CADM1 | CDC20 |
| MIR448 | CASZ1 |
| FLRT3 | MIR543 |
| EPB41L1 | PHPT1 |
| LAIR1 | IRAK4 |
| ARAP1 | VNN2 |
| ATG4A | PARS2 |
| CYB5R1 | OCIAD1 |
| PRRT2 | YTHDC1 |
| VPS4A | MERTK |
| ADAMTS12 | SRSF6 |
| KIF3B | MIR1246 |
| PCGF2 | BRD8 |
| TRIM27 | MRPS35 |
| UTRN | TRMT2A |
| WSB1 | KRT13 |
| PITPNM1 | PHOX2B |
| PRUNE1 | KRT2 |
| RIOK1 | CLIC1 |
| AKTIP | ITGA3 |
| EXOC8 | UBE2V2 |
| HES6 | PHF10 |
| IL36G | DAD1 |
| ING3 | HUWE1 |
| USP34 | MTA1 |
| CNKSR1 | SCGB2A1 |
| CSMD1 | DCAF1 |
| EPN3 | VEGFB |
| EXOC1 | GAS6-AS1 |
| LVRN | CEBPA-DT |
| NOB1 | MRPS30 |
| PPP6R3 | LGALS3BP |
| YPEL3 | NCBP2 |
| CNOT6L | GATA5 |
| CUEDC1 | RCAN1 |
| PDCD2 | POSTN |
| SERPINB4 | SMARCA1 |
| SIVA1 | SIAH1 |
| CIAO2A | MKNK2 |
| LGALSL | GIT1 |
| PTH2 | NASP |
| RNASE7 | MCPH1 |
| TRIM46 | GNA13 |
| ZNF574 | UTP14A |
| CLEC5A | PROCR |
| FBXL18 | CXCL5 |
| RBM12B | TET2-AS1 |
| RPP25 | TRIM21 |
| SECISBP2L | HIF1AN |
| TP53INP2 | S100G |
| TRIM50 | UBA6 |
| ZNF460 | TAOK3 |
| ABRACL | CLEC7A |
| JADE3 | NOD1 |
| LHFPL3 | MYDGF |
| TMEM92 | CXCR6 |
| C3orf70 | MILIP |
| APCDD1L-DT | LILRB1 |
| MIR548C | NKX6-2 |
| MIR1181 | RCN1 |
| MIR1285-1 | NT5DC3 |
| TAGLN2 | TFCP2L1 |
| PPP3CC | ENSG00000261069 |
| HNRNPU | ISL1 |
| PALLD | STRAP |
| PMEPA1 | SLIRP |
| RETN | MRPL37 |
| KPNA2 | SARNP |
| QKI | S100A4 |
| LAMA4 | CHCHD3 |
| RSPO1 | MIR100 |
| MIR520C | UBE2O |
| KAT2A | ZC3HAV1 |
| ALOX12 | FLOT1 |
| AHNAK2 | IL32 |
| CCNG2 | HHIP |
| ZNF778-DT | NUPR1 |
| GRIA3 | PUF60 |
| KAT6A | VTA1 |
| GRIK3 | NDUFA3 |
| EPHA8 | RSPO3 |
| TNKS2 | TMEM106B |
| FCN2 | ULK2 |
| RAPGEF1 | PTMA |
| GP5 | NEK2 |
| HK3 | KIF11 |
| ONECUT1 | EIF3I |
| PCDH9 | H2BC8 |
| C1GALT1 | KRT12 |
| NLRX1 | LAMP3 |
| PADI1 | SEC16A |
| URI1 | MYCNOS |
| KRT6C | BRD7 |
| SLC2A12 | H2AC1 |
| ARHGAP21 | SFRP2 |
| ATP6V0D2 | ARL6IP5 |
| KDM5D | CEBPE |
| SENP7 | HAS2-AS1 |
| ART1 | CASQ2 |
| KIAA1217 | RDX |
| ADGRF1 | ZFP36 |
| DMKN | EGLN3 |
| SYDE1 | RALA |
| TMT1B | MIR129-1 |
| DCAF15 | LAMC1 |
| JMJD8 | SPINT1 |
| ZNF382 | CFL2 |
| IRF1-AS1 | HSPA14 |
| TYMSOS | RBP3 |
| TTC28-AS1 | CPEB1 |
| SNAI3-AS1 | PRICKLE2 |
| LINC01060 | PFDN4 |
| CACNA1C-AS2 | VBP1 |
| MIR1285-2 | CELSR1 |
| LOC90246 | ALCAM |
| LOC111589215 | ELAVL3 |
| OA985516 | PPRC1 |
| CSNK2A2 | MIR517A |
| ACP3 | NONHSAG046336.2 |
| ISG15 | MAPK12 |
| SRI | TMPO-AS1 |
| MIR939 | CRYZ |
| CAND1 | IRX2-DT |
| LPL | MIR16-2 |
| DLL3 | MARCKSL1 |
| EIF6 | CXCL16 |
| CHD5 | ANKRD22 |
| CD9 | RBL1 |
| ST14 | TRV-CAC1-5 |
| RNF217-AS1 | TRV-CAC1-6 |
| MEOX2 | TRV-CAC1-1 |
| KRBOX4 | TRV-CAC1-2 |
| GPX1 | TRV-CAC1-4 |
| TUBA4A | TRV-CAC1-3 |
| PRSS1 | TRV-CAC1-7 |
| RPLP2 | TRV-CAC5-1 |
| AP1G1 | KMT2B |
| BTAF1 | MIR409 |
| LTB4R2 | PWAR5 |
| HOXD13 | KRT9 |
| FBXO32 | HDAC5 |
| PCDHA3 | EREG |
| CROCC | CXCL3 |
| FCGBP | OSMR |
| EMX2OS | PIAS3 |
| USP41 | CYFIP1 |
| KLF3-AS1 | AF465954-001 |
| LINC00839 | NDUFB1 |
| SNORA71A | VCPIP1 |
| CCND2-AS1 | RPL24 |
| MIR1266 | SCGB2A2 |
| MIR1976 | DINOL |
| MIR4429 | H2BC3 |
| SNORA71B | SNAP23 |
| ARAP1-AS2 | CENPM |
| LINC01816 | MSRB3 |
| MIR762 | SNORD43 |
| SUCLG2-DT | SNRPD1 |
| ITGB1-DT | CAPZA1 |
| LINC00842 | USP28 |
| LINC00900 | MIR133A2 |
| LINC00924 | CXCR2 |
| LINC02126 | CACNA2D1 |
| LINC02470 | CAMK2D |
| MIR3194 | RFC5 |
| MIR4513 | SLIT2 |
| MIR711 | MYO6 |
| MKLN1-AS | OXCT1-AS1 |
| SNORD138 | SRI |
| LINC01451 | CAPNS1 |
| MIR4476 | MIR551B |
| MRPS30-DT | HAVCR2 |
| MIR1252 | INA |
| MIR4744 | CCL8 |
| TMEM123-DT | GDF11 |
| MIR1243 | HSPA1L |
| MIR2113 | OGFR |
| FLJ32255 | PRPF40A |
| MIR3935 | SMDT1 |
| ENSG00000250697 | EMC4 |
| ENSG00000265477 | PLXNA4 |
| ENSG00000271788 | SLC48A1 |
| MIR6780B | NDUFA4L2 |
| C5-OT1 | ELF3-AS1 |
| CHCHD4P4 | RAD23A |
| ENSG00000273361 | MKNK1 |
| MIR8084 | FBXL3 |
| AKR1B10P1 | MIR495 |
| ENSG00000268108 | PTPN13 |
| ENSG00000289206 | EIF3C |
| AKAP13 | PIGR |
| ANKRD11 | HAGLROS |
| PABPC1 | ARID1B |
| FZD1 | NOLC1 |
| DARS1-AS1 | GLG1 |
| NTSR1 | CCR4 |
| DDB1 | ACTG2 |
| CHRNA7 | KRT5 |
| CSF1 | FAM72A |
| SIN3A | VSIR |
| ETV5 | CTCFL |
| FBXO45 | PES1 |
| HELLS | IPO4 |
| KLK7 | UBR1 |
| LBX1 | YKT6 |
| CAPRIN1 | MIR135A1 |
| MIEN1 | CAPZB |
| CLIP1 | MRPS5 |
| SOCS2-AS1 | MIR1248 |
| LAMA1 | HSPH1 |
| CYP3A5 | NAP1L4 |
| FBLN1 | CDC37 |
| FKBP5 | MIR539 |
| UCHL1 | ARL8B |
| PDHA1 | KDM5B |
| CIRBP | DMPK |
| MIR324 | RELN |
| MIR522 | CNOT7 |
| RND3 | PLEKHG5 |
| KRT6B | CYTL1 |
| OTX1 | MAP3K14 |
| NKX2-8 | PHF6 |
| PRR11 | ALOX12-AS1 |
| RPSA | LINC00243 |
| COL7A1 | HOXA9 |
| DPYD | DYNC1LI1 |
| NEDD8 | KDM4B |
| HDAC7 | RN7SL1 |
| PABPN1 | SLC2A1-DT |
| SPTA1 | LPAR1 |
| MGST1 | FLG2 |
| KRT3 | SIAH2 |
| RAB5A | MIR578 |
| TEX10 | AGR2 |
| CARD10 | BAG3 |
| STOX1 | KAT8 |
| PIK3C3 | WASF2 |
| VASP | CLEC4A |
| BPTF | HIF1A-AS1 |
| SYNPO2 | OLMALINC |
| FCGR2A | APOL2 |
| MELK | LAT |
| RMI1 | LTK |
| RRM1 | HIPK2 |
| ORAI1 | EOMES |
| POLR2A | UNC5B-AS1 |
| RCCD1 | MIR4485 |
| MOSPD1 | ROBO1 |
| FTH1 | SLBP |
| ITGB7 | AQP1 |
| ATP2C2 | SNORD55 |
| INHBB | SORBS2-AS1 |
| MB | TAF9 |
| H1-3 | MIR7-3 |
| ID3 | UBAP2L |
| ENTPD3-AS1 | SS18 |
| MIR502 | TBX3 |
| PCBP2-OT1 | H2AC19 |
| TJP3 | MAD1L1 |
| SELENBP1 | PDPN |
| SMOC2 | H2BC1 |
| CNTROB | HSPE1 |
| SNX6 | H2BC11 |
| LUZP1 | TLR7 |
| MIR154 | UCHL5 |
| SNHG15 | EIF3D |
| PRSS3 | NIPSNAP2 |
| ALPP | FKBP9 |
| USP10 | H1-2 |
| FIRRE | MDS2 |
| CTSK | NCAPG |
| BMAL1 | ADGRE5 |
| CBS | MIR4435-2HG |
| MIR378E | MYOG |
| ITK | HEATR1 |
| GPC6 | COPG1 |
| IRAK2 | DNAJC7 |
| TERF2IP | SETD5 |
| NOC2L | HSPA2 |
| SHROOM3 | HSDL2 |
| MIR99AHG | NIBAN2 |
| APOE | SNAI2 |
| YES1 | GUCA2B |
| LTF | TERF1 |
| RARS1 | AQP5 |
| HP | MBD6 |
| TRPV4 | LOC126860933 |
| WWP2 | SEPTIN9 |
| OPCML | TXNL1 |
| NR1H2 | OIP5-AS1 |
| CTSA | NUMA1 |
| KHSRP | ABCF2 |
| GTSE1 | SERPINB6 |
| SRSF1 | NCKAP1 |
| CAVIN2 | HOXC10 |
| RNF114 | FKBP3 |
| PEBP4 | SNORD95 |
| BOK | RAD18 |
| POMT2 | CXCL13 |
| PNPLA3 | EPIC1 |
| RIF1 | AXL |
| CIZ1 | HES1 |
| MEX3B | RYBP |
| ADAMTS15 | LOC108942766 |
| SYNE3 | CXCR5 |
| DPEP3 | COX7A2 |
| ZFPL1 | UBE2A |
| LYRM1 | SEC11C |
| CCDC7 | P2RY11 |
| CBLL1 | SUPT16H |
| DBN1 | SUV39H2 |
| KRT10 | LINC00470 |
| GPNMB | PHLDA3 |
| MIR524 | BCOR |
| TRIM44 | MYBL2 |
| MIR637 | TIMM23 |
| ATP1A1 | LRP4 |
| RARA | MIR101-2 |
| OVOL1 | CDK11B |
| LY75 | H2AC21 |
| WFDC2 | CSRP3 |
| NUDT1 | BZW1 |
| LINC00449 | H1-10 |
| LINC01232 | HCG18 |
| COX5A | KCNA5 |
| C10orf143 | TNFAIP6 |
| GABPB1-AS1 | MIR708 |
| CLMAT3 | FOXC1 |
| BTG3-AS1 | LINC00511 |
| PPP1R12B | CRTAP |
| PPP1CA | REXO1 |
| ALOX5 | KCNE1 |
| DEPTOR | SKIC8 |
| RBM38 | INSL5 |
| MIR3936HG | LMX1B |
| HLA-A | FOXF1 |
| PRKD2 | DSG2 |
| P2RY11 | PAM16 |
| STK36 | CAP1 |
| NDUFA2 | LIMS1 |
| SLC7A14 | PTP4A3 |
| EEFSEC | MRPL40 |
| EML4 | ZC3H12A |
| MYH8 | ISOC2 |
| PARP6 | LYST |
| CLGN | DLG1 |
| CLPTM1 | MIR612 |
| ACAP2 | DPPA5 |
| RAPGEF6 | LNCPRESS1 |
| ZNF318 | EDIL3 |
| YIPF1 | IFT88 |
| ALDH16A1 | EYA1 |
| BBX | TRX-CAT1-2 |
| CCDC93 | TRX-CAT1-8 |
| IGSF10 | TRX-CAT1-1 |
| SPATA18 | TRX-CAT1-3 |
| TCL1B | TRX-CAT1-4 |
| TSGA10 | TRX-CAT1-5 |
| CENPL | TRX-CAT1-7 |
| MEX3D | TRX-CAT1-6 |
| UBL7 | CUL9 |
| EMX1 | SUN2 |
| FAM13B | LEO1 |
| SERHL2 | IL19 |
| TBC1D31 | NEB |
| ZC3H15 | NKX3-1 |
| ZER1 | ATL3 |
| IQCN | POP1 |
| SOWAHB | MAP1B |
| PDZD9 | MIR302CHG |
| FSBP | CNN2 |
| IQCF2 | SZT2 |
| MIR193BHG | HOXB13 |
| TCP1 | MIR501 |
| LPAR1 | TLR5 |
| TRIM2 | GPKOW |
| ARG1 | MAP4K4 |
| FST | JMJD6 |
| SLAMF6 | DSC3 |
| MIR28 | CXCL11 |
| CD109 | MS4A15 |
| PYCR1 | THY1 |
| XRN2 | AKAP8 |
| MTSS1 | PURA |
| DCD | RRAD |
| ANXA3 | HCFC2 |
| TNFAIP3 | EPHB2 |
| PAX3 | AFF1 |
| CP | ZFPM1 |
| TUBB2A | SEPTIN7 |
| MIR4492 | USP22 |
| ADAMTS9-AS2 | METTL1 |
| CAP1 | TPM2 |
| SCN10A | OA985516 |
| EXOC5 | CKAP5 |
| HPSE2 | CD24 |
| EFL1 | SCAI |
| ME3 | PIM2 |
| IRX4 | FAM136A |
| QSOX2 | PDLIM1 |
| SNED1 | EHD4 |
| TRO | WIF1 |
| APOL3 | LONRF3 |
| HAPLN4 | RREB1 |
| ARMC2 | TAL1 |
| CLNK | ORAI1 |
| SEPTIN12 | TMT1A |
| TMC4 | HIRA |
| CAPRIN2 | TTLL12 |
| CC2D1B | EIF3F |
| FAAP100 | FAM225A |
| MEX3A | RING1 |
| PCDHGA10 | ID4 |
| METTL16 | SPOP |
| PKD1L2 | UHMK1 |
| VIRMA | TBCA |
| KRT39 | ACTG1P25 |
| EID2 | GAP43 |
| CMTM1 | EEF2K |
| LINC01550 | PRAME |
| CITED2 | CTNND2 |
| PSAP | LRRC59 |
| LY6D | MAP3K3 |
| PLCB1 | LAMA4 |
| ADAMTS6 | H2AZ2 |
| TRPM2-AS | TWF1 |
| BACE2 | ID3 |
| NCSTN | PARVA |
| CLIC1 | MEST |
| TRIM14 | UBE2B |
| RASA1 | GOLGA5 |
| HOXA1 | IL37 |
| FOLH1 | TOMM70 |
| TRPV1 | BOLL |
| MIR1-1 | ASH2L |
| VEGFB | SEMA7A |
| ZMYND8 | ZSCAN4 |
| PHLPP1 | CSDE1 |
| LINC00659 | PPIB |
| MEF2A | SEPHS1 |
| KDM7A | LIN7C |
| FOXO4 | CSF3R |
| LAPTM4B | PISRT1 |
| USP15 | SMARCC2 |
| TLR3 | NEK11 |
| LPCAT1 | JTB |
| VPS9D1-AS1 | NDUFAF7 |
| ZYX | MIR92B |
| GUCY2C | AP1M1 |
| GATA6-AS1 | ZBTB18 |
| TGFB1I1 | CAPZA2 |
| LGALS3BP | LEF1 |
| VDAC2 | SNRPA1 |
| TLE1 | CBX6 |
| STAT2 | MIR190B |
| IGF2BP2 | NSUN4 |
| STC1 | NR2C2 |
| CD63 | MIR634 |
| CCN3 | RNY3 |
| MIR411 | SCARNA14 |
| MIR101-2 | NHERF2 |
| DSPP | MAGI3 |
| LOC111099027 | OVOL1 |
| LOC111099028 | USP21 |
| FGB | SPNS1 |
| VRK1 | DCLK1 |
| SLC39A4 | MFAP1 |
| MYOF | SNW1 |
| GLRX | CEP131 |
| DNAH10 | RNASET2 |
| FSIP2 | SIX4 |
| NUDT19 | MIR661 |
| PALM3 | MIR452 |
| ZNF391 | MIR582 |
| C2CD4C | EFNB1 |
| CCDC168 | PAFAH2 |
| CEP295NL | VEGFD |
| TCF24 | CALCOCO2 |
| TDRD15 | FKBP2 |
| HOXB-AS4 | LINC00336 |
| CHMP4BP1 | ENOPH1 |
| LINC00645 | CLK4 |
| TMCC1-DT | RPL36AL |
| MIR4694 | PAXIP1-DT |
| MIR1587 | MIR199A2 |
| PSEN1 | MIR545 |
| TNFRSF10C | EIF4G2 |
| CPEB1 | MIR29B2 |
| SPIN1 | FGF10 |
| FGA | ANXA4 |
| MYCBP2 | LINC00339 |
| HIPK1 | ALKBH1 |
| CARMN | HOXC-AS3 |
| MIR654 | ARIH1 |
| GAB2 | HIVEP2 |
| NFIC | CRELD2 |
| UCN2 | MCMBP |
| RPS6 | MIR497 |
| SLC40A1 | ISG20 |
| PELATON | NCAM1 |
| BFSP2 | FGF8 |
| CGN | ESRP2 |
| MIR770 | IPO8 |
| ATF4 | NDUFA7 |
| ATF5 | KRT10 |
| PHLPP2 | BASP1 |
| SPINK13 | TRIM24 |
| CD34 | DBN1 |
| EGLN3 | OBSCN |
| SLC3A2 | ADAM12 |
| NTF3 | JPT1 |
| TRPC1 | PEG3 |
| CAD | MIR641 |
| HNRNPA2B1 | CORO1B |
| HFE | EDN3 |
| IPO4 | MT1E |
| KLRK1 | HGFAC |
| GAL | ELMO1 |
| KL | UACA |
| ADGRL2 | CENPE |
| SOHLH2 | BCAR1 |
| SYF2 | ITGB5 |
| FBN2 | HAPLN1 |
| PKLR | APOBEC3A |
| LINC00461 | MIR92A2 |
| BTG3 | CORO1C |
| MAML1 | RBM28 |
| BCAT1 | SNORD20 |
| ACVRL1 | CCL19 |
| KLC1 | DCAF13 |
| TUBB6 | AK6 |
| TMEFF2 | MAP4 |
| TBP | DVL1 |
| ADCYAP1 | EBAG9 |
| KMT5C | FAHD2A |
| MIR92B | NSFL1C |
| EPB41L5 | TTF1 |
| PLPP2 | PTPN14 |
| BRD7 | HMBOX1 |
| EGFR-AS1 | RCBTB1 |
| CAMP | MIR502 |
| MIR147B | NELL1 |
| MIR376A1 | TFAP2C |
| DSG2 | MIR1290 |
| KCNQ1 | TMX3 |
| PTGER2 | MIR518B |
| MYO1D-DT | PCA3 |
| COL1A2 | OLIG2 |
| ALCAM | NFE2 |
| NETO2 | ESF1 |
| RHBDF1 | PSMD5 |
| PSPC1 | SLTM |
| RPL7 | TXLNG |
| APOB | GRPEL1 |
| CXCL13 | ATAD2 |
| ZFPM2 | SFRP1 |
| NEDD4L | RAB26 |
| STK38 | MIR17HG |
| HBP1 | NIPSNAP1 |
| DUSP13B | HEY2 |
| HOXB-AS1 | TRIP6 |
| VCAM1 | SETDB2 |
| RPL7A | MSC-AS1 |
| RPS13 | SEPTIN11 |
| RN7SL1 | CIP2A |
| TOLLIP | CEP43 |
| TRIM59 | LINC01599 |
| MEF2B | LINC01588 |
| PKN1 | DDX3Y |
| SOX11 | DLK2 |
| CDK8 | ZMYND8 |
| REG3A | NKX2-8 |
| RPS8 | SLC22A16 |
| MIR520D | CDX4 |
| ROS1 | IL27 |
| PER2 | WDR3 |
| MIR612 | SULF1 |
| CADPS | LBX1 |
| AKR1C1 | HSPBP1 |
| AKAP4 | TRL-TAG1-1 |
| RNF111 | TAF6 |
| USF1 | LCP2 |
| MZF1 | CXCL1 |
| MAP3K20-AS1 | MT1F |
| CLDN10 | EOGT |
| ACVR1B | NELFE |
| EMP3 | DHX32 |
| MID1 | CD3D |
| TERF1 | PAX3 |
| SALL1 | UBE2C |
| TNFAIP8L1 | GTF2H4 |
| MSI1 | CCNT1 |
| PICK1 | GATD3 |
| CXCL11 | CLIP1 |
| BIRC3 | SH3GL2 |
| MIR3148 | NELFA |
| EIF4A1 | AKAP12 |
| ARHGAP1 | MIR493 |
| NUCB1 | ADGRA1 |
| NANOGP8 | NEBL |
| LINC01857 | MAGEA3 |
| EWSR1 | RBM25 |
| CAST | PITX2 |
| RAC2 | ACTBL2 |
| TNFSF13B | PSPC1 |
| KDM4C | RNF123 |
| ACTC1 | DAZL |
| CCL3 | SDAD1 |
| STAT4 | LNCNEF |
| MAP4 | MIR487A |
| LAYN | LINC00673 |
| CCDC88A | LMO3 |
| RAB23 | TXNDC12 |
| TRIB3 | ERGIC1 |
| MAOB | JUND |
| MAPRE1 | BUB3 |
| CYP27B1 | HDHD3 |
| MIR767 | ANXA7 |
| MIR3662 | FAIM |
| BAMBI | ACTR2 |
| RBMX | LPAR3 |
| CXCL14 | AMELX |
| SEMA7A | SNORA1 |
| KIN | MIR138-1 |
| APBB1 | USP50 |
| E2F7 | MIR421 |
| MIR760 | PRPF38A |
| SIK1 | PLAC8 |
| RUVBL1 | RAB19 |
| CLDN14 | COA4 |
| FAT4 | MT1B |
| GNA13 | PLXNB2 |
| PYGB | IFT140 |
| ALDH2 | TUBGCP3 |
| LINC00612 | ERGIC3 |
| MIR561 | AHSA1 |
| ATP2A2 | KLHL20 |
| HLA-F-AS1 | EPB41L2 |
| MIR6736 | DMAP1 |
| ID4 | MIR542 |
| CYP24A1 | CETN2 |
| GNAS | PDLIM7 |
| DDAH1 | TMED7 |
| INPPL1 | SAP30BP |
| CLOCK | LINC01094 |
| ASPH | MIR3591 |
| SHOX2 | ANXA3 |
| HOXC-AS3 | SNRPB |
| AMOT | IRF6 |
| PA2G4 | WASF1 |
| PLCG1 | LOC111099027 |
| PTGES | H1-5 |
| MTUS1 | EMC10 |
| MIR218-2 | SMU1 |
| F2RL3 | FOXF2 |
| MIR466 | CTDSPL2 |
| FUT3 | GPX8 |
| HOXB5 | AP4M1 |
| EMD | MIR520D |
| REN | TBC1D7 |
| KLK10 | MT1M |
| TTYH3 | THUMPD2 |
| PCDH17 | KCNA3 |
| LOC117134604 | CREB3 |
| LOC117134605 | SAV1 |
| LOC117134606 | HNF4A-AS1 |
| LOC117134607 | WNT7A |
| LOC117134608 | MIR4668 |
| LOC117134611 | TMEM14C |
| LOC117135104 | LOC111099028 |
| LOC117135105 | MIR449C |
| LOC117135106 | LINC01234 |
| DACT2 | MIR3157 |
| SCD | NDN |
| FLI1 | SNHG10 |
| PHF21A | HPCAL1 |
| AMOTL1 | GPNMB |
| SSX2 | KIRREL1 |
| BMP5 | LINC00842 |
| IBSP | USP32 |
| IFNB1 | METTL2B |
| RARRES1 | TRIM22 |
| MIR564 | TBC1D1 |
| UBQLN2 | CRTC3 |
| ITGAE | DLX6-AS1 |
| PTPRZ1 | IFNLR1 |
| HNRNPF | UBXN4 |
| ADAM15 | EMC2 |
| HRH4 | VTRNA2-1 |
| LINC01150 | MT1H |
| NOD2 | EIF3H |
| CCL4 | LEMD2 |
| ASIC1 | TCOF1 |
| SESN2 | ARPC4 |
| MIR656 | MIR137HG |
| TRPM8 | WDR76 |
| IRF8 | CD247 |
| CDC5L | GNL1 |
| CBX3 | LUC7L3 |
| RNU1-1 | CLUH |
| RPL9 | MBNL2 |
| CBL | COLEC11 |
| KISS1R | NFATC2 |
| MIR210HG | CD209 |
| NUSAP1 | ZYX |
| CCL26 | EN1 |
| GJB2 | PMS1 |
| NCBP1 | ANLN |
| MT-TG | RYK |
| MAP1B | UBP1 |
| C3AR1 | GTF3C1 |
| MEIS2 | MYL3 |
| SCARNA22 | TRIM17 |
| RAD23A | ENSA |
| MICALL2 | LOC111674477 |
| DSCAM-AS1 | ETV1 |
| PPARD | DNAH2 |
| ACSL4 | ITIH5 |
| IGFBP4 | LTBP2 |
| BAG1 | SELENOF |
| RNU1-4 | TBC1D25 |
| RNVU1-18 | SMAD1 |
| RNU1-2 | MIR767 |
| RNU1-3 | ITGA1 |
| RNVU1-29 | MIR1973 |
| LOC124904613 | IL21 |
| TSPAN8 | CTNNBL1 |
| NLK | AFG2A |
| MIR4306 | SPATA7 |
| MIR625 | AP1S2 |
| DLST | DKK3 |
| FLNC | IFIT3 |
| TRIP12 | BST2 |
| MFN2 | ISL2 |
| SGMS1 | TBR1 |
| CMTM8 | GFI1B |
| ERGIC3 | SKA2 |
| SCAI | LINC01537 |
| RETNLB | TIMM9 |
| MIR4286 | MIR484 |
| RNA18SN1 | RAB5B |
| MIR432 | NOL10 |
| EYA1 | MNT |
| WARS1 | VIL1 |
| MTHFD1L | MIR129-2 |
| EPHB6 | RCOR2 |
| FLG | SMARCD2 |
| PDE4D | MIR520E |
| TAOK1 | PHF7 |
| MIR208A | DSC2 |
| FOXD1 | PPP1R13B-DT |
| DCP1A | AP3B2 |
| HMG20A | PTK7 |
| OTUB1 | P3H2 |
| CEP70 | MYB-AS1 |
| CEP76 | TRL-AAG2-3 |
| GPX4 | FRZB |
| ERVW-1 | C10orf90 |
| MT-RNR2 | CDC6 |
| TMPRSS3 | EEF1A1P5 |
| YBX2 | SDCBP |
| FMR1-AS1 | HELB |
| TRIM11 | SCRT1 |
| HOXC13 | PALM |
| NET1 | MIR802 |
| MYL6B | STK38 |
| PDZD8 | STAG3L5P-PVRIG2P-PILRB |
| TXNDC9 | BAIAP2 |
| SLC22A18AS | ENSG00000285959 |
| CD27-AS1 | PPIH |
| NALT1 | TNFRSF10D |
| SERPINB12 | MANCR |
| PDIA3P1 | SNORA66 |
| CADM2 | TBX6 |
| MIR4516 | GPR183 |
| VCPIP1 | MIR766 |
| GRHL2-DT | SNORD81 |
| MIR1225 | ETS2 |
| PAPPA-AS1 | ENSG00000228741 |
| CST5 | EPB41L4A-DT |
| SMC2 | KRT8P3 |
| SLC16A4 | LINC01638 |
| CDCP1 | LINC00365 |
| ECT2 | MIR337 |
| DIS3 | ADORA3 |
| BOP1 | GJC1 |
| SNORD25 | MIR3667HG |
| P2RY2 | MIR3189 |
| CYLD | GP9 |
| FMR1 | LINC00520 |
| USP26 | SETD7 |
| UNC119 | ZHX2 |
| GLRX3 | SNORD73A |
| CDH22 | RCC2-AS1 |
| DTX3 | GEMIN5 |
| YDJC | RUNX1T1 |
| MIR579 | USP29 |
| MIR4723 | MIR4516 |
| MIR890 | TREM1 |
| COL12A1 | MX2 |
| POU2AF3 | HNRNPUL2 |
| ABCC4 | WDR82 |
| GFI1 | CCDC86 |
| MPO | TRL-AAG2-4 |
| GHR | TOX |
| RPL14 | MIR4286 |
| POLR2C | MAP3K2 |
| NCOA5 | NSD3 |
| PRPF19 | GADD45GIP1 |
| CAV2 | H1-3 |
| TAB1 | MSLN |
| GRK2 | MBD1 |
| MED1 | TICAM1 |
| MIR628 | DPF2 |
| PAX9 | LGALS8 |
| GART | PAK4 |
| NKX3-1 | EPC1 |
| THORLNC | EPC2 |
| KIF2C | TRL-AAG2-1 |
| PITPNM3 | TRL-AAG2-2 |
| NDUFA13 | HCG11 |
| KDM3A | ID1 |
| ADAMTS9 | POU2F2 |
| LINC02982 | EIF3J-DT |
| SULF1 | MIR3120 |
| SVIL | HOXA10 |
| ITIH2 | HDAC8 |
| SCRT2 | NOX5 |
| ADARB2-AS1 | LOC108281177 |
| LINC00205 | DLL1 |
| DOCK9-DT | MMP15 |
| LINC02321 | PTPN6 |
| LINC01871 | YES1 |
| MIR4500 | AAMDC |
| lnc-DYNC2H1-4 | MIR615 |
| HSPA1L | PPM1D |
| TNFRSF1A | OCIAD2 |
| XRCC6 | MAST1 |
| GJA3 | SLC38A5 |
| HSPH1 | TRK-TTT3-5 |
| DHX15 | TRK-TTT3-1 |
| ITGA1 | TRK-TTT3-2 |
| UBE2L3 | SGO1 |
| LINC01094 | KPNA4 |
| TTK | FLT3LG |
| ENKUR | MIR588 |
| PLAC1 | AM263179 |
| MIR433 | MIR208A |
| MIR329-1 | TRK-TTT3-4 |
| IPO5 | TRK-TTT3-3 |
| PKN2 | SMPDL3A |
| ELP1 | CCDC85C |
| MUC12 | IRAK2 |
| IL2RG | CRIP2 |
| ADRA1A | TNFAIP8L1 |
| DNAJB11 | SRCAP |
| KPNA4 | CDCA5 |
| KCNK15-AS1 | AP008439 |
| LINC01546 | LMO7 |
| LINC02620 | TMX2 |
| DMP1 | MMGT1 |
| CEACAM7 | POTEF |
| NID1 | RPLP0P6 |
| SLC25A13 | MIR296 |
| PIEZO1 | PTBP2 |
| RPS6KA3 | ASPN |
| SMC4 | RCN3 |
| CRYAA | MXRA7 |
| BRCC3 | RNF43 |
| PTGDS | MEX3A |
| MBD2 | AKT1S1 |
| LRP1 | TBX2 |
| IL6ST | MIR378E |
| THBS2 | SDF4 |
| IQGAP2 | SRGN |
| LINC01852 | CTNNA3 |
| UICLM | EFHC1 |
| LINC01140 | FCAR |
| ASPN | WASF3 |
| SRSF3 | VPS26B |
| RAP1A | MIR449B |
| TRAF3 | SNORD104 |
| UBA2 | DDX56 |
| HTR1B | LILRB2 |
| PPM1B | CHID1 |
| ARHGAP19-SLIT1 | SFXN2 |
| HMGCR | ESCO2 |
| APLNR | DAZ2 |
| FAM225A | MAP1LC3C |
| PCDH10 | NFIL3 |
| GRHL3 | HEXIM1 |
| KLF9 | ATG4B |
| LELP1 | EAF2 |
| PARP3 | POTEKP |
| SOX18 | FAM120A |
| PWAR5 | KPNA6 |
| UPP1 | MIR1247 |
| ILF2 | PDZD2 |
| PTH1R | LRIG1 |
| IVL | IL3RA |
| CLASP2 | HERC5 |
| MIR634 | ODC1-DT |
| DVL1 | TP53BP2 |
| FGF18 | MIR362 |
| PICSAR | MIR99AHG |
| LGMN | ASXL3 |
| BCL2L14 | BCLAF1 |
| STK3 | AF346985-001 |
| SIK2 | AF346985-002 |
| MIR582 | AF346985-003 |
| CDK10 | KCNK15-AS1 |
| MASTL | LINC01260 |
| MIR664A | ENSG00000132832 |
| UBE3A | PSMA3-AS1 |
| JMJD6 | PCGEM1 |
| RAB11FIP2 | DSTN |
| HAGLROS | SLC17A7 |
| ACLY | IDH1-AS1 |
| KLF11 | EIF3G |
| STON2 | TSR1 |
| MIR1248 | FUNDC1 |
| DUSP1 | ZNF423 |
| PEX13 | G3BP2 |
| PCK2 | GRSF1 |
| PFN1 | ST14 |
| ABCC2 | DLX4 |
| VDAC3 | RSPO1 |
| OBSCN | GPS1 |
| TIMD4 | BATF |
| MIR1268A | FZD3 |
| NONO | PUM1 |
| NECTIN4 | MIR454 |
| EFNA1 | ITGB2-AS1 |
| ZNF224 | SDF2 |
| PHF19 | TOLLIP |
| FAM30A | CREB5 |
| MIR626 | DUX4 |
| MIR449C | TRERNA1 |
| MIR552 | MIR320D1 |
| DIAPH1 | C1orf198 |
| VAV1 | LINC00964 |
| TRIM25 | CPEB4 |
| AFF4 | RARRES1 |
| STK38L | CDX1 |
| ASAP1 | MAGEA1 |
| EIF5B | ICOS |
| EBNA1BP2 | UCHL3 |
| NOP2 | STK35 |
| TNPO1 | LINC00857 |
| CLCA1 | IL12RB2 |
| ANTXR2 | NUMB |
| MAP1LC3B | PPM1A |
| MIR1293 | CUX1 |
| EFNB2 | EBI3 |
| FMN1 | LOC106728418 |
| PRR14 | MIR519A1 |
| MIR4755 | ENSG00000285402 |
| DPYSL3 | CREBZF |
| KCNH1 | LRCH3 |
| ING2 | HEBP1 |
| SEPTIN2 | LEFTY1 |
| ITGAL | VIM-AS1 |
| PRKCQ | RNA5-8SN1 |
| COX4I2 | RNA5-8SN2 |
| MMP28 | RNA5-8SN3 |
| REPS2 | RNA5-8SP10 |
| AKAP12 | LOC124907114 |
| EEF1A1 | LOC124907115 |
| XPOT | LOC124907116 |
| MAP3K8 | LOC124907117 |
| DLG4 | LOC124907118 |
| C10orf55 | LOC124907119 |
| DOCK2 | LOC124907120 |
| KAT8 | LOC124907121 |
| KCTD12 | LOC124907122 |
| TSG101 | LOC124907123 |
| NR4A3 | LOC124907124 |
| WIPF1 | LOC124907125 |
| MINCR | LOC124907126 |
| ITGA7 | LOC124907127 |
| RING1 | LOC124907128 |
| RYBP | LOC124907129 |
| SPAG5 | LOC124907130 |
| HSPA2 | LOC124907131 |
| FOXD1-AS1 | LOC124907132 |
| PTTG2 | LOC124907133 |
| PRMT2 | LOC124907134 |
| TMEM100 | LOC124907135 |
| IL1RN | LOC124907136 |
| UBE2E1 | LOC124907137 |
| ARHGAP15 | LOC124907138 |
| ELF3-AS1 | LOC124907139 |
| SERBP1 | LOC124907140 |
| TICRR | LOC124907141 |
| CX3CL1 | LOC124907142 |
| MEST | LOC124907143 |
| SFTPB | LOC124907144 |
| RAB31 | LOC124907145 |
| AKR1C2 | LOC124907146 |
| PROP1 | LOC124907147 |
| PPM1A | LOC124907148 |
| AHSG | LOC124907149 |
| INPP5D | LOC124907150 |
| KDM4A | LOC124907151 |
| PRMT7 | LOC124907152 |
| BCL2L2 | LOC124907153 |
| KIDINS220 | LOC124907154 |
| PFDN1 | LOC124907155 |
| SGPP1 | LOC124907160 |
| RRP15 | LOC124907172 |
| MUCL1 | LOC124907183 |
| MIR504 | LOC124907194 |
| TP73-AS1 | LOC124907205 |
| SETD7 | LOC124907216 |
| HNRNPC | LOC124907227 |
| UBE2J2 | LOC124907238 |
| MIR5703 | LOC124907248 |
| FBLN2 | LOC124907258 |
| COL6A1 | LOC124907269 |
| NEFL | LOC124907280 |
| EEF1A2 | LOC124907291 |
| FOSL2 | LOC124907302 |
| LIMK2 | LOC124907313 |
| CHRNA5 | LOC124907324 |
| REST | LOC124907327 |
| SSRP1 | LOC124907328 |
| DOT1L | LOC124907329 |
| IFIT3 | LOC124907330 |
| PINX1 | LOC124907331 |
| ABCB5 | LOC124907332 |
| TPT1 | LOC124907333 |
| PAFAH1B2 | LOC124907334 |
| CERS6 | LOC124907335 |
| GINS2 | LOC124907336 |
| SKA1 | LOC124907337 |
| ZFP64 | LOC124907338 |
| SCML2 | LOC124907339 |
| HTN1 | LOC124907340 |
| RADIL | LOC124907341 |
| MIR3196 | LOC124907342 |
| PDCD1LG2 | LOC124907343 |
| TCOF1 | LOC124907439 |
| CXCL2 | LOC124907440 |
| MIR593 | LOC124907450 |
| MT-RNR1 | LOC124907458 |
| PRDM1 | LOC124907467 |
| HADHA | LOC124907475 |
| AGTR2 | LOC124907476 |
| LINC00667 | LOC124907477 |
| ARHGDIA | LOC124907478 |
| CPXM2 | LOC124907479 |
| PLEK2 | LOC124907480 |
| AIMP1 | LOC124907481 |
| PARP2 | LOC124907482 |
| RANBP2 | LOC124907483 |
| KTN1 | LOC124907484 |
| TFCP2L1 | LOC124907572 |
| EPHB3 | LOC124907573 |
| PDCD6IP | LOC124907574 |
| CFH | LOC124907575 |
| EIF4G2 | LOC124907576 |
| OTX2 | LOC124907577 |
| SQLE | LOC124907578 |
| OGN | LOC124907579 |
| FGD5 | LOC124907580 |
| SCAND1 | LOC124907581 |
| ARRDC3 | LOC124907584 |
| GUCD1 | LOC124907585 |
| PAX8-AS1 | LOC124907586 |
| MIR935 | LOC124907587 |
| FAM3D-AS1 | LOC124907588 |
| MYOG | LOC124907589 |
| IGFBP1 | LOC124907590 |
| GSTO1 | LOC124907591 |
| SNAI3 | LOC124907592 |
| STIP1 | LOC124907593 |
| MIR492 | LOC124907594 |
| KRT6A | LOC124907595 |
| AGL | LOC124907596 |
| PPP1R8 | LOC124907597 |
| TCIRG1 | LOC124907598 |
| MT3 | LOC124907600 |
| MIR376B | LOC124907611 |
| MIR516B1 | LOC124907622 |
| LSINCT5 | LOC124907633 |
| HTR1D | LOC124907644 |
| MYH10 | LOC124907655 |
| WASF2 | LOC124907662 |
| EIF4B | LOC124907673 |
| IFIT1 | LOC124907684 |
| EHD2 | LOC124907694 |
| SOX12 | LOC124907705 |
| ZFP91-CNTF | LOC124907709 |
| MIR520F | LOC124907710 |
| HK1 | LOC124907711 |
| SAA1 | LOC124907712 |
| BANP | LOC124907713 |
| KIF13B | LOC124907714 |
| SLCO4C1 | LOC124907715 |
| TENM1 | LOC124907716 |
| STXBP5-AS1 | LOC124907717 |
| MIR380 | LOC124907718 |
| NRSN2-AS1 | LOC124907719 |
| MIR4775 | LOC124907720 |
| XPNPEP2 | LOC124907721 |
| SPIDR | LOC124908237 |
| TLR5 | LOC124908238 |
| MTMR2 | LOC124908239 |
| SLIT3 | LOC124908240 |
| ARTN | LOC124908241 |
| GALNT12 | LOC124908242 |
| MYO6 | LOC124908243 |
| KLF1 | LOC124908244 |
| PGAM1 | LOC124908245 |
| CHD1L | LOC124908246 |
| NKAP | LOC124908247 |
| GBA1 | LOC124908248 |
| KLK5 | LOC124908249 |
| MIR525 | LOC124908257 |
| MIR3619 | LOC124908268 |
| MIR520B | LOC124908278 |
| PAXIP1-DT | LOC124908289 |
| PRAME | LOC124908300 |
| MBNL1 | LOC124908310 |
| CD58 | LOC124908316 |
| TPI1 | LOC124908327 |
| CANX | LOC124908336 |
| BCL11A | LOC124908347 |
| IL12A | LOC124908358 |
| TFCP2 | LOC124908368 |
| MARCKS | LOC124908369 |
| SOX7 | LOC124908370 |
| ST3GAL1 | LOC124908371 |
| LGALS9 | LOC124908372 |
| MRC1 | LOC124908373 |
| SOX8 | LOC124908374 |
| ALAD | LOC124908375 |
| EIF3A | LOC124908376 |
| GOLGA2 | LOC124908377 |
| PLAGL1 | LOC124908378 |
| KLK8 | LOC124908379 |
| ITGB8 | LOC124908380 |
| MIR1275 | LOC124908381 |
| LNCOC1 | LOC124908382 |
| PXN-AS1 | LOC124908383 |
| IFIT2 | LOC124908384 |
| MIR484 | LOC124908385 |
| MIR383 | LOC124908386 |
| ARAP1-AS1 | LOC124908387 |
| CCT3 | LOC124908388 |
| RPS4X | LOC124908389 |
| BMP1 | LOC124908390 |
| NR1H3 | LOC124908391 |
| MFAP5 | LOC124908392 |
| NCAPH | LOC124908393 |
| PPP1R13L | LOC124908474 |
| TNFRSF12A | LOC124908494 |
| TRAIP | LOC124908504 |
| MIR3178 | LOC124908512 |
| DDB2 | LOC124908513 |
| SERPING1 | LOC124908514 |
| PF4 | LOC124908515 |
| WWC1 | LOC124908516 |
| SACS-AS1 | LOC124908517 |
| KDM4A-AS1 | LOC124908518 |
| LINC01215 | LOC124908519 |
| EPHA1 | LOC124908520 |
| DRD2 | LOC124908521 |
| STK11IP | LOC124908522 |
| TPTE2 | LOC124908523 |
| CAPZA1 | LOC124908524 |
| C4A | LOC124908525 |
| BCL3 | LOC124908527 |
| KIF20A | DDX11-AS1 |
| NEO1 | LINC02620 |
| SOCS5 | SPECC1L |
| MLLT3 | CNTN5 |
| CCL8 | ALYREF |
| PRRX2 | GAB2 |
| CCDC26 | SENP7 |
| MIR940 | MAPT-AS1 |
| ILF3-DT | TAX1BP1 |
| SYCP1 | CCN4 |
| P2RX7 | MIR449A |
| CCAR2 | MIR450B |
| RBM14 | GJA9-MYCBP |
| FDPS | TRN-GTT4-1 |
| MED12 | PRSS50 |
| CXCR5 | CEACAM3 |
| HNRNPL | MIR655 |
| RAB14 | SKA3 |
| SNORD41 | E2F2 |
| SI | IFI35 |
| EPDR1 | MEX3C |
| RNY4 | LNC-LBCS |
| SYNPO | IGKV2-28 |
| USP44 | ZBTB2 |
| CRIM1 | MIR135A2 |
| CLDND1 | MYL12A |
| KIF3C | KPRP |
| RAB3D | TMPRSS11B |
| VGLL4 | CELSR3 |
| STK32A | GRIN2D |
| MB21D2 | DNAJC11 |
| HCFC1R1 | MCTS1 |
| SPANXA1 | ZBTB10 |
| VTRNA2-1 | LINC00458 |
| AGAP2-AS1 | DYRK3 |
| MSBP1 | GLOD4 |
| KCNJ2 | SLC5A12 |
| KAT7 | GPRC5A |
| SIAH1 | EMC7 |
| POLR2B | KRT16 |
| FOXE1 | STON2 |
| CPQ | ING3 |
| NELFE | VPS72 |
| CEBPD | HMCES |
| INSIG2 | ENSG00000277444 |
| MRPS22 | SAMMSON |
| FUT6 | GPR31 |
| MIR584 | FHL3 |
| UCN | TMEM109 |
| SNIP1 | IKZF2 |
| ROMO1 | VPS39 |
| RPS3 | ZFR |
| RIPK2 | RASSF1-AS1 |
| GFPT1 | DNAJC2 |
| CMA1 | IRF8 |
| APOA4 | NR4A1AS |
| HPX | MPDZ |
| PTP4A2 | RAB11FIP5 |
| HPR | STX7 |
| ECD | STX6 |
| PHLDB2 | MLLT3 |
| ZNF703 | MHRT |
| LMNB2 | MMP20 |
| LIFR | PCNP |
| SH3PXD2A | PRKCQ-AS1 |
| ALX4 | HSPA4L |
| GCNT3 | NRBF2 |
| LAPTM5 | MIR4741 |
| GALNT8 | MIR448 |
| RFLNA | CEBPG |
| SNHG11 | ANKRD34C |
| LINC00977 | CIAPIN1 |
| LINC00976 | SP100 |
| LINC01260 | H2AB1 |
| MIR610 | SLAMF7 |
| ENSG00000132832 | LRWD1 |
| RAPGEF3 | LOC102724159 |
| INPP4B | MIR665 |
| AQP2 | DIAPH2 |
| C1QBP | ELMOD2 |
| GALNT3 | CEP78 |
| CCR4 | TADA1 |
| HACE1 | MIR422A |
| EIF3C | MIR1269A |
| CFB | RPP25 |
| MYLK2 | SIGLEC1 |
| EIF2AK4 | MIR663B |
| POLI | PRDM1 |
| P3H1 | AMOTL2 |
| APLN | KLK10 |
| MIR1199 | MNX1 |
| RBP1 | WNT2 |
| ALPL | TRIM33 |
| HDAC11 | CD180 |
| XRCC4 | MYCNUT |
| SUV39H1 | TBX21 |
| SERPINE2 | HEY1 |
| SOSTDC1 | ZIC1 |
| GBP1 | PPP1R9B |
| ASPM | MLANA |
| MIR190B | ROR1 |
| RYK | SNORD46 |
| CCDC43 | PHF8 |
| SNHG29 | CLDN7 |
| MIR2355 | CREG1 |
| LINC00426 | CD300LF |
| MIR1306 | TBC1D17 |
| LETR1 | ZFHX4 |
| LINC00861 | MIR190A |
| ID2-AS1 | CXXC1 |
| LINC00278 | KBTBD4 |
| TMEM92-AS1 | PRSS2 |
| TBILA | IGKV2D-29 |
| MIR2053 | ROR2 |
| TACR3-AS1 | LRRC7 |
| EHF | LINC02982 |
| DOCK1 | ZSCAN10 |
| CD70 | CEND1 |
| AQP9 | TOMM5 |
| KLF10 | TOMM6 |
| MOB1A | PA2G4 |
| FSTL5 | MAGEC2 |
| LGALS7 | PELI1 |
| CDX1 | BHLHE41 |
| MIR9-1HG | TNMD |
| HYOU1 | LINC00462 |
| FRK | SREBF2-AS1 |
| TLE3 | MIR4739 |
| DPP9 | NPTN |
| UBE4A | TRS-TGA2-1 |
| FMNL1 | ACTR6 |
| HTRA3 | MRC2 |
| RNF6 | MLLT11 |
| TNKS1BP1 | NIPSNAP3A |
| ALKBH2 | PTRHD1 |
| CENPA | FOSB |
| SIX3 | TRIP10 |
| SLC39A10 | LINC01503 |
| RHOU | SNORD102 |
| CCR1 | SUPT3H |
| CACNA2D3 | KRT6A |
| GLIS1 | RGS4 |
| SLC29A1 | POU3F4 |
| GC | ESM1 |
| PTPRK | CLEC4C |
| BRD2 | NKX2-2 |
| DPEP1 | CD2 |
| CX3CR1 | MXD1 |
| CYFIP2 | MIR579 |
| MTO1 | LOC107963955 |
| ATG3 | NEDD9 |
| MEOX1 | RGS6 |
| NUAK2 | DOCK10 |
| SERPINA4 | TMEM198B |
| SLC30A8 | DUSP6 |
| B3GALT5 | FZD7 |
| TMEM126A | FAM234A |
| RASAL1 | LINC01060 |
| FRAT1 | SOX17 |
| MNT | TEAD4 |
| C1orf116 | PAX7 |
| SCX | LOC107980440 |
| SPANXB1 | JPH2 |
| PAGE2 | CBFA2T2 |
| MIR601 | MIR654 |
| MIR3648-1 | OTUB2 |
| MIR3648-2 | FUBP1 |
| PRPF8 | PCF11 |
| RAB13 | HMGB2 |
| LNC-LBCS | LRG1 |
| PTRH2 | MIR320B1 |
| PAPPA | MIR320B2 |
| SPARCL1 | PADI1 |
| SYT11 | DLEC1 |
| CCL25 | GLMP |
| ANGPTL2 | PCIF1 |
| SKA2 | TRG-GCC2-6 |
| MLLT11 | CNN1 |
| RNF187 | VPS29 |
| LINC00324 | HLF |
| LINC01093 | TARID |
| ETV6 | LINC00707 |
| PPIB | ENSG00000285743 |
| FUT1 | ENSG00000285845 |
| TIMP4 | MICOS10 |
| SIPA1L3 | EIF4G3 |
| LINC01013 | MIR4284 |
| ADAM28 | MYO1B |
| EPHA7 | RBM47 |
| A2M | DGCR8 |
| HMGB2 | TBC1D2 |
| C4BPA | KRT6B |
| RAB4A | ARHGEF12 |
| FGL2 | KCNMB1 |
| ITIH1 | RFTN1 |
| LMO4 | SNORA75 |
| A1BG | GSEC |
| EMB | PCGF6 |
| HAS1 | BATF3 |
| PIR | MAGEA4 |
| RETREG1 | ENSG00000285517 |
| COPS7A | EPHA5 |
| DMXL2 | GREB1 |
| ZNF746 | DAAM1 |
| SYT13 | PDCD1LG2 |
| FAM83B | MIR676 |
| MMD | MIR4454 |
| PDZK1IP1 | MIR548AH |
| VOPP1 | TRG-CCC2-2 |
| HSP90AA2P | TRG-TCC1-1 |
| SNORD94 | MIR5701-1 |
| RB1-DT | MIR5701-2 |
| ABALON | TRG-CCC2-1 |
| MIR5100 | TRG-GCC2-1 |
| TRL-TAG1-1 | TRG-GCC2-3 |
| TRS-TGA2-1 | MIR5701-3 |
| FAM201A | TRG-GCC2-2 |
| ILF3 | TRG-GCC2-4 |
| FBL | TRG-GCC2-5 |
| EN2 | lnc-HLTF-5 |
| CAV3 | LOC117134604 |
| UCP1 | LOC117134605 |
| MAPKAPK2 | LOC117134606 |
| AZGP1 | LOC117134607 |
| CTNND2 | LOC117134608 |
| NR5A1 | LOC117134611 |
| LACTB | LOC117135104 |
| ST7-OT3 | LOC117135105 |
| LTBP2 | LOC117135106 |
| SLC1A5 | MYCL |
| TFPI2 | AGAP2 |
| FAM172A | PXK |
| EPPK1 | STRN3 |
| NR2F2-AS1 | CDR2 |
| LOC111162621 | MIR339 |
| UVRAG | CDK2AP1 |
| MTHFD2 | SNORD47 |
| MT-TP | MIR320D2 |
| CAPN9 | MIR2052 |
| EFNA2 | THRIL |
| CDK5R2 | PATJ |
| ADGRL4 | EP400 |
| LMO3 | KANSL2 |
| CTDSPL2 | KRBOX4 |
| EI24 | MIR486-2 |
| MEGF6 | CCR8 |
| SCEL | BCL2L13 |
| ZNF532 | CUTA |
| LMO7DN | MIR6852 |
| MIR517C | KANSL1 |
| MIR576 | KIF15 |
| MIR875 | TYROBP |
| SH3PXD2A-AS1 | HIF1A-AS2 |
| LINC01233 | MIR1908 |
| LINC01569 | RN7SK |
| TRIM63 | MYO15A |
| RCAN1 | EMC8 |
| PHLDA3 | SH3BGRL3 |
| CD164 | C15orf48 |
| MORF4L2 | NIFK-AS1 |
| NPHS1 | MIR3182 |
| RGS16 | EZH1 |
| CPSF6 | MXI1 |
| MIR517A | FZD9 |
| OGDHL | AMOTL1 |
| EDIL3 | SMARCAD1 |
| MIR513A1 | KCNQ1-AS1 |
| LINC02418 | TEAD3 |
| ENSG00000227083 | FAM24B-CUZD1 |
| SHMT2 | LACTB2 |
| RPL18 | TMX4 |
| SYNCRIP | LYRM2 |
| MAP1LC3A | MIR497HG |
| CKAP4 | LINC02223 |
| MIR1-2 | ENSG00000267047 |
| INTS4 | DACOR1 |
| MIR519A1 | TBC1D10B |
| CCT4 | ZNF407-AS1 |
| SRSF2 | TBX18 |
| MTHFD1 | EWSAT1 |
| NPEPPS | NFE2L3 |
| DCAF13 | TDGF1 |
| SMAD9 | PALS1 |
| ELOVL5 | CD207 |
| KCNN3 | KRT6C |
| TRAF7 | MIR222HG |
| CASQ2 | CD70 |
| NSF | PWRN1 |
| TNFAIP2 | POU2F3 |
| MIR1231 | ACKR3 |
| MIR4767 | WNT3A |
| MIR1255B1 | VPS9D1-AS1 |
| MIR1471 | HOXB4 |
| MIR3937 | SUGP2 |
| MIR4632 | AJUBA |
| MIR1255B2 | PRRX2 |
| MIR3135B | SH3BGRL |
| MIR5003 | TRIAP1 |
| MIR7110 | TTYH3 |
| MIR6802 | MGARP |
| LOC108942766 | HSP90B2P |
| PCBP2 | SNHG19 |
| G6PC1 | LINC00996 |
| HNRNPD | ITGB7 |
| SP7 | POLH |
| LINC01436 | TMOD3 |
| DUXAP8 | ARHGEF11 |
| SFRP5 | NPM3 |
| IPO9 | FAM83A |
| FUBP1 | SPIB |
| MIR4756 | MIR3178 |
| ROBO3 | QNG1 |
| MIR1249 | ONECUT2 |
| LMCD1-AS1 | ONECUT3 |
| MIR1226 | RNU6ATAC |
| MIR516B2 | MIR509-3 |
| MIR3163 | PTK6 |
| MIR888 | MIR205HG |
| RSU1P2 | NELFCD |
| ADAMTSL1 | ADGRA3 |
| RIT1 | MTA3 |
| ZNF350 | ARL6IP4 |
| CCT8 | YPEL2 |
| GTF2I | HES6 |
| NIFK-AS1 | SYPL1 |
| PTK7 | ATPAF1 |
| MIR1247 | YIPF3 |
| NUDT6 | FAM162A |
| ARMC8 | MAGEB2 |
| MIR519C | EMC3 |
| HLX | POGLUT3 |
| CERS2 | ARMCX3 |
| CAPN2 | TMEM205 |
| TCF12 | C17orf75 |
| LIPC | EVA1B |
| LAMP2 | ZNF185 |
| HNRNPR | AGPAT4-IT1 |
| H1-2 | SIPA1L3 |
| USP39 | ESRP1 |
| ALDH5A1 | KIF2A |
| HUWE1 | TBX4 |
| CBX4 | DAZ1 |
| HMGN5 | CCNE2 |
| ANGPT1 | MRPL52 |
| TOR2A | BICD1 |
| HOXC8 | RIOK3 |
| TESC | SFMBT1 |
| LINC00240 | CCN5 |
| CTPS1 | TMEM141 |
| COPA | PADI6 |
| MATR3 | ZNF407 |
| IRS4 | ERRFI1 |
| ANXA4 | FOXQ1 |
| DPYSL2 | SBF2-AS1 |
| ADRB3 | MIR4689 |
| VGF | NFIA |
| ENAM | BRD9 |
| IMPA1 | VAV3 |
| TRPC6 | PURB |
| RIGI | MIR613 |
| GARS1 | SLC22A14 |
| RPN1 | BCL7C |
| SF3B2 | NLRP2 |
| RPL17 | TARBP2 |
| U2SURP | MIR1322 |
| MAN1B1 | ITGA9 |
| SLC7A11 | ANGPTL1 |
| RAB27B | CUEDC2 |
| NMU | BEGAIN |
| RPPH1 | RSPO2 |
| MIR1227 | SLC22A18AS |
| RUNX1-IT1 | LMX1A |
| MIR664B | RGPD3 |
| EFTUD2 | MACROH2A2 |
| SETD1A | RCOR1 |
| RPS16 | GTF2A1 |
| RPL23A | LINC01093 |
| TLX1NB | HECTD2 |
| PHGDH | OXR1 |
| RPL15 | UBAC1 |
| RPL21 | FUNDC2 |
| UCHL3 | BATF2 |
| TRIP10 | NANOS2 |
| ERRFI1 | PRPF31-AS1 |
| IPO7 | ORAI3 |
| SULT1A3 | SV2B |
| BGN | PDS5A |
| GPLD1 | MIR34B |
| PUM1 | ECT2 |
| ITLN1 | KRT80 |
| KRT2 | BTC |
| ARHGAP27P1 | C4BPA |
| RUVBL2 | IFITM1 |
| KIFAP3 | TRPC5 |
| MIR1258 | MYL12B |
| OTUD1 | MIR365B |
| MIR3200 | CHRNE |
| LINC01605 | KIR3DL1 |
| MIR519A2 | ENSG00000289084 |
| LINC00680 | FAIM2 |
| MIR1267 | HAND1 |
| ENSG00000272316 | KLF8 |
| LINC00680-GUSBP4 | RABGAP1 |
| GLUD1 | NUDCD3 |
| ATF6 | BTF3 |
| PHKB | SUGT1 |
| RPLP0 | RABGAP1L |
| SF3A1 | CDC42EP1 |
| IARS1 | KPNA5 |
| KRT16 | ZNF445 |
| PDZK1 | ZNF267 |
| ABCF2 | SFTA1P |
| CKAP2 | H19-ICR |
| THRAP3 | CHAER1 |
| ACACA | OR10J5 |
| LETM1 | RNU2-1 |
| APOBEC3A | H1-6 |
| SIAH2 | EIF3CL |
| CMTM6 | NBAT1 |
| EPIC1 | WSPAR |
| ETV7 | JPT2 |
| APOC2 | PRICKLE1 |
| TIA1 | WNT11 |
| RBM8A | FZD2 |
| CSDE1 | SEMA3F |
| NUDT5 | ZMYND11 |
| SF3B3 | MYT1 |
| QARS1 | PITX3 |
| LYAR | TBC1D2B |
| SLC34A2 | APLF |
| CBX8 | WASHC2A |
| RBM25 | ADAT3 |
| USP42 | SNURF |
| CHASERR | MMP10 |
| MFGE8 | SNORD7 |
| ANKS1B | IL1F10 |
| RTKN | SNHG17 |
| RBM3 | MIR629 |
| TRIM65 | ARL5B |
| H6PD | SMYD1 |
| RPS15A | TBC1D9 |
| IFITM3 | TCF25 |
| CSPP1 | TRIM15 |
| EIF3B | IGKV2-30 |
| H3-4 | IGKV2-40 |
| TAF7 | MEIS1 |
| NASP | H1-8 |
| MAPKAPK5 | WWC3 |
| PITX1 | CLDN11 |
| ARHGAP5 | TAF4B |
| PARVA | DUSP22 |
| SNRPB | CPEB2 |
| EPHA10 | OIT3 |
| PCDH8 | TBC1D16 |
| SYVN1 | ACTL6B |
| UBE2O | MIR9-1HG |
| FOXP4 | MIR520G |
| HAND1 | SPINDOC |
| ITPKA | NLRP7 |
| NAV2 | TRK-CTT2-1 |
| RLIM | CMSS1 |
| GPS1 | TTC28-AS1 |
| CDCA4 | TRK-CTT2-3 |
| LTO1 | TRK-CTT2-2 |
| ZFPM2-AS1 | TRK-CTT2-5 |
| MIR887 | TRK-CTT2-4 |
| MIR1197 | KDM4D |
| SNORD50A | MESP1 |
| MIR645 | HISLA |
| CERS6-AS1 | VPS26A |
| MYOD1 | ENSG00000271590 |
| NCF4 | ENSG00000285016 |
| EIF3F | CORO2A |
| HSPA6 | PCGF5 |
| IRF2 | PELI3 |
| NFYA | CISD3 |
| LARP7 | IGKV2-29 |
| LIPH | IGKV2D-30 |
| SPINK2 | TRBV11-2 |
| MRPS18B | IGKV2D-26 |
| UBR7 | MAFA-AS1 |
| TRIM58 | MIR513A1 |
| MIR29B2CHG | LNCTAM34A |
| GPT | MIR513A2 |
| PIGR | LEFTY2 |
| MAFG | S100A2 |
| XCL1 | SNORD42A |
| HHLA2 | LINC02990 |
| GNAS-AS1 | MYOSLID |
| YARS1 | PDZK1IP1 |
| GNE | MAPK8IP3 |
| TARID | OTX1 |
| CDR1-AS | DPPA2 |
| PCSK9 | HORMAD1 |
| PYGL | RSPO4 |
| MARS1 | TBC1D10A |
| RPS6KB2 | NUTM1 |
| DARS1 | H2AB3 |
| DDX1 | H2AB2 |
| HAVCR1 | SKA1 |
| RPL13 | LINC00205 |
| AGFG1 | LINC00242 |
| PABPC4 | PRC1 |
| BCAR3 | ARL6IP1 |
| CCT6A | MIR516A1 |
| CTCFL | CDH3 |
| FAU | ERBIN |
| NCBP2 | PHLDB2 |
| USO1 | HAND2-AS1 |
| GRWD1 | MIR153-2 |
| H2BC12 | LINC00908 |
| LMX1A | MIR1283-1 |
| EPSTI1 | MIR1283-2 |
| FSIP1 | BCL7A |
| FNDC1 | C5orf24 |
| ISX | FAM201A |
| CT45A1 | GACAT3 |
| MIR659 | SNORD51 |
| MIR4505 | LOC113664106 |
| MIR5188 | RNF112 |
| PICART1 | ARID3B |
| MIR646 | H1-1 |
| UC.134 | CCDC26 |
| BAZ1B | SNHG29 |
| FOXN3 | MIR138-2 |
| RHOV | LINC00977 |
| ALDH18A1 | MIR153-1 |
| C1QC | LINC00683 |
| NAA10 | LINC00976 |
| PVR | MIR4732 |
| COPB2 | MIR602 |
| RAD17 | MIR29B2CHG |
| VPS35 | MIR23AHG |
| CAPG | MIR5703 |
| CSRP2 | ETV4 |
| EIF2A | TOB1 |
| IL1RL1 | SNORD3B-1 |
| PDIA4 | LINC01436 |
| IL16 | DBET |
| VIL1 | SNORD3B-2 |
| FYCO1 | MIR664B |
| TOX | MIR384 |
| AMOTL2 | RF00873 |
| MTREX | ELF1 |
| SUB1 | HOXA11 |
| RO60 | TUBG2 |
| S1PR2 | USP2-AS1 |
| CDK12 | LINC01150 |
| PPL | MIR891A |
| CAMSAP2 | LOC124904138 |
| SYNM | LOC124904135 |
| SLC25A24 | LOC124904136 |
| CPS1 | LOC124904137 |
| RDH5 | LOC124904139 |
| DVL2 | LOC124904140 |
| FGD4 | LOC124904141 |
| SMYD2 | LOC124904142 |
| KIF2A | LOC124904143 |
| MGP | LOC124904144 |
| SFPQ | LOC124904146 |
| CTNNA2 | LOC124907963 |
| ANKRD1 | LOC124907964 |
| NUP93 | LOC124907965 |
| WWP1 | GAS7 |
| RNF40 | DKK4 |
| EIF3G | MAP7D3 |
| SRSF5 | MISP |
| TPM4 | COLCA1 |
| BAG6 | RNU5A-1 |
| HOXB2 | ARLNC1 |
| AQP8 | PURPL |
| DNAJC10 | MIR3196 |
| RCHY1 | AFAP1 |
| TRIM68 | ELF5 |
| ATP5MK | GADD45B |
| MRPS31 | SFI1 |
| MARCHF1 | ZNF232 |
| LINC00852 | LINC01565 |
| DUBR | SNHG8 |
| MIR124-2HG | PANTR1 |
| GAS6-DT | ATP8B1-AS1 |
| PCED1B-AS1 | MIR5196 |
| MIR2467 | ELFN1-AS1 |
| CASC20 | MIR3148 |
| MIR1205 | MIR3942 |
| MIR3918 | EEF1B2P5 |
| MIR3658 | EF177379 |
| DINOL | HOXB7 |
| MIR1321 | MRGPRD |
| MIR1233-1 | FOXN2 |
| MIR6732 | FAM30A |
| DLG1 | MCTS2 |
| MIR488 | HMGB3 |
| TIAM2 | DKK2 |
| CRISPLD2 | ZBTB17 |
| LY6K | REG4 |
| FRMD5 | ARHGEF19 |
| SCGB2A2 | MRGPRX1 |
| SPINK7 | IRF2BP1 |
| TMED3 | TRIM59 |
| C14orf28 | TBC1D9B |
| MAPKAPK5-AS1 | H3-5 |
| MIR614 | TMEM105 |
| SGO1-AS1 | PICSAR |
| TM4SF1-AS1 | MIR517C |
| MIR4478 | MIR628 |
| ZEBTR | MALINC1 |
| CCL1 | MIR3911 |
| SPINT2 | PWRN2 |
| ACO2 | LINC00278 |
| LARS1 | MIR4524A |
| MAP2K7 | MIR4270 |
| PRKAR2A | ENSG00000269966 |
| UQCRC2 | ENSG00000234050 |
| SLC27A4 | LOC108510657 |
| KCNJ12 | RGS12 |
| TRIM32 | TOB2 |
| DDX11 | KLRC2 |
| TAF15 | L1TD1 |
| TARS1 | CACUL1 |
| ARHGAP9 | LINC00299 |
| CCR8 | RUNX1-IT1 |
| DDX39B | STXBP5-AS1 |
| LMO1 | OVAAL |
| NCAPD2 | SNORD36C |
| NFYC | TSPOAP1-AS1 |
| ADAP1 | ITIH4-AS1 |
| SAP30 | MIR3175 |
| SPON2 | MIR4672 |
| TSPAN31 | ERLNC1 |
| CENPU | LINC01215 |
| EDC4 | RNA5S10 |
| EIF3M | RNA5S11 |
| NUMBL | RNA5S12 |
| PDIA6 | RNA5S13 |
| RAB9A | RNA5S14 |
| RFX1 | RNA5S15 |
| SAP18 | RNA5S16 |
| WDR4 | RNA5S17 |
| DSTN | RNA5S1 |
| RAB3IP | RNA5S2 |
| RHOJ | RNA5S3 |
| HNRNPUL1 | RNA5S4 |
| MAL | RNA5S5 |
| ATP5IF1 | RNA5S6 |
| GOLGB1 | RNA5S7 |
| RBM27 | RNA5S8 |
| TRIM56 | RUNX2-AS1 |
| FBXO22 | MIR5571 |
| AJAP1 | MIR6079 |
| AMBN | LOC124900560 |
| MIR629 |  |
| MIR559 |  |
| MIR4784 |  |
| MYBBP1A |  |
| KCNMA1 |  |
| DAG1 |  |
| LINC00996 |  |
| LINC01655 |  |
| LINC02257 |  |
| MIR4728 |  |
| CM034961-284 |  |
| SYT1 |  |
| GABRA3 |  |
| PRKCSH |  |
| TNKS |  |
| LILRB1 |  |
| LRP4 |  |
| PFAS |  |
| VARS1 |  |
| ADAMTS18 |  |
| FGFRL1 |  |
| LDHC |  |
| LEFTY1 |  |
| MAP3K4 |  |
| WDR26 |  |
| CCR9 |  |
| IARS2 |  |
| IQSEC1 |  |
| MEIS1 |  |
| NR2E1 |  |
| SEMA5A |  |
| TRIP4 |  |
| TXNDC5 |  |
| ABCF1 |  |
| DHRS2 |  |
| LHX1 |  |
| LY6E |  |
| BAZ2A |  |
| CORO1C |  |
| FCRL5 |  |
| GALNT6 |  |
| LTB |  |
| PARP10 |  |
| PDCD5 |  |
| RAB1B |  |
| SNX2 |  |
| SSH1 |  |
| TNRC6B |  |
| ESYT1 |  |
| MYO1D |  |
| PYGO2 |  |
| TBL1X |  |
| TOMM70 |  |
| TRIB2 |  |
| DOCK5 |  |
| EPB41L4B |  |
| LHX2 |  |
| PLP2 |  |
| PODXL2 |  |
| TMBIM6 |  |
| UBP1 |  |
| ANKS1A |  |
| GBP2 |  |
| GPR34 |  |
| OTUD7B |  |
| POLDIP2 |  |
| AHDC1 |  |
| RABL6 |  |
| SHCBP1 |  |
| TUT1 |  |
| BAALC |  |
| GINS4 |  |
| HASPIN |  |
| MED26 |  |
| MTFR2 |  |
| PSD4 |  |
| CST2 |  |
| CHRFAM7A |  |
| USP51 |  |
| DCTPP1 |  |
| HADHB |  |
| GRIN2B |  |
| SUMO1 |  |
| MIR208B |  |
| KRT4 |  |
| IRAK1 |  |
| ADRB1 |  |
| ARHGAP26 |  |
| GGPS1 |  |
| PDGFC |  |
| CHSY1 |  |
| CTSV |  |
| EFNA4 |  |
| RASGRP3 |  |
| DLGAP1 |  |
| ELF1 |  |
| GRAP |  |
| KLRC1 |  |
| KSR1 |  |
| NAXE |  |
| NXN |  |
| PELI1 |  |
| PLEKHG5 |  |
| RNPEP |  |
| SLC6A14 |  |
| SORBS2 |  |
| THOP1 |  |
| UBE2R2 |  |
| USP25 |  |
| AGO1 |  |
| COL13A1 |  |
| FGFBP1 |  |
| ICMT |  |
| RAB8B |  |
| B3GNT3 |  |
| BTG1 |  |
| CAB39 |  |
| CD248 |  |
| CNN2 |  |
| DCDC2 |  |
| DLX6 |  |
| EPB41L2 |  |
| IP6K1 |  |
| LYPLA1 |  |
| MCF2 |  |
| NMB |  |
| NTN4 |  |
| OTUD7A |  |
| PATZ1 |  |
| POU3F3 |  |
| RAB35 |  |
| SLC38A3 |  |
| STARD13 |  |
| STK17A |  |
| SUGCT |  |
| TASP1 |  |
| ANXA9 |  |
| APBB3 |  |
| ATG10 |  |
| EBI3 |  |
| EDARADD |  |
| GRSF1 |  |
| HTR1E |  |
| KLF13 |  |
| KLF3 |  |
| MRI1 |  |
| NSRP1 |  |
| ORAI2 |  |
| PARP12 |  |
| PBLD |  |
| PNO1 |  |
| RASSF4 |  |
| RNF128 |  |
| SRSF11 |  |
| TGIF2 |  |
| USP33 |  |
| ARID4B |  |
| DHX32 |  |
| HAO2 |  |
| HOXA6 |  |
| PARD6B |  |
| RNF138 |  |
| RUSC1 |  |
| SAP130 |  |
| TMSB10 |  |
| TSPAN2 |  |
| TXNDC12 |  |
| BDH2 |  |
| BLOC1S1 |  |
| FOXL1 |  |
| GDE1 |  |
| ARL4C |  |
| CMTM3 |  |
| DCAF11 |  |
| GPS2 |  |
| PCDH20 |  |
| RNF38 |  |
| SH3BGRL3 |  |
| STYX |  |
| TSKU |  |
| ZBTB11 |  |
| FBXL16 |  |
| MRGBP |  |
| PCP4 |  |
| REG3G |  |
| SPSB3 |  |
| STRIP2 |  |
| WDFY2 |  |
| ZNF334 |  |
| GRINA |  |
| PRRC2B |  |
| TMEM158 |  |
| ZNF267 |  |
| ZNF471 |  |
| JPT2 |  |
| KCTD11 |  |
| LRATD1 |  |
| PLAAT4 |  |
| ST7L |  |
| TAFA5 |  |
| N4BP3 |  |
| TM4SF18 |  |
| GPR75 |  |
| NCBP3 |  |
| SPINK6 |  |
| OR1G1 |  |
| CEBPA-DT |  |
| HOXD-AS2 |  |
| MIR578 |  |
| LINC00944 |  |
| MIR325 |  |
| MIR602 |  |
| TRG-CCC2-2 |  |
| TRG-CCC2-1 |  |
| SP3 |  |
| LRPAP1 |  |
| PRKAR1B |  |
| ACSS2 |  |
| RORB |  |
| MYO1E |  |
| PTPRM |  |
| ARHGAP4 |  |
| ADAM23 |  |
| ATP12A |  |
| CDH12 |  |
| GLS2 |  |
| LIPG |  |
| MCF2L |  |
| TNFSF14 |  |
| TRPC5 |  |
| MYSM1 |  |
| NAPSA |  |
| CKAP2L |  |
| GPX8 |  |
| IL34 |  |
| MAFK |  |
| PKNOX1 |  |
| AP1G2 |  |
| EGFL6 |  |
| IRF2BP2 |  |
| PDZD2 |  |
| ZBTB38 |  |
| HIVEP1 |  |
| IGSF9 |  |
| WWC2 |  |
| C1GALT1C1 |  |
| CEMIP2 |  |
| KRT80 |  |
| LARP4 |  |
| OMA1 |  |
| TMEM63C |  |
| ZKSCAN4 |  |
| CXXC4 |  |
| DCAF5 |  |
| HNRNPUL2 |  |
| MYCT1 |  |
| PRMT9 |  |
| TGFBRAP1 |  |
| NRARP |  |
| POTEE |  |
| TMEM139 |  |
| CT83 |  |
| MINAR1 |  |
| EIF3CL |  |
| TMEM229A |  |
| SPANXA2 |  |
| TDRG1 |  |
| MIR181B2 |  |
| SFTA1P |  |
| HOXC13-AS |  |
| MIR1915 |  |
| LINC00443 |  |
| MIR1301 |  |
| SNORD51 |  |
| ELN-AS1 |  |
| GRASLND |  |
| LINC00551 |  |
| MIR597 |  |
| SEMA3B-AS1 |  |
| MIR365B |  |
| OTUD6B-AS1 |  |
| LINC02273 |  |
| LOC105372273 |  |
| MIR581 |  |
| MIR642B |  |
| LINC01613 |  |
| MIR4435-2 |  |
| MIR5194 |  |
| MIR6838 |  |
| MIR5590 |  |
| RPS6KA1 |  |
| RPS20 |  |
| SNHG33 |  |
| USP21 |  |
| PAICS |  |
| TWNK |  |
| EDN2 |  |
| RPS7 |  |
| PTS |  |
| WDR6 |  |
| DSC3 |  |
| GABARAP |  |
| ASIC3 |  |
| EXOC4 |  |
| POU4F1 |  |
| IRX5 |  |
| FEZF1 |  |
| HOXB3 |  |
| ISLR |  |
| KLK12 |  |
| NPNT |  |
| SHC2 |  |
| GPR85 |  |
| TOX3 |  |
| ZRANB1 |  |
| CYP4Z1 |  |
| ZNF407 |  |
| FOXO6 |  |
| PRAG1 |  |
| KRTAP2-3 |  |
| ANXA2P2 |  |
| TPTE2P1 |  |
| LBX2-AS1 |  |
| LINC00265 |  |
| MIR541 |  |
| BOK-AS1 |  |
| LINC00491 |  |
| MIR518F |  |
| PCBP1-AS1 |  |
| LINC00885 |  |
| LINC01426 |  |
| GAS5-AS1 |  |
| LINC00543 |  |
| LINC00641 |  |
| LINC02580 |  |
| MELTF-AS1 |  |
| MIR3199-2 |  |
| MIR4496 |  |
| MIR4677 |  |
| MIR4766 |  |
| IKBKB-DT |  |
| LINC00165 |  |
| LINC01762 |  |
| MIR3691 |  |
| MIR4698 |  |
| MIR4742 |  |
| RAB6C-AS1 |  |
| SGMS1-AS1 |  |
| WFDC21P |  |
| LINC01269 |  |
| LINC01793 |  |
| LINC02200 |  |
| LRRC52-AS1 |  |
| MIR451B |  |
| MIR513B |  |
| MIR580 |  |
| CASC21 |  |
| KAZN-AS1 |  |
| LINC01347 |  |
| LNCSRLR |  |
| MIR1297 |  |
| MIR3182 |  |
| MIR3184 |  |
| MIR4518 |  |
| MIR4709 |  |
| MIR761 |  |
| SNORD114-28 |  |
| SNORD116-14 |  |
| LDC1P |  |
| LOC101929470 |  |
| MIR4270 |  |
| MIR924 |  |
| MIR1233-2 |  |
| MIR3650 |  |
| HSPB1P1 |  |
| LINC01061 |  |
| MIR103B1 |  |
| MIR5694 |  |
| ENSG00000224818 |  |
| ENSG00000224950 |  |
| ENSG00000236452 |  |
| MIR103B2 |  |
| MIR6839 |  |
| ENSG00000229642 |  |
| ENSG00000285673 |  |
| LOC108353817 |  |
| LOC111365171 |  |
| MERTK |  |
| CSPG4 |  |
| AFDN-DT |  |
| ITGB2-AS1 |  |
| LINC01783 |  |
| MIR514B |  |
| MYCNUT |  |
| MIR5193 |  |
| SMASR |  |
| LINC01939 |  |
| ATXN3 |  |
| MIR4485 |  |
| CALB1 |  |
| DAOA-AS1 |  |
| MPRIP |  |
| DDX6 |  |
| CLCN5 |  |
| TRPM2 |  |
| NACA |  |
| RPS25 |  |
| ARPP19 |  |
| NEUROD1 |  |
| ATAD3A |  |
| RPS9 |  |
| HACD3 |  |
| F13A1 |  |
| MYLK |  |
| FPR1 |  |
| ABL2 |  |
| BDKRB2 |  |
| VAPB |  |
| CD2AP |  |
| BCAS2 |  |
| ZRANB2 |  |
| EFS |  |
| PADI2 |  |
| UBE2M |  |
| PDHB |  |
| ABCD3 |  |
| SRPX2 |  |
| FAM120A |  |
| SNRPD1 |  |
| LSM14A |  |
| PELO |  |
| COL17A1 |  |
| SUN2 |  |
| ALDH7A1 |  |
| HMGCL |  |
| CDCA3 |  |
| LPAR2 |  |
| ELOC |  |
| QPCT |  |
| ARF4 |  |
| RPL24 |  |
| CACYBP |  |
| SNRPB2 |  |
| TRPC3 |  |
| NDUFS3 |  |
| RANGAP1 |  |
| SORBS1 |  |
| SRP72 |  |
| GIGYF2 |  |
| CLK2 |  |
| RPL30 |  |
| H2AC4 |  |
| KIR2DS4 |  |
| MIR3940 |  |
| IMPDH2 |  |
| VASH1 |  |
| TRAM2 |  |
| GTF2IRD1 |  |
| MIR520A |  |
| ATP1A3 |  |
| EGR2 |  |
| CELF2 |  |
| TMPRSS6 |  |
| EIF4A2 |  |
| ATG16L1 |  |
| EXOSC9 |  |
| RGMA |  |
| SOD3 |  |
| ATXN2L |  |
| CLEC1B |  |
| ACTBL2 |  |
| H2BC3 |  |
| RAP1B |  |
| TECR |  |
| DNAJA3 |  |
| MED16 |  |
| PPT2-EGFL8 |  |
| OSBPL1A |  |
| LIPE-AS1 |  |
| AMELX |  |
| FGF14 |  |
| GNG2 |  |
| PIAS4 |  |
| WDR1 |  |
| SQOR |  |
| CCAR1 |  |
| MRPS23 |  |
| PPP1R7 |  |
| RAP1GAP |  |
| DCAF7 |  |
| RPL38 |  |
| SAV1 |  |
| TRIM26 |  |
| FAM83H |  |
| IFITM2 |  |
| HECTD2 |  |
| RAB34 |  |
| NREP |  |
| PAQR4 |  |
| MIR548B |  |
| ACP5 |  |
| CBY1 |  |
| DIO2 |  |
| HSD17B12 |  |
| SPIB |  |
| NUDCD1 |  |
| MAP7D1 |  |
| SPINDOC |  |
| MIR4739 |  |
| PXDNL |  |
| SNHG10 |  |
| XRCC2 |  |
| CACNA1B |  |
| NTF4 |  |
| NSDHL |  |
| SLC22A3 |  |
| AIP |  |
| SRPK2 |  |
| ARHGAP31 |  |
| ATP5PO |  |
| MICAL1 |  |
| PDSS2 |  |
| PORCN |  |
| AK4 |  |
| VAMP7 |  |
| MRC2 |  |
| NEK4 |  |
| DOCK10 |  |
| GEMIN2 |  |
| PLXDC1 |  |
| ARIH1 |  |
| CPEB4 |  |
| DPP8 |  |
| LPAR5 |  |
| RAB11FIP1 |  |
| AP2A2 |  |
| NEIL3 |  |
| PIWIL2 |  |
| RAI14 |  |
| SEC62 |  |
| NHERF2 |  |
| DIXDC1 |  |
| SARNP |  |
| ZFP91 |  |
| CAMSAP3 |  |
| GADD45GIP1 |  |
| LMTK3 |  |
| NEK5 |  |
| UBTD1 |  |
| SEPT5-GP1BB |  |
| PPP1R13B-DT |  |
| FABP7 |  |
| CRABP1 |  |
| IL15RA |  |
| RNU105B |  |
| TGM4 |  |
| DHCR7 |  |
| CHD1 |  |
| AGRN |  |
| ANTXR1 |  |
| PRDM16 |  |
| LPP |  |
| PIAS2 |  |
| RALBP1 |  |
| SLC39A8 |  |
| NDUFB9 |  |
| S100A10 |  |
| SLC2A5 |  |
| TNNI2 |  |
| ARHGEF12 |  |
| RANBP9 |  |
| CISD2 |  |
| PARG |  |
| RAB2A |  |
| MFNG |  |
| OPN4 |  |
| ADGRF5 |  |
| MPZL2 |  |
| TBC1D7 |  |
| ZNF143 |  |
| ANGPTL1 |  |
| MICA |  |
| MYCBP |  |
| NDRG4 |  |
| SGMS2 |  |
| TIGAR |  |
| AGBL2 |  |
| FRMD6 |  |
| MOB1B |  |
| NEU4 |  |
| GRHL1 |  |
| HINT2 |  |
| NOP14 |  |
| RBMS3 |  |
| ZBTB2 |  |
| ZNF326 |  |
| COL28A1 |  |
| ESF1 |  |
| PKNOX2 |  |
| SETD6 |  |
| SPRR1A |  |
| BRMS1L |  |
| OIP5 |  |
| DNAJB8 |  |
| PRSS50 |  |
| FBXO16 |  |
| LDLRAD2 |  |
| AK6 |  |
| TCL6 |  |
| TMEM51-AS1 |  |
| SKAP1-AS2 |  |
| MIR4472-2 |  |
| MIR4472-1 |  |
| HOXD11 |  |
| ITGAX |  |
| LRSAM1 |  |
| CXCL3 |  |
| FER |  |
| PICALM |  |
| ABCC5 |  |
| ADGRE5 |  |
| CRBN |  |
| PAK6 |  |
| JAM2 |  |
| PROK2 |  |
| RREB1 |  |
| CFHR3 |  |
| CHST15 |  |
| UFM1 |  |
| HAS3 |  |
| RNF125 |  |
| CERK |  |
| EBF3 |  |
| IGSF8 |  |
| SOX13 |  |
| THEM4 |  |
| BCO1 |  |
| ITFG1 |  |
| PPM1H |  |
| REEP3 |  |
| SCARA3 |  |
| SHROOM2 |  |
| CORO7 |  |
| MPP7 |  |
| SHKBP1 |  |
| UBE2W |  |
| AFAP1L1 |  |
| ARHGAP25 |  |
| KPNA5 |  |
| LIMCH1 |  |
| NT5DC2 |  |
| PHF20L1 |  |
| POLR1H |  |
| RTN4RL1 |  |
| SGTB |  |
| GPR19 |  |
| NAA40 |  |
| NDUFA4L2 |  |
| RER1 |  |
| VPS37B |  |
| RNFT2 |  |
| TMC7 |  |
| ACTL8 |  |
| CLEC17A |  |
| KPNA7 |  |
| ZBTB3 |  |
| YY2 |  |
| HYMAI |  |
| ZNF252P-AS1 |  |
| HAR1B |  |
| MIR9-3HG |  |
| RAB11B-AS1 |  |
| BTG2-DT |  |
| MIR3188 |  |
| MIR450A1 |  |
| MIR4319 |  |
| MIR6873 |  |
| PSMA3-AS1 |  |
| GFRA2 |  |
| DNTT |  |
| EMX2 |  |
| DGKG |  |
| MLLT10 |  |
| ARHGAP29 |  |
| CXXC5 |  |
| HOXA7 |  |
| HOXD8 |  |
| NOVA2 |  |
| ARHGAP45 |  |
| GREB1 |  |
| NOSTRIN |  |
| ZFP42 |  |
| SLC30A3 |  |
| DBX2 |  |
| CPTP |  |
| LDLRAD3 |  |
| USP27X |  |
| CCDC144NL |  |
| OR10G2 |  |
| C16orf96 |  |
| PLAC4 |  |
| LINC02915 |  |
| LINC00174 |  |
| LINC00323 |  |
| MIR376C |  |
| NRON |  |
| MALINC1 |  |
| LINC00964 |  |
| LINC01108 |  |
| MIR1237 |  |
| MIR517B |  |
| DLEU1-AS1 |  |
| LINC00273 |  |
| MIR550A3 |  |
| MIR643 |  |
| CD44-DT |  |
| LDLRAD4-AS1 |  |
| MIR3190 |  |
| MIR4279 |  |
| MIR4746 |  |
| MIR513A2 |  |
| RPAP3-DT |  |
| LNCBRM |  |
| MIR3144 |  |
| TGFB2-OT1 |  |
| MIR378G |  |
| ENSG00000250362 |  |
| MIR3201 |  |
| MIR4282 |  |
| COL1A2-AS1 |  |
| ENSG00000272021 |  |
| ENSG00000228741 |  |
| LOC102724720 |  |
| ST2 |  |
| LOC107832851 |  |
| LOC109113863 |  |
| LUM |  |
| ADAM33 |  |
| ABCA2 |  |
| ANLN |  |
| LDHB |  |
| LINC00649 |  |
| MIR1229 |  |
| COL26A1 |  |
| KLF7 |  |
| LIMD1 |  |
| FXR1 |  |
| FGF4 |  |
| MYL6 |  |
| CFTR-AS1 |  |
| FUCA1 |  |
| DLX3 |  |
| MIR320C1 |  |
| NR1I3 |  |
| PCDH7 |  |
| ADAM19 |  |
| COL8A2 |  |
| HOXC4 |  |
| HUNK |  |
| SH3RF3 |  |
| ST7-AS1 |  |
| LINC00525 |  |
| HOXC-AS1 |  |
| ADAMTS9-AS1 |  |
| SNORA21 |  |
| B4GALT1-AS1 |  |
| MIR1283-1 |  |
| MIR1303 |  |
| MIR298 |  |
| MIR3120 |  |
| SNORA80E |  |
| LINC00332 |  |
| LINC01287 |  |
| MIR621 |  |
| RPL26L1-AS1 |  |
| STEAP1B-AS1 |  |
| LINC01303 |  |
| MAFTRR |  |
| MIR1265 |  |
| MIR3614 |  |
| MIR518D |  |
| MSTO2P |  |
| HSPC324 |  |
| MIR4262 |  |
| ENSG00000232949 |  |
| MIR6783 |  |
| MIR6754 |  |
| ANCR |  |
| lnc-GNAQ-6 |  |
| BEST1 |  |
| PURA |  |
| PLTP |  |
| SNRNP70 |  |
| PRDX6 |  |
| FTO |  |
| LOC110806263 |  |
| DNAJC3-DT |  |
| FAS-AS1 |  |
| ASS1 |  |
| GNB4 |  |
| NOM1 |  |
| EMID1 |  |
| CDH9 |  |
| LAMA2 |  |
| GYPA |  |
| PPIL2 |  |
| RECQL5 |  |
| SSBP2 |  |
| DDA1 |  |
| MAGEA12 |  |
| RPL11 |  |
| MIR337 |  |
| LLGL1 |  |
| PDLIM5 |  |
| LARP1 |  |
| SCN8A |  |
| RPS3A |  |
| RPS18 |  |
| MIR320C2 |  |
| DNAJB6 |  |
| RPL5 |  |
| NEU1 |  |
| DUSP19 |  |
| OLIG1 |  |
| PRPS1 |  |
| CPT1A |  |
| POU1F1 |  |
| RPL6 |  |
| TBX4 |  |
| EMILIN3 |  |
| RGMB-AS1 |  |
| RPS5 |  |
| MIR3064 |  |
| SLC12A3 |  |
| IRF4 |  |
| KDM5C |  |
| BCL2A1 |  |
| RBM10 |  |
| NSD3 |  |
| G6PC3 |  |
| YBX3 |  |
| IL25 |  |
| MIR365A |  |
| LIFR-AS1 |  |
| LINC01224 |  |
| ODC1-DT |  |
| MIR3147 |  |
| RF00873 |  |
| SLC4A1 |  |
| OAT |  |
| SAMHD1 |  |
| CCT2 |  |
| CCT7 |  |
| RPS11 |  |
| U2AF2 |  |
| CSN2 |  |
| MSTN |  |
| ENPP2 |  |
| FSCN2 |  |
| SLC25A11 |  |
| EEF1G |  |
| HNRNPA3 |  |
| ARL2 |  |
| LNX1 |  |
| HSPA14 |  |
| ARL2BP |  |
| COPS2 |  |
| ZFX |  |
| OTUB2 |  |
| PHF14 |  |
| MIR320E |  |
| TMA7 |  |
| MCOLN1 |  |
| RPS2 |  |
| RPS23 |  |
| RCN2 |  |
| ATAD3B |  |
| MRPS27 |  |
| MRPS34 |  |
| SNRPA1 |  |
| RTRAF |  |
| DLX5 |  |
| DKK2 |  |
| PLA1A |  |
| HOXD10 |  |
| KLHL20 |  |
| PAN2 |  |
| CENPI |  |
| CHMP4C |  |
| BCORL1 |  |
| TRIM47 |  |
| RBMY1A1 |  |
| ZNF677 |  |
| MIRLET7F2 |  |
| MIR4324 |  |
| LRP2 |  |
| HSD17B10 |  |
| RPS10 |  |
| RPS24 |  |
| RPL3 |  |
| NCK2 |  |
| RPL19 |  |
| RPL8 |  |
| SSBP1 |  |
| BAG2 |  |
| MUS81 |  |
| RPL29 |  |
| MXD1 |  |
| CALML5 |  |
| GRIA2 |  |
| ADORA1 |  |
| NDUFS8 |  |
| DHPS |  |
| RPL10 |  |
| RPS14 |  |
| FAF1 |  |
| PRPF6 |  |
| NUP205 |  |
| RPLP1 |  |
| GPHA2 |  |
| RNU5A-1 |  |
| MMP17 |  |
| FREM1 |  |
| TBX18 |  |
| FRAS1 |  |
| USP32 |  |
| SLC25A33 |  |
| TMEM52B |  |
| LINC00523 |  |
| PTCSC3 |  |
| MIR320B1 |  |
| MATN1-AS1 |  |
| MIR320B2 |  |
| MIR636 |  |
| MIR941-2 |  |
| MIR941-4 |  |
| MIR941-3 |  |
| MIR941-5 |  |
| CALCR |  |
| ST3GAL5 |  |
| MAT2A |  |
| CKM |  |
| DGKA |  |
| COL5A2 |  |
| RPL27 |  |
| SBF1 |  |
| RPL13A |  |
| RBM17 |  |
| RBM5 |  |
| RTCB |  |
| COX7A1 |  |
| SPTAN1 |  |
| CYSLTR2 |  |
| USP8 |  |
| ACAT2 |  |
| ATF1 |  |
| CUBN |  |
| ETFA |  |
| SLC25A1 |  |
| ARPC1B |  |
| PRPF31 |  |
| ERLIN2 |  |
| DHX30 |  |
| RPL31 |  |
| UBE2K |  |
| RPL34 |  |
| ATP5F1C |  |
| GTPBP4 |  |
| HERC5 |  |
| MPP1 |  |
| RNF20 |  |
| CDCA2 |  |
| CLNS1A |  |
| DDX39A |  |
| EIF3L |  |
| SNRPD2 |  |
| SYMPK |  |
| EXOSC4 |  |
| RPP30 |  |
| GCN1 |  |
| PURB |  |
| ECPAS |  |
| IGHG1 |  |
| ICE2 |  |
| HIPK3 |  |
| STRA6 |  |
| HOXA2 |  |
| FUCA2 |  |
| FREM2 |  |
| KRT75 |  |
| OSR2 |  |
| MMRN2 |  |
| USP43 |  |
| PTTG3P |  |
| NAP1L6P |  |
| PWRN1 |  |
| MIR137HG |  |
| MIR1262 |  |
| MIR1207 |  |
| MRPL23-AS1 |  |
| HMGA2-AS1 |  |
| LINC00662 |  |
| LINC01128 |  |
| PRRT3-AS1 |  |
| ARHGEF26-AS1 |  |
| LINC00920 |  |
| MIR606 |  |
| MIR607 |  |
| MIR937 |  |
| PTCSC1 |  |
| TNK2-AS1 |  |
| ZSCAN16-AS1 |  |
| LINC01050 |  |
| LINC02487 |  |
| MIR4645 |  |
| MIR623 |  |
| MIR941-1 |  |
| NCK1-DT |  |
| PAXIP1-AS2 |  |
| PWRN3 |  |
| LINC00244 |  |
| LINC01541 |  |
| MIR3167 |  |
| MIR329-2 |  |
| QKILA |  |
| MIR4689 |  |
| THY1-AS1 |  |
| MIR4704 |  |
| MIR4799 |  |
| MIR5571 |  |
| MIR6088 |  |
| RSF1-IT2 |  |
| ARNILA |  |
| MIR4255 |  |
| ENSG00000286110 |  |
| LINC03055 |  |
| PDS5B-DT |  |
| TOMM22-DT |  |
| EGLN1 |  |
| IFNAR1 |  |
| ALDH3A1 |  |
| HMGCS2 |  |
| SCARB2 |  |
| STT3A |  |
| BCKDK |  |
| ADORA3 |  |
| PAPSS2 |  |
| TOP3A |  |
| ATXN10 |  |
| CYSLTR1 |  |
| DDX20 |  |
| EEF1B2 |  |
| FARSA |  |
| PAX1 |  |
| PPBP |  |
| AP3B2 |  |
| APC2 |  |
| EIF1AX |  |
| PIP4K2B |  |
| PRDX4 |  |
| SIK3 |  |
| CXCL6 |  |
| LIN7A |  |
| LLGL2 |  |
| CIR1 |  |
| DPPA4 |  |
| HOXB4 |  |
| RPL18A |  |
| SNX17 |  |
| CMAS |  |
| CPSF7 |  |
| DDX19A |  |
| TSPAN32 |  |
| FAM98A |  |
| ZCCHC10 |  |
| LINC00968 |  |
| MIR1299 |  |
| GRIP1 |  |
| HCG22 |  |
| TAB2 |  |
| EPS15 |  |
| MIB1 |  |
| TLK2 |  |
| IFNGR2 |  |
| TREM2 |  |
| CPA6 |  |
| HAT1 |  |
| PLD3 |  |
| RARS2 |  |
| TCN2 |  |
| AUTS2 |  |
| CHRDL1 |  |
| CPA4 |  |
| CSRP1 |  |
| GPR39 |  |
| RAB10 |  |
| RECQL |  |
| RHOT1 |  |
| TOP1MT |  |
| AHSA1 |  |
| DAP3 |  |
| MFAP4 |  |
| SSR1 |  |
| TFF2 |  |
| ALG13 |  |
| ARID3B |  |
| CCDC88C |  |
| CEP170 |  |
| DSC1 |  |
| PTCD3 |  |
| RARRES2 |  |
| SHC3 |  |
| USP53 |  |
| AGMAT |  |
| GALNT4 |  |
| GAR1 |  |
| IPO11 |  |
| NOVA1 |  |
| DTX3L |  |
| NRBP1 |  |
| RAB17 |  |
| TMTC3 |  |
| VBP1 |  |
| ZIC4 |  |
| BRIX1 |  |
| CLUH |  |
| OTUD4 |  |
| THOC3 |  |
| USP36 |  |
| AUP1 |  |
| CENPK |  |
| FBXO2 |  |
| PHRF1 |  |
| RILP |  |
| JPH4 |  |
| TRMT13 |  |
| SAPCD2 |  |
| H3-5 |  |
| MIRLET7BHG |  |
| FAM66D |  |
| FAM66A |  |
| NPHP3-ACAD11 |  |
| FAM86B2-DT |  |
| TRK-TTT3-5 |  |
| ENSG00000255495 |  |
| TRK-TTT3-4 |  |
| TRG-GCC3-1 |  |
| TRK-TTT3-1 |  |
| TRK-TTT3-2 |  |
| TRK-TTT3-3 |  |
| B4GALNT1 |  |
| FDFT1 |  |
| MADD |  |
| MAPK6 |  |
| CRLF1 |  |
| EFNA3 |  |
| HAPLN1 |  |
| IVNS1ABP |  |
| NTNG1 |  |
| PIP5K1B |  |
| USP20 |  |
| BPNT2 |  |
| CELSR1 |  |
| CTSE |  |
| DOCK4 |  |
| ECI2 |  |
| ELMO2 |  |
| LMOD1 |  |
| MS4A2 |  |
| SPEN |  |
| UBE2J1 |  |
| VPS13B |  |
| CMKLR1 |  |
| LDHD |  |
| NELL2 |  |
| PRELP |  |
| RBP2 |  |
| SLC4A11 |  |
| SNX10 |  |
| SOCS6 |  |
| STK25 |  |
| TAB3 |  |
| APBA3 |  |
| CNOT4 |  |
| ECH1 |  |
| EEF1E1 |  |
| HS6ST2 |  |
| LAMTOR3 |  |
| NUDT2 |  |
| RSPO3 |  |
| SAR1A |  |
| USP6NL |  |
| ARL1 |  |
| BAG4 |  |
| CDCA5 |  |
| DCUN1D1 |  |
| DNAJB9 |  |
| NELFCD |  |
| UBA6 |  |
| USP28 |  |
| BCL6B |  |
| CMKLR2 |  |
| COPS7B |  |
| DIP2B |  |
| HJURP |  |
| LILRB4 |  |
| RTCA |  |
| TMEM135 |  |
| XCR1 |  |
| C1QTNF6 |  |
| COPG2 |  |
| JOSD1 |  |
| NDST3 |  |
| SELENOS |  |
| ASB3 |  |
| COPS4 |  |
| EMP1 |  |
| GMFG |  |
| JOSD2 |  |
| METTL5 |  |
| SAMD9 |  |
| ZNF667 |  |
| AKIRIN2 |  |
| BPIFB1 |  |
| CBLN4 |  |
| COMMD3 |  |
| KCTD5 |  |
| PRPSAP2 |  |
| QPCTL |  |
| RASSF8 |  |
| SDAD1 |  |
| ZNF598 |  |
| DUSP11 |  |
| SCRT1 |  |
| TMEM106A |  |
| YOD1 |  |
| ZC3H13 |  |
| OBI1 |  |
| XCL2 |  |
| MRPL52 |  |
| TMEM263 |  |
| DCAF4L2 |  |
| MIR592 |  |
| SNORA23 |  |
| MIR640 |  |
| SNORD49A |  |
| HERC2 |  |
| PTPRN |  |
| HCLS1 |  |
| HYAL2 |  |
| AFDN |  |
| CDC42BPA |  |
| PLCD3 |  |
| RPS6KA6 |  |
| CBFA2T3 |  |
| CD8B |  |
| PPM1F |  |
| HHEX |  |
| GDF3 |  |
| HOXA3 |  |
| PIP4K2C |  |
| G6PC2 |  |
| GEMIN4 |  |
| LONP2 |  |
| NEURL1 |  |
| RIPOR2 |  |
| TBX22 |  |
| TRMT10C |  |
| JAML |  |
| MCOLN2 |  |
| PPM1E |  |
| TTYH2 |  |
| ERCC6L |  |
| ITPRIP |  |
| LINGO2 |  |
| MYDGF |  |
| ZNF507 |  |
| MACROD2 |  |
| PLEKHM3 |  |
| RNF145 |  |
| USP54 |  |
| WTIP |  |
| NEURL4 |  |
| TMEM200A |  |
| COPRS |  |
| CARMIL3 |  |
| SHISA3 |  |
| ODR4 |  |
| LINC02875 |  |
| LINC00336 |  |
| LINC02908 |  |
| PRR34 |  |
| LINC00518 |  |
| PART1 |  |
| ZNF561-AS1 |  |
| LINC00320 |  |
| PTPRG-AS1 |  |
| CDH23-AS1 |  |
| LINC02905 |  |
| MIR1276 |  |
| MIR497HG |  |
| OXCT1-AS1 |  |
| SLC7A11-AS1 |  |
| VCAN-AS1 |  |
| ALOX12-AS1 |  |
| COMETT |  |
| EIF3J-DT |  |
| EOLA1-DT |  |
| LINC00488 |  |
| LINC01091 |  |
| NRAD1 |  |
| SNORD38B |  |
| LNCAROD |  |
| MIR320D1 |  |
| VTRNA1-2 |  |
| SNORD81 |  |
| GJA9-MYCBP |  |
| MIR1827 |  |
| MIR3131 |  |
| TRG-GCC2-6 |  |
| ENSG00000267047 |  |
| MIR6504 |  |
| SNAI1P1 |  |
| TRG-TCC1-1 |  |
| TRG-GCC2-1 |  |
| TRG-GCC2-3 |  |
| TRG-GCC2-2 |  |
| TRG-GCC2-4 |  |
| TRG-GCC2-5 |  |
| ITGA8 |  |
| CYP26A1 |  |
| HGFAC |  |
| TNFRSF25 |  |
| IPO8 |  |
| CLTCL1 |  |
| DISP1 |  |
| HOXA4 |  |
| PAPPA2 |  |
| FAT2 |  |
| GDF1 |  |
| SOX21 |  |
| ZFHX4 |  |
| ZNFX1 |  |
| DISP2 |  |
| PCDHB3 |  |
| ZNRF2 |  |
| CRACD |  |
| DRAM1 |  |
| PIK3CD-AS1 |  |
| DHRS4-AS1 |  |
| POLR1HASP |  |
| MIR124-1HG |  |
| DLEU2L |  |
| GNG12-AS1 |  |
| LINC00243 |  |
| LINC00654 |  |
| LINC00689 |  |
| LINC01118 |  |
| MIR516A1 |  |
| SNHG18 |  |
| ZNF503-AS1 |  |
| LINC00239 |  |
| LINC01018 |  |
| MIR518A1 |  |
| FGF14-AS2 |  |
| IGFBP7-AS1 |  |
| LINC00882 |  |
| MIR3661 |  |
| MIR518A2 |  |
| MIR548A3 |  |
| MSC-AS1 |  |
| SWINGN |  |
| TMEM72-AS1 |  |
| DPH6-DT |  |
| FAM238C |  |
| FAM66C |  |
| IDH1-AS1 |  |
| LINC00402 |  |
| LINC00485 |  |
| LINC00607 |  |
| LINC01119 |  |
| LINC01123 |  |
| LINC01152 |  |
| LINC01160 |  |
| LINC01446 |  |
| MIR1913 |  |
| MIR3678 |  |
| MIR4515 |  |
| MIR596 |  |
| NUTM2A-AS1 |  |
| PRR34-AS1 |  |
| RFX3-DT |  |
| ZNF790-AS1 |  |
| ADD3-AS1 |  |
| CKMT2-AS1 |  |
| FAM138C |  |
| GNAO1-DT |  |
| LINC01063 |  |
| LINC01608 |  |
| LINC01714 |  |
| LINC01820 |  |
| LINC02544 |  |
| LNCTAM34A |  |
| MIR1298 |  |
| MIR133A1HG |  |
| MIR4284 |  |
| MIR4691 |  |
| MIR520E |  |
| MIR585 |  |
| MIR670 |  |
| RBPMS-AS1 |  |
| ABCA9-AS1 |  |
| CASC18 |  |
| ELFN1-AS1 |  |
| IER3-AS1 |  |
| LINC02112 |  |
| MIR1202 |  |
| MIR320D2 |  |
| MIR3689F |  |
| YTHDF3-DT |  |
| ANXA2P1 |  |
| BABAM2-AS1 |  |
| FLJ45513 |  |
| FNDC1-AS1 |  |
| GPAT4-AS1 |  |
| LINC01228 |  |
| LINC01836 |  |
| LINC01969 |  |
| LINC02454 |  |
| LINC02533 |  |
| LOC148696 |  |
| MIR1257 |  |
| MIR3150A |  |
| MIR3928 |  |
| MIR4454 |  |
| MIR4668 |  |
| MIR4706 |  |
| MIR570HG |  |
| NALF1-IT1 |  |
| SNORD76 |  |
| C4A-AS1 |  |
| LINC01956 |  |
| MIR3149 |  |
| MIR3162 |  |
| MIR378F |  |
| MIR4313 |  |
| MIR4647 |  |
| MIR4652 |  |
| MIR4656 |  |
| MIR4688 |  |
| MIR4695 |  |
| MIR4713 |  |
| MIR4776-1 |  |
| MIR4776-2 |  |
| MIR5088 |  |
| MIR6720 |  |
| C4B-AS1 |  |
| ENSG00000206028 |  |
| MIR3116-1 |  |
| MIR3116-2 |  |
| MIR6823 |  |
| EBLN3P |  |
| ENSG00000228274 |  |
| ENSG00000249406 |  |
| ERVK-10 |  |
| MIR6071 |  |
| MIR6134 |  |
| ENSG00000214797 |  |
| ENSG00000247134 |  |
| ENSG00000267934 |  |
| MIR4433B |  |
| MIR548AO |  |
| MIR5739 |  |
| MIR6512 |  |
| MIR6733 |  |
| MIR6799 |  |
| SNORA99 |  |
| ENSG00000258117 |  |
| LOC101927394 |  |
| MIR5692A2 |  |
| MIR6825 |  |
| LINC03056 |  |
| MIR11401 |  |
| ENSG00000275327 |  |
| piR-32051 |  |
| ENSG00000204584 |  |
| ENSG00000271858 |  |
| LOC110121280 |  |
| piR-36318 |  |
| LOC110120689 |  |
| LOC110121281 |  |
| LOC111365225 |  |
| piR-34736 |  |
| piR-43607 |  |
