# Supplementary material for: The crosstalk between metabolic reprogramming and epithelial-mesenchymal transition and their synergistic roles in distant metastasis in breast cancer
Source: Medicine (Baltimore). 2024 Jun 14;103(24):e38462. doi: 10.1097/MD.0000000000038462 (PMC11175907; doi:10.1097/MD.0000000000038462)
Supplement: Supplementary file 2 [file medi-103-e38462-s002.docx]

**Supplementary Table 2 The significant results of univariate Cox analysis of MR genes**

| **ID** | **Hazard Ratio (HR)** | **HR.95L** | **HR.95H** | **pvalue** |
| --- | --- | --- | --- | --- |
| TXNIP | 0.999875 | 0.99981 | 0.9999395 | 0.000153 |
| PNPLA2 | 0.999503 | 0.999243 | 0.9997633 | 0.000182 |
| CYCS | 1.000155 | 1.000069 | 1.0002405 | 0.000399 |
| STX3 | 1.000898 | 1.000398 | 1.0013974 | 0.000424 |
| HIF1A | 1.000176 | 1.000078 | 1.0002744 | 0.000453 |
| PPARD | 1.000974 | 1.000412 | 1.0015368 | 0.000687 |
| PFKFB3 | 1.000356 | 1.000146 | 1.000566 | 0.000905 |
| TPI1 | 1.00008 | 1.000032 | 1.0001266 | 0.000947 |
| NRAS | 1.001897 | 1.00077 | 1.0030241 | 0.000962 |
| CCL2 | 0.999469 | 0.999144 | 0.9997933 | 0.001337 |
| PYCR1 | 1.000499 | 1.000189 | 1.0008095 | 0.001617 |
| NDUFA4 | 1.000135 | 1.000051 | 1.0002186 | 0.00162 |
| OPA1 | 1.001118 | 1.000422 | 1.0018143 | 0.001643 |
| NDUFA6 | 1.000206 | 1.000074 | 1.0003378 | 0.002159 |
| ANGPTL4 | 1.000572 | 1.000204 | 1.0009392 | 0.002292 |
| NPC1 | 1.000668 | 1.000238 | 1.0010972 | 0.002296 |
| PDX1 | 0.990693 | 0.98466 | 0.9967625 | 0.002695 |
| CLPB | 1.002384 | 1.000823 | 1.0039464 | 0.002741 |
| PGAM1 | 1.000157 | 1.000054 | 1.0002595 | 0.002759 |
| BMP6 | 0.996468 | 0.994139 | 0.9988015 | 0.003031 |
| NR1H3 | 0.998638 | 0.997736 | 0.9995403 | 0.003103 |
| MAOA | 0.999489 | 0.999143 | 0.9998349 | 0.003788 |
| CD44 | 0.99986 | 0.999762 | 0.9999569 | 0.004688 |
| STAT3 | 0.999797 | 0.999655 | 0.9999389 | 0.005047 |
| NFKB1 | 0.999021 | 0.998336 | 0.9997057 | 0.005083 |
| HLA-B | 0.999971 | 0.99995 | 0.9999912 | 0.005203 |
| VEGFA | 1.000166 | 1.000049 | 1.0002829 | 0.005405 |
| PINK1 | 0.998985 | 0.998265 | 0.9997063 | 0.005815 |
| BRCA1 | 1.001563 | 1.000427 | 1.0027005 | 0.007007 |
| LDHA | 1.000054 | 1.000015 | 1.0000936 | 0.007138 |
| HCFC1 | 0.998826 | 0.997967 | 0.9996849 | 0.007399 |
| B2M | 0.999971 | 0.99995 | 0.9999931 | 0.009763 |
| SP1 | 0.99533 | 0.991786 | 0.9988874 | 0.010125 |
| MMP1 | 1.000128 | 1.00003 | 1.0002253 | 0.010485 |
| ABCC1 | 1.000503 | 1.000117 | 1.0008893 | 0.010595 |
| FOXM1 | 1.000404 | 1.000092 | 1.0007159 | 0.011103 |
| SHMT2 | 1.000203 | 1.000046 | 1.0003608 | 0.011459 |
| KCNJ5 | 0.99694 | 0.994575 | 0.9993119 | 0.011478 |
| EZH2 | 1.000584 | 1.00013 | 1.0010392 | 0.01174 |
| CPS1 | 1.000511 | 1.000113 | 1.0009087 | 0.011788 |
| CD38 | 0.998922 | 0.998077 | 0.9997682 | 0.012536 |
| PGK1 | 1.00007 | 1.000015 | 1.0001242 | 0.012656 |
| HPRT1 | 1.000206 | 1.000044 | 1.0003678 | 0.012923 |
| CDKN3 | 1.000684 | 1.000143 | 1.0012259 | 0.013202 |
| HNF4A | 0.996419 | 0.993585 | 0.9992612 | 0.013567 |
| TNFRSF11B | 0.996822 | 0.994303 | 0.9993475 | 0.013682 |
| GBE1 | 1.000419 | 1.000086 | 1.000753 | 0.013704 |
| GCK | 0.984422 | 0.972207 | 0.9967901 | 0.013715 |
| HEXA | 0.999222 | 0.9986 | 0.9998443 | 0.014293 |
| ERCC2 | 1.002584 | 1.000511 | 1.004662 | 0.014552 |
| LONP1 | 1.000496 | 1.000097 | 1.0008963 | 0.014935 |
| GSTO1 | 1.000179 | 1.000032 | 1.0003251 | 0.016703 |
| PGM1 | 1.000215 | 1.000037 | 1.0003933 | 0.017767 |
| SOCS1 | 0.996616 | 0.993817 | 0.9994217 | 0.018118 |
| GNPAT | 1.000316 | 1.000054 | 1.0005785 | 0.018277 |
| CREBBP | 1.000699 | 1.000118 | 1.0012803 | 0.018344 |
| SPTLC1 | 1.000402 | 1.000067 | 1.0007363 | 0.018599 |
| TFAM | 0.998322 | 0.996924 | 0.9997213 | 0.018776 |
| ALDH1A1 | 0.998775 | 0.99775 | 0.9998004 | 0.019224 |
| EIF2S1 | 1.000331 | 1.000054 | 1.0006085 | 0.019287 |
| ATG7 | 0.997334 | 0.995101 | 0.9995716 | 0.019561 |
| CPOX | 1.001418 | 1.000226 | 1.0026104 | 0.019701 |
| PAICS | 1.000194 | 1.000028 | 1.00036 | 0.021983 |
| GFPT1 | 1.000262 | 1.000035 | 1.0004884 | 0.023511 |
| PDHX | 1.000431 | 1.000056 | 1.000805 | 0.024091 |
| MTHFR | 0.991027 | 0.983272 | 0.9988422 | 0.024512 |
| REN | 0.994348 | 0.989438 | 0.9992822 | 0.024814 |
| DNMT3B | 1.000914 | 1.000114 | 1.0017146 | 0.025094 |
| PGR | 0.998359 | 0.996914 | 0.9998061 | 0.026254 |
| CPT1A | 1.000198 | 1.000022 | 1.0003739 | 0.027248 |
| MTHFD1 | 1.000472 | 1.000052 | 1.0008916 | 0.02761 |
| BCKDHB | 0.997796 | 0.99583 | 0.9997655 | 0.028301 |
| ATIC | 0.999664 | 0.999362 | 0.9999654 | 0.028907 |
| SOAT1 | 1.001523 | 1.000156 | 1.0028912 | 0.028952 |
| SDHA | 0.999553 | 0.999152 | 0.9999547 | 0.029178 |
| IFNG | 0.996593 | 0.99353 | 0.9996651 | 0.02976 |
| HSD17B10 | 1.00018 | 1.000017 | 1.0003433 | 0.030117 |
| AK2 | 0.999524 | 0.999092 | 0.9999567 | 0.031079 |
| ASNS | 1.000236 | 1.000021 | 1.0004504 | 0.031101 |
| SLC25A13 | 1.001155 | 1.000104 | 1.0022074 | 0.031173 |
| MAPK1 | 1.000654 | 1.000059 | 1.0012497 | 0.031329 |
| NDUFS2 | 1.000146 | 1.000013 | 1.0002802 | 0.032083 |
| HSPG2 | 1.000326 | 1.000027 | 1.000624 | 0.032393 |
| SIRT6 | 0.998083 | 0.996313 | 0.9998562 | 0.034113 |
| TXN | 1.000103 | 1.000007 | 1.0001982 | 0.034525 |
| MTAP | 0.997943 | 0.996016 | 0.9998735 | 0.036771 |
| AASS | 0.996523 | 0.993265 | 0.9997922 | 0.037131 |
| UQCRB | 1.000053 | 1.000003 | 1.0001036 | 0.037157 |
| SETD2 | 1.000395 | 1.000021 | 1.0007693 | 0.03843 |
| MTHFD2 | 1.000188 | 1.000009 | 1.0003663 | 0.039039 |
| PDHB | 1.000153 | 1.000008 | 1.0002985 | 0.039187 |
| ACSL4 | 1.002799 | 1.000125 | 1.0054789 | 0.040162 |
| ALDH7A1 | 0.999495 | 0.999011 | 0.9999787 | 0.040739 |
| SLC25A3 | 0.999895 | 0.999793 | 0.9999957 | 0.041016 |
| ABHD5 | 0.998014 | 0.996095 | 0.9999356 | 0.042808 |
| PIK3R1 | 0.999652 | 0.999312 | 0.9999911 | 0.044307 |
| AMPD2 | 1.000391 | 1.000007 | 1.0007759 | 0.045941 |
| ABCD1 | 0.998604 | 0.997233 | 0.999976 | 0.046135 |
| FTO | 0.999737 | 0.999478 | 0.9999958 | 0.046423 |
| HSD11B1 | 0.998454 | 0.996927 | 0.9999844 | 0.047715 |
| DNMT3A | 0.997632 | 0.995279 | 0.9999907 | 0.049109 |
|  |  |  |  |  |

HR.95L --- The lower limit of the 95% confidence interval

HR.95H --- The upper limit of the 95% confidence interval
