# Supplementary material for: The crosstalk between metabolic reprogramming and epithelial-mesenchymal transition and their synergistic roles in distant metastasis in breast cancer
Source: Medicine (Baltimore). 2024 Jun 14;103(24):e38462. doi: 10.1097/MD.0000000000038462 (PMC11175907; doi:10.1097/MD.0000000000038462)
Supplement: Supplementary file 3 [file medi-103-e38462-s003.docx]

**Supplementary Table 3 The significant results of univariate Cox analysis of EMT genes**

| **ID** | **Hazard Ratio (HR)** | **HR.95L** | **HR.95H** | **pvalue** |
| --- | --- | --- | --- | --- |
| AURKA | 1.000936 | 1.000453 | 1.001418 | 0.000143 |
| CDK2 | 1.0016 | 1.000765 | 1.002434 | 0.00017 |
| CEMIP | 1.001077 | 1.000501 | 1.001654 | 0.000247 |
| HIF1A | 1.000176 | 1.000078 | 1.000274 | 0.000453 |
| ENO2 | 1.000548 | 1.000234 | 1.000863 | 0.000634 |
| BUB1B | 1.001071 | 1.000444 | 1.001698 | 0.000807 |
| JAK2 | 0.997139 | 0.995467 | 0.998815 | 0.000825 |
| NRAS | 1.001897 | 1.00077 | 1.003024 | 0.000962 |
| PTTG1 | 1.000214 | 1.000084 | 1.000343 | 0.001232 |
| CCNB1 | 1.000714 | 1.000279 | 1.00115 | 0.001311 |
| CCL2 | 0.999469 | 0.999144 | 0.999793 | 0.001337 |
| LOXL2 | 1.00021 | 1.00008 | 1.00034 | 0.001585 |
| CDK1 | 1.000515 | 1.000194 | 1.000837 | 0.001668 |
| CCNB2 | 1.000416 | 1.000149 | 1.000684 | 0.00229 |
| ITGA5 | 1.000888 | 1.000309 | 1.001468 | 0.002638 |
| BTRC | 0.996241 | 0.993769 | 0.998719 | 0.002963 |
| BMP6 | 0.996468 | 0.994139 | 0.998802 | 0.003031 |
| PCNA | 1.000253 | 1.000084 | 1.000421 | 0.00326 |
| POSTN | 1.00004 | 1.000012 | 1.000068 | 0.004671 |
| CD44 | 0.99986 | 0.999762 | 0.999957 | 0.004688 |
| XPO1 | 1.00028 | 1.000085 | 1.000474 | 0.00478 |
| STAT3 | 0.999797 | 0.999655 | 0.999939 | 0.005047 |
| NFKB1 | 0.999021 | 0.998336 | 0.999706 | 0.005083 |
| VEGFA | 1.000166 | 1.000049 | 1.000283 | 0.005405 |
| SMURF2 | 1.000669 | 1.000194 | 1.001144 | 0.005792 |
| JAG1 | 1.000215 | 1.000062 | 1.000369 | 0.006058 |
| NDRG1 | 1.000038 | 1.000011 | 1.000066 | 0.006687 |
| HDAC3 | 0.998766 | 0.997874 | 0.999659 | 0.006747 |
| BRCA1 | 1.001563 | 1.000427 | 1.0027 | 0.007007 |
| CDC27 | 1.001767 | 1.000468 | 1.003069 | 0.007681 |
| IL17A | 0.995231 | 0.991676 | 0.998799 | 0.008838 |
| SP1 | 0.99533 | 0.991786 | 0.998887 | 0.010125 |
| FOXM1 | 1.000404 | 1.000092 | 1.000716 | 0.011103 |
| EZH2 | 1.000584 | 1.00013 | 1.001039 | 0.01174 |
| ITGB1 | 1.000101 | 1.000022 | 1.000179 | 0.012377 |
| CDH2 | 1.000136 | 1.000029 | 1.000244 | 0.012675 |
| CDKN3 | 1.000684 | 1.000143 | 1.001226 | 0.013202 |
| HNF4A | 0.996419 | 0.993585 | 0.999261 | 0.013567 |
| SNAI2 | 1.000411 | 1.000084 | 1.000738 | 0.013768 |
| ERCC2 | 1.002584 | 1.000511 | 1.004662 | 0.014552 |
| BIRC5 | 1.000301 | 1.000058 | 1.000545 | 0.015346 |
| PKMYT1 | 1.001024 | 1.000194 | 1.001854 | 0.015532 |
| WNT4 | 0.997698 | 0.99583 | 0.999568 | 0.015885 |
| CCND3 | 0.999212 | 0.998567 | 0.999857 | 0.016686 |
| SOX4 | 1.000072 | 1.000013 | 1.000131 | 0.017034 |
| MCM2 | 1.000235 | 1.000042 | 1.000429 | 0.017308 |
| LINC00472 | 0.995146 | 0.991162 | 0.999145 | 0.01742 |
| CREBBP | 1.000699 | 1.000118 | 1.00128 | 0.018344 |
| TOP2A | 1.000178 | 1.000028 | 1.000328 | 0.019907 |
| TGFBR1 | 0.99512 | 0.991016 | 0.999242 | 0.020356 |
| TGFB2 | 0.995887 | 0.992274 | 0.999513 | 0.02622 |
| SIX1 | 1.001645 | 1.000161 | 1.00313 | 0.029758 |
| PLAU | 1.000208 | 1.000019 | 1.000397 | 0.030816 |
| MAPK1 | 1.000654 | 1.000059 | 1.00125 | 0.031329 |
| FAP | 1.000216 | 1.000018 | 1.000413 | 0.032223 |
| PTK2 | 1.000192 | 1.000012 | 1.000371 | 0.036878 |
| HSPB1 | 1.000026 | 1.000001 | 1.00005 | 0.039028 |
| TWIST1 | 1.000439 | 1.00002 | 1.000858 | 0.040174 |
| TEAD4 | 1.000524 | 1.000018 | 1.00103 | 0.042586 |
| NME1 | 1.000056 | 1.000002 | 1.00011 | 0.043214 |
| CDK5 | 1.001163 | 1.000035 | 1.002292 | 0.043299 |
| CDC20 | 1.000269 | 1.000008 | 1.00053 | 0.043714 |
| PIK3R1 | 0.999652 | 0.999312 | 0.999991 | 0.044307 |
| HSPA5 | 0.999889 | 0.999779 | 1 | 0.049634 |
|  |  |  |  |  |

HR.95L --- The lower limit of the 95% confidence interval

HR.95H --- The upper limit of the 95% confidence interval
