# Supplementary material for: The crosstalk between metabolic reprogramming and epithelial-mesenchymal transition and their synergistic roles in distant metastasis in breast cancer
Source: Medicine (Baltimore). 2024 Jun 14;103(24):e38462. doi: 10.1097/MD.0000000000038462 (PMC11175907; doi:10.1097/MD.0000000000038462)
Supplement: Supplementary file 4 [file medi-103-e38462-s005.docx]

The formula for the calculation of Metastasis Score

Metastasis Score = -0.385390*SP1 + 0.374615*HPRT1 - 0.229742*NR1H3 + 0.054029*GBE1 + 0.409407*GFPT1 - 0.367299*BCKDHB - 0.430774*CD44 -0.113167*ALDH1A1 - 0.714278*CCL2 + 1.083416*ANGPTL4 + 0.518780*SMURF2 + 0.092299*HSPB1 + 0.374084*NDRG1
